# Museomics and phylogenomics with protein-encoding ultraconserved elements illuminate the evolution of life history and phallic morphology of flesh flies (Diptera: Sarcophagidae)

**DOI:** 10.1186/s12862-021-01797-7

**Published:** 2021-04-28

**Authors:** Eliana Buenaventura

**Affiliations:** 1 Center for Integrative Biodiversity Discovery, Museum für Naturkunde, Leibniz Institute for Evolution and Biodiversity Science, Invalidenstraße 43, 10115 Berlin, Germany; 2 National Museum of Natural History, Smithsonian Institution, Washington, DC 20013 USA

**Keywords:** Predation, Sarcosaprophagy, Coprophagy, Targeted enrichment, Phylogenomics, Oestroidea, Sarcophagidae, Ultraconserved elements, Rapid radiation

## Abstract

**Background:**

The common name of the Flesh flies (Sarcophagidae) usually relates them with organisms feeding on decomposing organic matter, although the biology of one of the largest radiations among insects also includes predation, coprophagy, and even kleptoparasitism. The question of whether the ancestor of all sarcophagids was a predator or a decomposer, or in association to which host have sarcophagids evolved, has thus always piqued the curiosity of flesh fly specialists. Such curiosity has often been hindered by both the impossibility of having a well-supported phylogeny of Sarcophagidae and its sister group to trace live habits and the scarcity of information on the biology of the group. Using a phylogenomic dataset of protein-encoding ultraconserved elements from representatives of all three subfamilies of Sarcophagidae as ingroup and a large Calyptratae outgroup, a robust phylogenetic framework and timescale are generated to understand flesh fly systematics and the evolution of their life histories.

**Results:**

The evolutionary history for Sarcophagidae reconstructed here differs considerably from previous hypotheses. Within subfamily Sarcophaginae, a group of predatory flies, including genera *Lepidodexia* and *Boettcheria*, emerged as sister-group to the rest of Sarcophaginae. The genera *Oxysarcodexia*, *Ravinia*, and *Tricharaea*, long considered archaic and early-branching coprophagous and sarcosaprophagous lineages, were found nested well within the Sarcophaginae as sister-group to the sarcosaprophagous *Microcerella*. Predation on invertebrates is suggested as the ancestral and dominant strategy throughout the early evolution of flesh flies. Several transitions from predation to sarcosaprophagy and coprophagy occur across the sarcophagid phylogenetic tree, in contrast with almost no transitions from sarcosaprophagy or coprophagy to predatory habits. Regarding the morphological evolution of flesh flies, there might be a concerted evolution of male genitalia traits, such as the phallotrema position and the juxta, or the vesica and the folding of the phallotrema. One diversification rate shift was inferred in the evolution of sarcophagids, which is related to the origin of genus *Sarcophaga*.

**Conclusions:**

This study has a significant impact on understanding sarcophagid evolution and highlights the importance of having a robust phylogenetic framework to reconstruct the ancestral character state of biological and morphological characters. I discuss the evolution of life histories of the family in relation to their hosts or substrates and outline how sarcosaprophagy, coprophagy, and kleptoparasitism behavior on various hosts may have evolved from predation on invertebrates. This study provides a phylogenetic framework for further physiological and comparative genomic work between predatory, sarcosaprophagous, coprophagous, and kleptoparasitic lineages, which could also have significant implications for the evolution of diverse life histories in other Diptera.

**Supplementary Information:**

The online version contains supplementary material available at 10.1186/s12862-021-01797-7.

## Background

The flesh flies (Sarcophagidae, ~ 3000 species) have been recognized as one of the largest insect radiations [1] with an age estimated at 23 Ma [2] and a myriad of different life habits, whose evolutionary patterns are not yet explained. The common name of flesh flies usually relates them directly with organisms feeding on decomposing organic matter, although their biology is one of the most diverse among insects. In fact, they constitute a diverse assemblage of flies that vary considerably in their biological requirements for larval food resources and feeding habits. Published data of the sarcophagid larval biology document the complexities of their nutritional options and biological relationships [3, 4], which include parasites, parasitoids on various hosts, kleptoparasites (inquilines), predators, sarcosaprophages, and coprophages [5]. Some species are habitat- or host-specific and accurately placed in one of these categories. Others show a more opportunistic or generalist approach, utilizing instead a variety of hosts or several different food sources. However, for many of these species, precise ecological requirements remain essentially unknown. Thus, limited biological information on the group and little understanding of the phylogenetic relationships of sarcophagids limit inferences on the evolutionary pattern of this diverse ecology and its impacts on flesh-fly diversification.

Sarcophagidae species are organized into three subfamilies, Miltogramminae, Paramacronychiinae, and Sarcophaginae [6]. The most diverse subfamily is Sarcophaginae with around 2,000 species in 46 genera [7] that have colonized almost all terrestrial ecosystems. Only few Sarcophaginae species occur in subarctic regions but most are found in tropical areas, showing a peak of diversity in the Neotropical region [8]. Only the three genera of Sarcophaginae *Blaesoxipha* Loew, *Ravinia* Robineau-Desvoidy, and *Sarcophaga* Meigen have been able to extend their distribution out of the Americas, although the latter one also colonized Afro-Eurasia and Australasia/Oceania and diversified becoming the largest radiation within this subfamily with almost 900 species worldwide [9–11].

Flesh flies of Sarcophaginae seem to be a versatile group able to extend their distributions and dominate different feeding substrates. In urban environments, some flesh flies indistinctly feed on garbage and corpses of animals and humans becoming mechanical vectors of important diseases of great importance for public health [12, 13]. Their affinity for decomposing matter of human corpses makes them useful as indicators of time and place of death in forensic investigations [14, 15]. This preference for decomposing organic matter is defined as sarcosaprophagy, which is different from coprophagy, as carrion and dung produce different profiles of volatile organic compounds. Despite of these differences in volatile profiles, some sarcophagid species are able to feed both on carrion and dung. Other sarcophagids seem to have evolved more specialized relationships with their feeding substrates, which sometimes are not as ‘passive’ as carrion and dung, but consist of a broad range of living organisms targeted by flesh flies as preys or hosts. Sarcophagids are able to develop on terrestrial gastropods and arthropods (mostly insects) including millipedes, scorpions, beetles, grasshoppers, cockroaches, and mantids either as predators or parasites [5, 16, 17]. Even more specialized sarcophagids behave as kleptoparasitoids on soil-nesting wasps, bees and ants [18], a strategy where some species have adapted to usurp other species’ resources instead of collecting their own. Carrion and dung-flies provide ecosystem services such as nutrient recycling [19–21], which are essential for the sustainability and well being of urban, rural, and wild ecosystems, while the ecosystem impact and services of predator and kleptoparasitic flies is entirely unclear.

In contrast to other widespread fly groups, flesh flies have been largely neglected in phylogenetic studies. Still, the most representative phylogenetic hypotheses included only a small taxon sampling representing less than one third of the flesh fly species [9, 18, 22–﻿24﻿] or they were based on datasets of maximum nine genes reporting very low statistical support [18, 22, 23, ﻿25﻿]. As a result, basic knowledge on phylogenetic relationships is lacking for most flesh fly lineages and their evolutionary history remains unraveled. Specifically, the homology characterization of male terminalia structures, which are essential for species recognition in diversity surveys and species delimitation analyses, and the evolution of larval feeding substrates and habits are still little studied.

Large-scale evolutionary studies are currently dramatically benefitting from modern genomic-based approaches, yet no attempts have been made to apply these methods to elucidate the evolution of sarcophagids. The most popular approaches for understanding biological diversification in time and space include two different strategies of reduced-representation of genome using targeted enrichment, i.e., anchored hybrid enrichment (AHE) [9, 26, 27] and ultraconserved elements (UCE) [28–33]. Targeted enrichment is especially popular as it produces large amounts of molecular data from highly fragmented DNA and/or sub-optimally preserved samples such as those coming from museum specimens [34, 35]. Particularly, studies using UCEs have capitalized on museum specimens [30, 31, 33–37] and set the stage for the development of an entire field known as “museomics”. Using protein-encoding UCEs brings the best of two major approaches of reduced representation sequencing strategies, i.e., transcriptomes and UCE. Thus, UCEs can be applied to degraded DNA while transcriptomes ensure targeting protein-encoding genomic regions. The use of protein-encoding UCEs to resolve phylogenetic relationships within dipterans has only started to be explored [33], and as Sarcophagidae is one the largest radiations of flies that account for the majority of fly life on Earth [38], they constitute an interesting taxon to be studied with the combined use of the UCE targeted-enrichment method and massively parallel DNA sequencing technologies.

Thus, I aimed to reconstruct the phylogenetic relationships of flesh flies and provide a discussion of the implications for morphological and biological character evolution. In an effort to resolve possible incongruences, I assembled a novel UCE-based dataset of Sarcophagidae and a large representation of related outgroup species of Oestroidea and other Calyptratae, and assessed the robustness of phylogenetic estimates using concatenation and gene tree-based approaches. I additionally conducted an analysis of diversification rates and a reconstruction of ancestral character states (i.e., male terminalia characters and larval natural history) of the major lineages of flesh flies and Oestoidea outgroups.

## Results

### UCE probes and capture results

All DNA extractions from pinned museum specimens (= 24), specimens preserved in 96% ethanol (= 66) and liquid nitrogen (= 19) succeeded. Similarly, all existing DNA aliquots (= 32) had ample well-preserved DNA (above 1 ng/µL) for the present study. Thus, 141 DNA extractions were enriched using the UCE probes and sequenced on two Illumina Hi-Seq lanes. 17% of the sampled taxa consisted of pinned museum specimens (3–54 years old), of which 100% resulted in successful UCE enrichment. I recovered more UCE loci than the average for 11 pinned, dried specimens with age range of 16–48 years. See Additional file 1 to compare specimen age *versus* total DNA extracted and UCEs captured coded for preservation method.

Sequencing of libraries produced an average of 2,374,209 raw paired-end reads per sample. Trinity assembled reads into 1070–75,722 contigs with average of 9,608 contigs assembled per sample. These contigs had average lengths of 242.9–516.3 bp. From the total assembled contigs, a total of 2018 UCE loci out of 2,581 UCE targets were recovered across all taxa with an average of 1214 UCE loci per sample and average lengths ranging from 230.5 to 625 bp. Summary results of empirically generated UCE data processed are presented in Additional file 1.

I analyzed datasets containing 64–2018 UCE loci from 141 representatives, including 110 ingroup species of Sarcophagidae and 31 outgroup species from 10 Calyptratae families, with a total concatenated aligned length ranging between 20 and 551 kB.

### Phylogenetic results

**Fig. 1:**
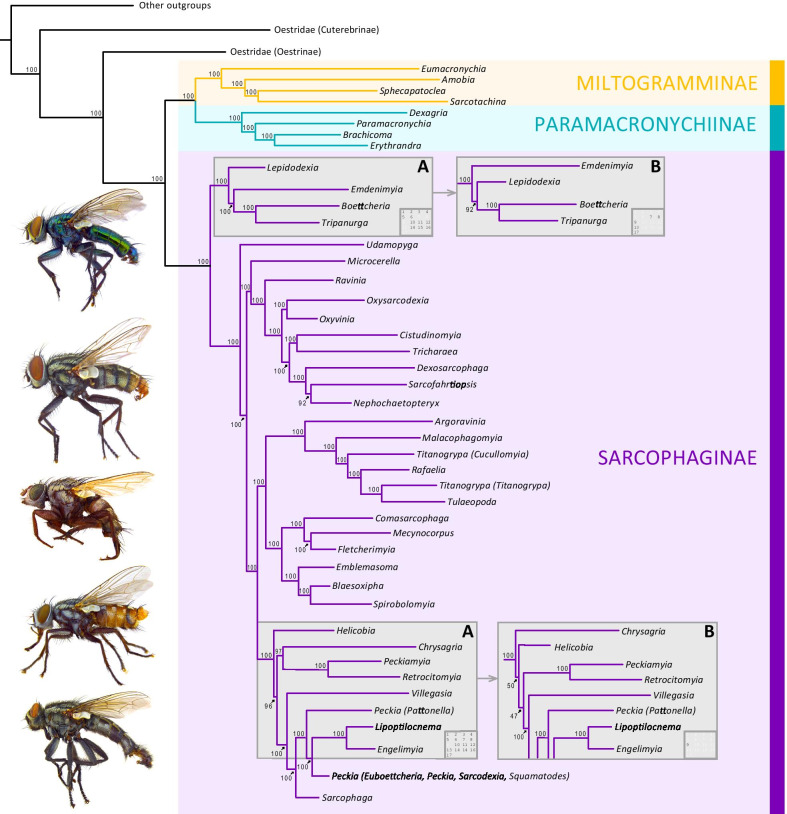
Genus-level summary of phylogeny of Sarcophagidae. The background topology is shared among all analyses. **a** Topology produced from datasets having 1271–2018 UCE loci. **b** Topology produced from datasets having 59 UCE loci coded as nucleotides and from an intermediate topology between topologies A and B produced by three datasets having 288–936 UCE loci coded as nucleotides or amino acids. Bootstrap support values are shown on branches. Inset squares indicate analyses producing A and B topologies (see Table 1 for analysis numbers). (For interpretation of the references to color in this figure legend, the reader is referred to the web version of this article)

**Fig. 2:**
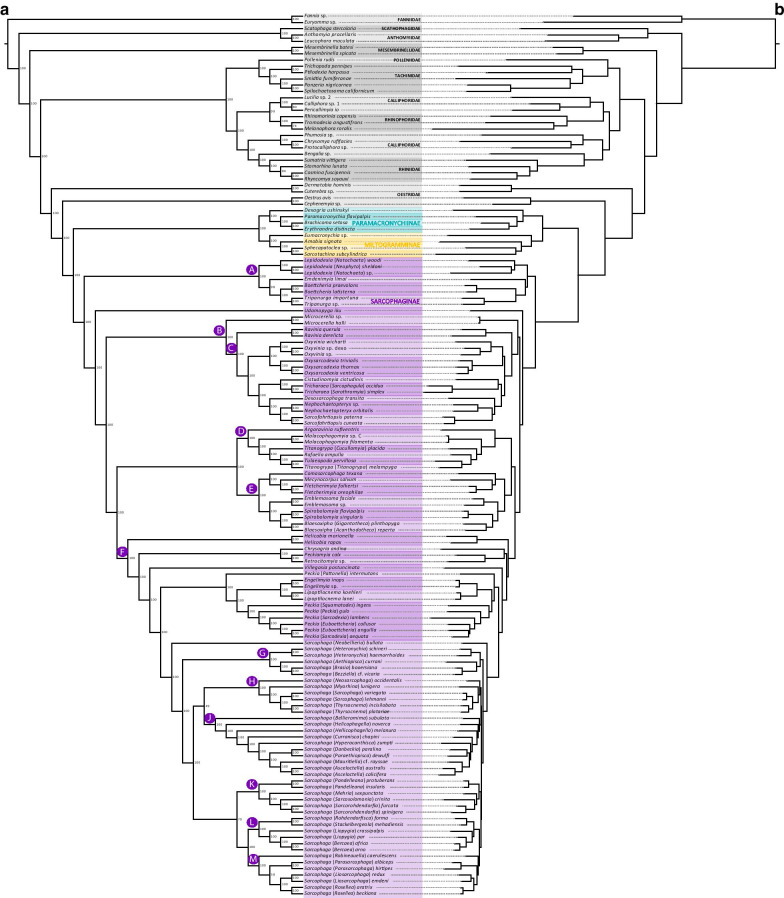
Phylogeny of Sarcophagidae. Species tree estimated by ASTRAL-III analysis of the dataset having 2018 UCE loci coded as nucleotides (topology A) using SWSC-EN partitioning scheme. Local posterior probabilities are shown in front of each node. Main tree is displayed as cladogram for clarity of relationships (**a**, left panel) and phylogram includes information on branch lengths (**b**, right panel). Current subfamily assignments are indicated. Capital letters indicate nodes discussed in the main text. (For interpretation of the references to color in this figure legend, the reader is referred to the web version of this article)

Phylogenetic relationships for Sarcophagidae and 10 other Calyptratae fly families were inferred from 17 datasets having varying loci occupancy and coded as nucleotides and amino acids using a concatenated ML approach as well as by reconstructing a species tree estimated from UCE gene trees. Both methods returned two topologies largely congruent at genus- and species-level relationships, but topology A received higher branch support (BS, Bootstrap support = 96–100) than topology B (Fig. 1a, b). Most datasets, i.e., 12 out of 17, returned the topology A (Table 1). Datasets producing topology A included 1271–2018 UCE loci (Fig. 1a), while only one dataset, which included 59 UCE loci coded as nucleotides, produced topology B (Fig. 1b). An alternative topology, which is an intermediate between topologies A and B, was produced by four datasets having 288–936 UCE loci coded as nucleotides or as amino acids (Fig. 1a, b). For illustration purposes, the most recurrently recovered topology (topology A), i.e., the coalescent-based tree reconstructed from the dataset containing 2018 UCE loci coded as nucleotides, which received strong statistical support, is depicted in Fig. 2.

**Table 1:** Summary of datasets and phylogenomic analyses

Analysis number	Datamatrix	Occupancy (%)	UCE loci	Sequence coding	Model	Analysis	Topology
1	10_nt_ml	0.10	2018	Nucleotides	GTRCAT	concat. RAxML	A
2	20_nt_ml	0.20	1778	Nucleotides	GTRCAT	concat. RAxML	A
3	30_nt_ml	0.30	1634	Nucleotides	GTRCAT	concat. RAxML	A
4	40_nt_ml	0.40	1524	Nucleotides	GTRCAT	concat. RAxML	A
5	50_nt_ml	0.50	1417	Nucleotides	GTRCAT	concat. RAxML	A
6	60_nt_ml	0.60	1271	Nucleotides	GTRCAT	concat. RAxML	A
7	70_nt_ml	0.70	936	Nucleotides	GTRCAT	concat. RAxML	int.
8	80_nt_ml	0.80	440	Nucleotides	GTRCAT	concat. RAxML	int.
9	90_nt_ml	0.90	59	Nucleotides	GTRCAT	concat. RAxML	B
10	10_nt_astral	0.10	2018	Nucleotides	GTR + F + I + G4	coales. ASTRAL-III	A
11	30_nt_astral	0.30	1634	Nucleotides	GTR + F + I + G4	coales. ASTRAL-III	A
12	60_nt_astral	0.60	1271	Nucleotides	GTR + F + I + G4	coales. ASTRAL-III	A
13	75_nt_astral	0.75	288	Nucleotides	GTR + F + I + G4	coales. ASTRAL-III	int.
14	10_aa_astral	0.10	2018	Amino acids	HIVw + F + I + G4	coales. ASTRAL-III	A
15	30_aa_astral	0.30	1634	Amino acids	HIVw + F + I + G4	coales. ASTRAL-III	A
16	60_aa_astral	0.60	1271	Amino acids	HIVw + F + I + G4	coales. ASTRAL-III	A
17	75_aa_astral	0.75	288	Amino acids	HIVw + F + I + G4	coales. ASTRAL-III	int.

Topologies have been grouped into A, B and ‘int’ for intermediate according to their phylogenetic relationships

Overall, datasets including more UCE loci (1271–2018) produced topologies that were more congruent among them, while datasets containing fewer UCE loci (59–936) (Table 1) produced topologies showing alternative relationships at genus- and subgenus-level. Similarly, UCE loci coded as amino acids, which by principle produce datasets with fewer characters, produced alternative topologies that were incongruent with the phylogenetic relationships recovered using datasets that included more characters. Resulting topologies from all of the analyses are included in Additional file 2.

Within Sarcophagidae, the subfamilies were recovered as monophyletic. Among the Sarcophagidae genera represented by two or more species, only the genera *Peckia* Robineau-Desvoidy and *Titanogrypa* Townsend were not recovered as monophyletic. Within the genus *Sarcophaga*, among subgenera represented by two or more species, only subgenera *Helicophagella* Enderlein and *Liopygia* Enderlein were recovered as paraphyletic in all analyses.

Subfamily-level relationships within Sarcophagidae received full support (BS = 100, local posterior probability (LPP) = 100) and were identical across all phylogenetic analyses, with Sarcophaginae as sister to (Miltogramminae + Paramacronychiinae). Regarding genus-level relationships, all analyses produced topologies that were largely congruent and well supported (BS = 89–100, LPP = 100) with two exceptions, as follows: (1) genus *Emdenimyia* Lopes as sister to (*Tripanurga* Brauer & Bergenstamm + *Boettcheria* Parker) in 12 out of 17 analyses (Fig. 1a) or to (*Lepidodexia* Brauer & Bergenstamm (*Tripanurga* + *Boettcheria*)) in 5 out of 17 analyses (Fig. 1b); (2) genus *Chrysagria* Townsend as sister to (*Peckiamyia* Dodge + *Retrocitomyia* Lopes) in 16 out of 17 analyses (Fig. 1a) or to (*Helicobia* Coquillett ((*Chrysagria* (*Peckiamyia* + *Retrocitomyia*)) + (remaining Sarcophaginae))) in 1 out of 17 analyses (Fig. 1b). Regarding subgenus- and species-level relationships, all analyses produced topologies that were largely congruent, with few exceptions, all of them within the genus *Sarcophaga*. Seven different topologies are reconstructed for this genus. One of these topologies is overall well supported and recurrent among analyses, which is depicted in Fig. 2. Differences among the seven topologies for *Sarcophaga* include (1) variable phylogenetic positions of clade H containing species of subgenera *Neosarcophaga* Shewell and *Myorhina* Robineau-Desvoidy (among others) and clade J containing subgenera *Bellieriomima* Rohdendorf and *Helicophagella* (among others) that are recovered as sister groups or as laddered sister-groups to the remaining *Sarcophaga*, and (2) the position of subgenus *Parasarcophaga* Johnston & Tiegs as sister to (*Robineauella* Enderlein (*Liosarcophaga* Enderlein + *Rosellea* Rohdendorf)) or to (*Liosarcophaga* + *Rosellea*) in clade M (Fig. 2, Additional file 2).

### Dating analysis

Using the function *makeChronosCalib* in the R package *ape*, I estimated the timescale of the evolution of Sarcophagidae. The estimation of ages used the topology A from the coalescent-based tree reconstructed from the dataset containing 2018 UCE loci and coded as nucleotides. The estimated age for the most recent common ancestor (MRCA) of the clade (Miltogramminae + Paramacronychiinae) is 19.6 Ma and for Sarcophaginae is 21.8 Ma, while the estimated age for the MRCA of the subfamily Miltogramminae is 7.8 Ma and for Paramacronychiinae is 14.9 Ma. The estimated age for the MRCA of genus *Sarcophaga*, the richest of the whole family, is 14.1 Ma. The Miocene was an important setting in generating the presently recognized generic diversity of Sarcophagidae, as the majority of genera appear to have evolved between 1 and 15 Ma. However, these age estimates include considerable uncertainty and should be read with caution, given the lack of fossils for sarcophagids and closely related fly families.

### *Ancestral character reconstruction of life history and male terminalia traits*

**Fig. 3:**
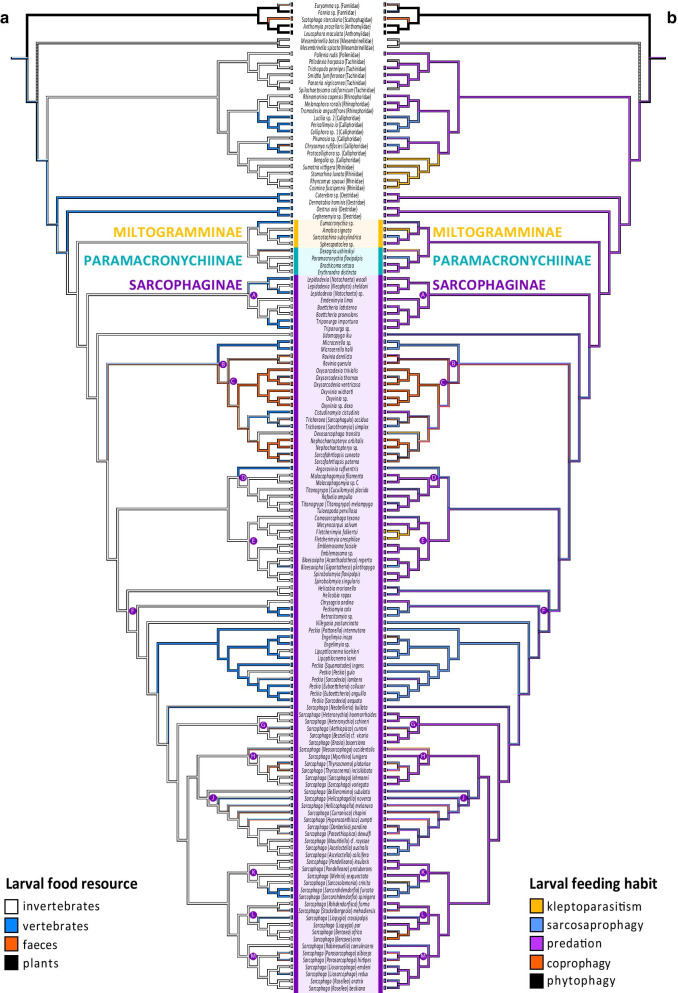
Evolution of sarcophagid life histories. Ancestral character state reconstructions for sarcophagid life histories using a parsimony-based ancestral character state reconstruction (ACR) on the ASTRAL-III species tree estimated from the dataset having 2018 UCE loci coded as nucleotides (topology A) in the Mesquite software. **a** Left panel shows ACR for larval food resource. **b** Right panel shows ACR for larval feeding habit. Character states are indicated at the bottom right and left of each panel. Current subfamily assignments are indicated. Capital letters indicate nodes discussed in the main text. (For interpretation of the references to color in this figure legend, the reader is referred to the web version of this article)

The ancestral character state reconstruction (ACR) for life-history traits using Mesquite suggests that phytophagy and predation are the most parsimonious states for the MRCA of Oestroidea, while the most parsimonious state for the MRCA of the clade containing the Oestroidea families Calliphoridae, Polleniidae, Rhiniidae, Rhinophoridae, and Tachinidae is predation on invertebrates (Fig. 3). The most parsimonious state for the MRCA of (Oestridae + Sarcophagidae) is predation on invertebrates or vertebrates. Similarly, the most parsimonious state for the MRCA of Sarcophagidae, and for each of its subfamilies, is predation on invertebrates. Within subfamily Sarcophaginae, several MRCAs have made use of other resources than invertebrates. For example, the most parsimonious state for the MRCA of the clade B is a diverse set of phenotypes including feces, invertebrates, and vertebrates as larval food resources and coprophagy, sarcosaprophagy and, predation as larval feeding habits. More specialized phenotypes are estimated within this clade, with coprophagy as the most parsimonious state for the MRCA of the clade (*Oxysarcodexia* Townsend + *Oxyvinia* Dodge). Similarly, a specialized phenotype is estimated for the clade containing genera *Engelimyia* Lopes, *Lipoptilocnema* Townsend, and *Peckia*, whose MRCA has sarcosaprophagy on vertebrates as the most parsimonious state. The most parsimonious state for the MRCA of the genus *Sarcophaga* includes predation and sarcosaprophagy on invertebrates.

**Fig. 4:**
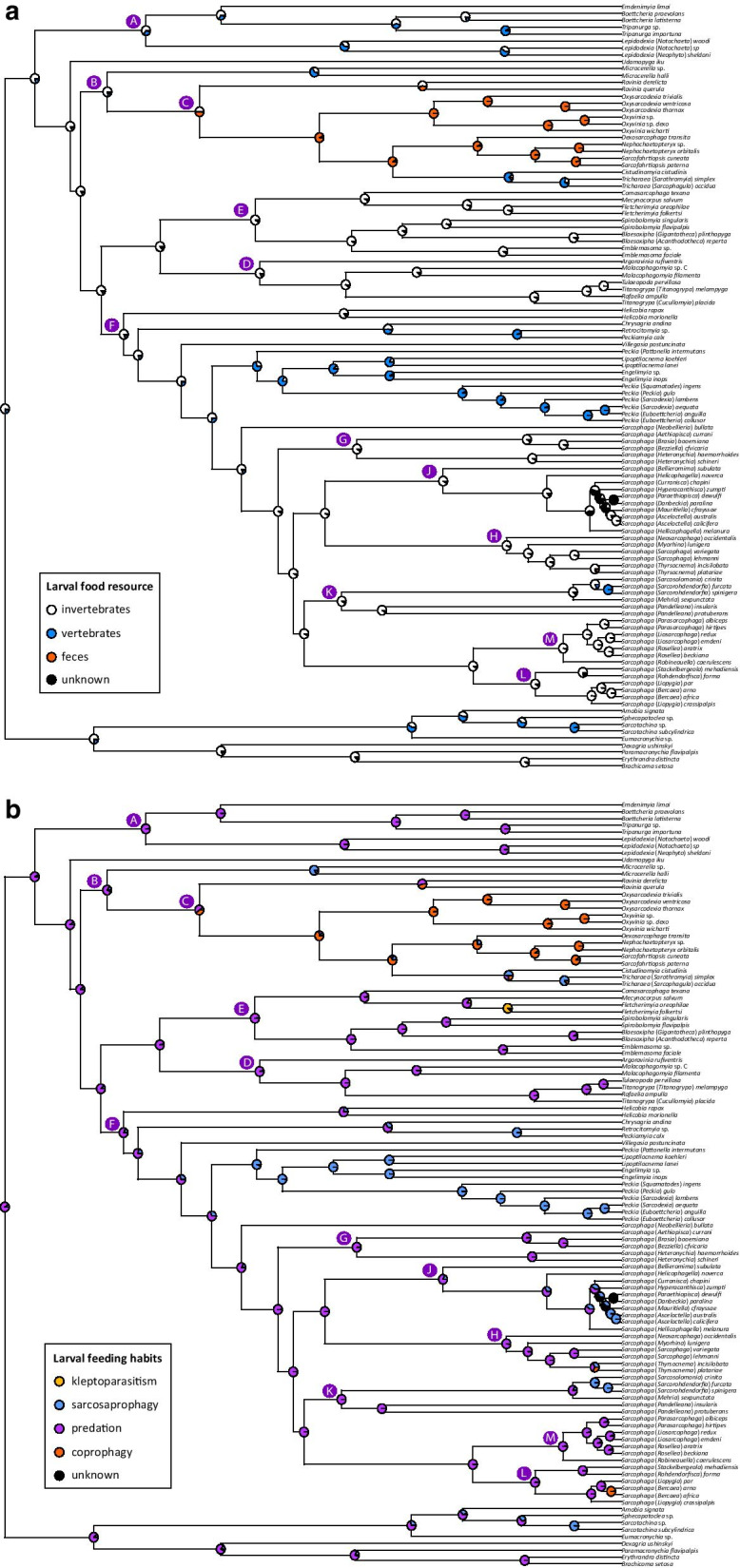
Evolution of sarcophagid life histories. Ancestral character state reconstructions for sarcophagid life histories using maximum likelihood and the *rayDISC* function in the R package *corHMM*. Only reconstructions of the best fitting models are shown. Pie proportions represent state probabilities estimated for each internal node. **a** Top panel shows ACR for larval food resource (model ARD). **b** Bottom panel shows ACR for larval feeding habits (model ER). Character states are indicated in insets inside each panel. Capital letters indicate nodes discussed in the main text. (For interpretation of the references to color in this figure legend, the reader is referred to the web version of this article)

In the *corHMM*-based ACR, the two evaluated models (ARD = all transition rates are allowed and are independently estimated; ER = all transitions among the specified number of rate classes are the same) provided a differential best fit to the life history and morphological characters (Additional file 3). I found stronger support for a MRCA of Sarcophagidae using invertebrates than vertebrates as larval food resource (Fig. 4a), and overwhelming support for a MRCA of Sarcophagidae with predation as larval feeding habit (Fig. 4b). There is further strong support for invertebrates as larval food resource of the MRCA of each of the Sarcophagidae subfamilies, and very strong support for predation as the larval feeding habit of the MRCA of Sarcophaginae while slightly weaker support for predation as the larval feeding habit of the MRCA of Miltogramminae and Paramacronychiinae. Within the Sarcophaginae, transitions in larval food resources are observed for the clade B (Fig. 4a). For example, the MRCA of this clade is possibly a predator on invertebrates, while the MRCA of genus *Microcerella* Macquart is a sarcosaprophage on vertebrates and the MRCA of the remaining taxa in clade C is possibly a predator or coprophagous. Similarly, the MRCA of the clade containing genera *Engelimyia*, *Lipoptilocnema*, *Peckia*, and *Sarcophaga* is most likely a predator on invertebrates, while the MRCA of the subclade containing genera *Engelimyia*, *Lipoptilocnema*, and *Peckia* is a sarcosaprophage on vertebrates. There is further strong support for the MRCA of genus *Sarcophaga* as a predator on invertebrates, with several subsequent transitions towards sarcosaprophagy on vertebrates and coprophagy. Thus, predominant sarcosaprophagy and coprophagy have evolved independently only a few times within the subfamily Sarcophaginae. Similarly, few taxa have adapted to a combination of life habits including coprophagy, sarcosaprophagy, and predation from a mostly predator ancestor. Such is the case of some species of subgenera of *Sarcophaga*, like *Bercaea* Robineau-Desvoidy, *Helicophagella*, *Liopygia*, *Neobellieria* Blanchard, *Parasarcophaga*, and *Thyrsocnema* Enderlein, which are subgenera usually having one widespread species with a variety of records as coprophage, sarcosaprophage and/or predator, while the remaining species of these subgenera have species with more specialized habits, but in many cases, species with less available information on their habits.

**Fig. 5:**
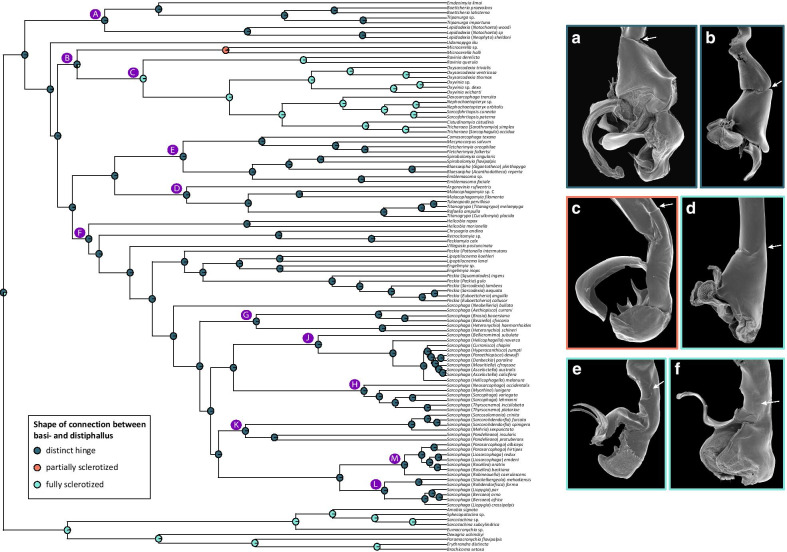
Evolution of the shape of connection between basi- and distiphallus of Sarcophagidae. Ancestral character state reconstructions for shape of connection between basi- and distiphallus using maximum likelihood and the *rayDISC* function in the R package *corHMM*. Only the reconstruction of the best fitting model (model ER) is shown. Pie proportions represent state probabilities estimated for each internal node. Character states are indicated in inset inside tree panel. Capital letters on the tree indicate nodes discussed in the main text. Insets at right showing the phallus in left lateral view with arrows indicating the connection between basi- and distiphallus of **a** *Lepidodexia* (*Notochaeta*) *woodi*; **b** *Udamopyga neivai*; **c** *Microcerella spinigena*; **d** *Ravinia* sp.; **e** *Oxysarcodexia angrensis*; **f** *Sarcofahrtiopsis* (*Pacatuba*) *matthewsi*. **a**, **c**, **d**, and **e** courtesy of Dr. M. Giroux. (For interpretation of the references to color in this figure legend, the reader is referred to the web version of this article)

**Fig. 6:**
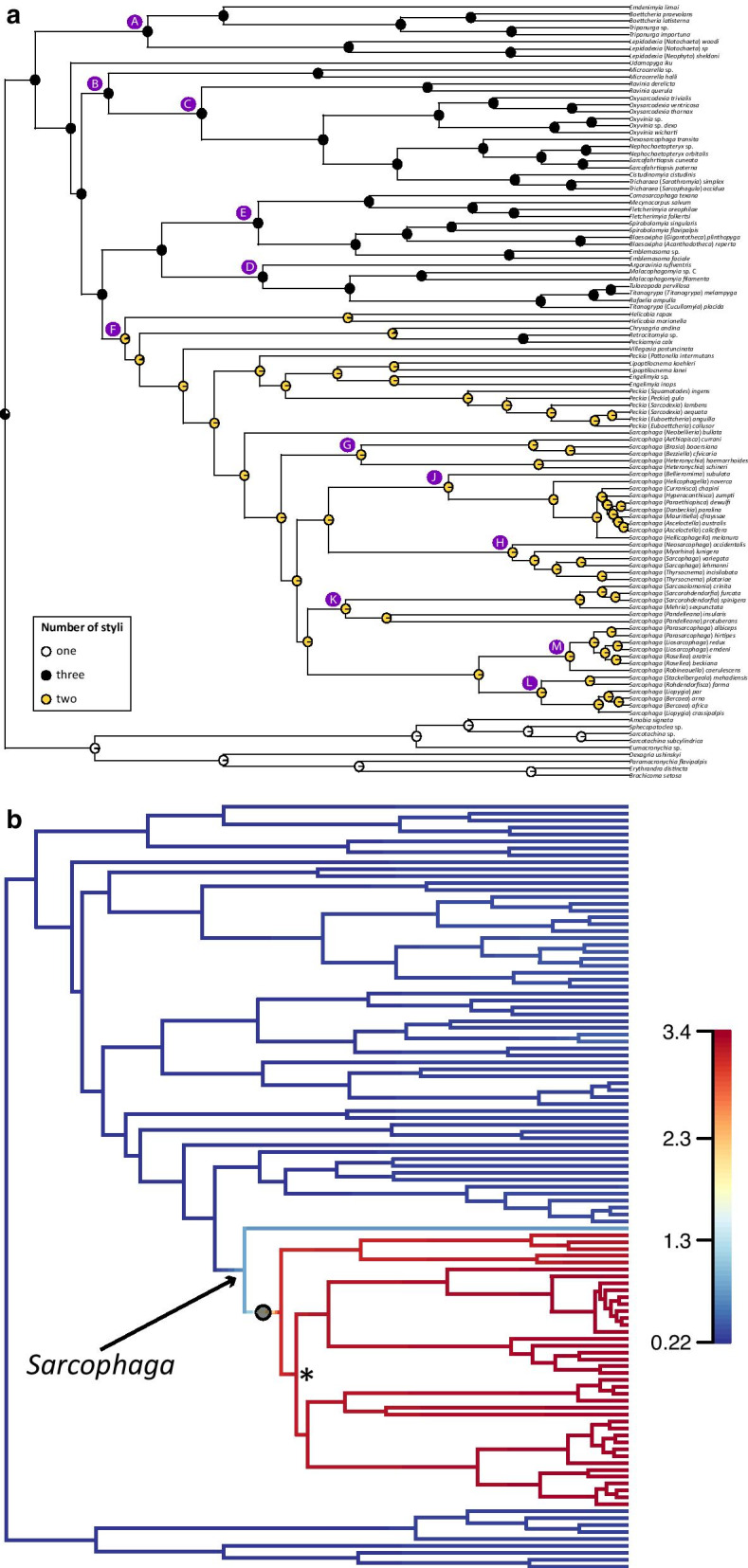
Evolution of the number of styli and diversification of Sarcophagidae. **a** Top panel shows ancestral character state reconstruction for the number of styli using maximum likelihood and the *rayDISC* function in the R package *corHMM*. Only reconstruction of the best fitting model (model ER) is shown. Pie proportions represent state probabilities estimated for each internal node. Character states are indicated in inset inside panel. Capital letters indicate nodes discussed in the main text. **b** BAMM analysis on the chronogram resulting from calibration analysis of the ASTRAL-III species tree estimated from the dataset having 2018 UCE loci coded as nucleotides (topology A) using *makeChronosCalib* in the R package *ape* and using clade-specific sampling probabilities (Additional file 15) to account for incomplete sampling based on species estimates. Phylorate graph showing the mean marginal posterior density of speciation rates, an increase in estimated speciation rate indicated with a semi-transparent black circle, and the highest rate increase indicated with an asterisk (*). (For interpretation of the references to color in this figure legend, the reader is referred to the web version of this article)

Regarding the *corHMM*-based ACR for male terminalia traits, I found strong support for the MRCA of Miltogramminae and Paramacronychiinae having the posterior margin of the abdominal ST5 straight or with a shallow concavity, while it forms a cleft in the MRCA of Sarcophaginae (Additional file 4). Within the later, there is strong support for a transition from a posterior margin of the abdominal ST5 forming a cleft towards a straight or a shallow concavity shape in the MRCA of the clade containing *Cistudinomyia* Townsend, *Dexosarcophaga* Townsend, *Nephochaetopteryx* Townsend, *Oxysarcodexia*, *Oxyvinia*, *Sarcofahrtiopsis* Hall, and *Tricharaea* Thomson. Regarding the outline of dorsal surface of cercal prong in lateral view, the MRCA of the three subfamilies have a straight or almost straight outline (Additional file 5), while the MRCA of the clade E has the outline of dorsal surface of cercal prong in lateral view with a proximal hump. There is support for a transition from a straight or almost straight cercal outline to an outline with a subapical saddle-shaped concavity followed by a hump in the MRCA of the clade containing genera *Engelimyia*, *Lipoptilocnema*, *Peckia*, *Sarcophaga*, and *Villegasia* Dodge. Regarding the connection between basi- and distiphallus, there is strong support for the MRCA of Miltogramminae and Paramacronychiinae having a continuous connection, while this connection is non-continuous in the MRCA of Sarcophaginae and continuous in the MRCA of the clade C (Additional file 6). This connection between basi- and distiphallus is studied in more detail with the inclusion of the character shape of connection between basi- and distiphallus, for which the ACR recovers a very strong support for a serial transition between the presence of a distinct hinge between basi- and distiphallus, a partially sclerotized connection and a fully sclerotized connection. More specifically, strong support is obtained for the MRCA of the clade B having a distinct hinge between basi- and distiphallus, while the MRCA of genus *Microcerella* has a partially sclerotized connection and the MRCA of the clade C has a fully sclerotized connection (Fig. 5). The ACR for the harpes gives numerous independent origins for these structures within Sarcophaginae (Additional file 7), while the ACR for the vesica provides a strong support for a single origin of this structure in the MRCA of Sarcophaginae, and a reversal to absence of vesica in the MRCA of genus *Blaesoxipha* (Additional file 8). Similarly, a folded phallotrema, forming three openings, seems to have evolved only once in the MRCA of Sarcophagidae (Additional file 9). There is support for a phallotrema placed in a ventral position with regard to the phallic tube in the MRCA of all sarcophagids, with the independent evolution of an apical phallotrema in the MRCA of Miltogramminae (Additional file 10). The ACR for the acrophallic levers supports only one origin in the MRCA of the clade C (Additional file 11). The number of styli of the phallus is estimated to be one in the MRCA of (Miltogramminae + Paramacronychiinae) while three in the MRCA of Sarcophaginae, with a reduction to two styli in the MRCA of the large clade F (Fig. 6a). There is support for a single origin of the capitis in the MRCA of Sarcophagidae, with two independent reductions in the MRCA of Miltogramminae and in four subgenera of the genus *Peckia* (Additional file 12). Regarding the evolution of the median process, the ARC provides support for a single reduction of this structure in the MRCA of the large clade F (Additional file 13). Finally, the juxta is estimated to have evolved only once in the ancestor of all sarcophagids and have reduced only once in the MRCA of the subfamily Miltogramminae (Additional file 14).

### Genus-level diversification of Sarcophagidae

**Fig. 7:**
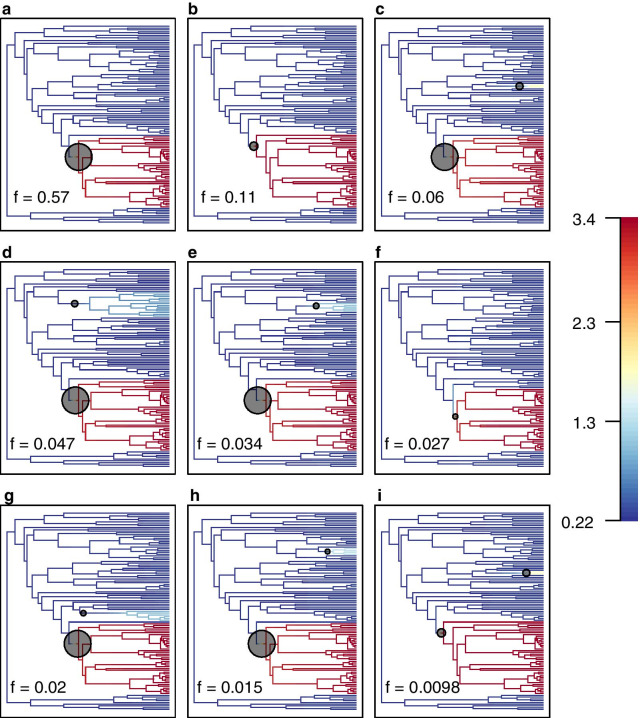
Diversification of Sarcophagidae and best shift configurations. BAMM analyses on the ASTRAL-III species tree estimated from the dataset having 2018 UCE loci coded as nucleotides (topology A) using *makeChronosCalib* in the R package *ape* and using clade-specific sampling probabilities (Additional file 15) to account for incomplete sampling based on species estimates. A–I. Top nine best shift configurations. (For interpretation of the references to color in this figure legend, the reader is referred to the web version of this article)

Rates of diversification were estimated in Sarcophagidae to investigate whether significant rate shifts have occurred over time, using (1) the time calibrated topology from the coalescent-based tree reconstructed from the dataset containing 2018 UCE loci and coded as nucleotides and (2) a set of sampling fractions based on species estimates (Additional file 15). I plotted the mean marginal posterior density of speciation rates in Fig. 6b. This phylorate graph shows an increase in estimated rate along the branch leading to genus *Sarcophaga* (semi-transparent black circle in Fig. 6b) excluding subgenus *Neobellieria* (light blue branch in Fig. 6b). The highest rate increase occurs along the branch leading to a clade containing several subgenera of *Sarcophaga* (indicated with an asterisk on Fig. 6b). All of the top nine most credible rate shift configurations support an increase in estimated rate along the branch leading to or within the genus *Sarcophaga* (Fig. 7). Some of the top nine most credible rate shift configurations support additional rate increases in other branches but with very low frequency (= posterior probability support) (f = 0.06–0.0098) (Fig. 7c–e, g–i). Moreover, the frequency for the best shift configuration was high (f = 0.57), and the alternative eight best shift configurations were generally consistent with this (Fig. 7).

## Discussion

The family Sarcophagidae is a highly diverse group, being one of the largest insect radiations among the living organisms [1]. The present study corroborates previous findings regarding the monophyly of sarcophagids [18, 23–25, 33, 39–43] and its three subfamilies [6, 9, 10, 18, 25, 33]. Several studies have challenged the traditional classification and questioned subfamily-level relationships [25, 40, 44], but more evidence is accumulating in support of Paramacronychiinae as sister to Miltogramminae [8, 9, 18, 23, 33]. Regarding the phylogenetic relationships within Miltogramminae and Paramacronychiinae, the relationships recovered here are consistent with those estimated using other molecular datasets. For example, *Eumacronychia* Townsend is sister to the remaining Miltogramminae [18] and the genera *Dexagria* Rohdendorf, *Paramacronychia* Brauer & Bergenstamm, and *Brachicoma* Rondani are early-branching lineages within Paramacronychiinae [9].

The relationships within the subfamily Sarcophaginae have historically been a challenge for phylogeneticists. Previous morphology-based studies suggested phylogenetic hypotheses for the genera of this subfamily [7, 45], which are not fully supported by Sanger-based phylogenies [22, 23, 25]. Most of these studies have important differences in taxon sampling and molecular markers, and many of them received weak statistical support. More recent next generation sequencing (NGS)-based phylogenies using transcriptomes [8], anchored hybrid enrichment [9], and UCEs [33] received stronger statistical support, but had limited taxon sampling for genera. In the present study, the use of UCE data augmented by improved taxon sampling results in a much better resolved phylogeny, with most nodes receiving maximum support (Fig. 2).

The new phylogeny does not generally contradict well-supported clades in the previous NGS-based studies but does uncover many novel relationships that are revealed by the inclusion of a wide sampling of genera. These novel relationships include (1) the placement of the clade A or (*Lepidodexia* (*Emdenimyia* (*Boettcheria* + *Tripanurga*))) as sister to the remaining sarcophagids, (2) the sister-group relationship between *Microcerella* and the clade C (Fig. 2), (3) the sister-group relationship between *Cistudinomyia* and *Tricharaea*, (4) the sister-group relationship between *Dexosarcophaga* and (*Nephochaetopteryx* + *Sarcofahrtiopsis*), (5) the sister-group relationship between *Argoravinia* Townsend and (*Malacophagomyia* Lopes (*Titanogrypa* + *Rafaelia* Townsend + *Tulaeopoda* Townsend)) in clade D, (6) the sister-group relationship between (*Comasarcophaga* (*Mecynocorpus* + *Fletcherimyia*)) and (*Emblemasoma* Aldrich (*Spirobolomyia* Townsend + *Blaesoxipha*)), (7) the sister-group relationship between *Helicobia* and a large clade containing genera *Chrysagria*, *Peckiamyia*, *Retrocitomyia*, *Villegasia*, *Peckia*, *Engelimyia*, *Lipoptilocnema*, and *Sarcophaga*, and (8) the sister-group relationship between (*Peckia* (*Pattonella*) Enderlein (*Peckia* (remaining subgenera) (*Engelimyia* + *Lipoptilocnema*))) and *Sarcophaga*. These results partially agree with phylogenetic relationships for Sarcophaginae based on morphological and molecular characters. For example, the morphology-based ‘Blaesoxipha clade’ of Buenaventura & Pape [7] is represented here by *Blaesoxipha*, *Comasarcophaga*, *Fletcherimyia*, *Mecynocorpus*, and *Spirobolomyia* and is recovered as a well-supported clade, although the clade recovered here also includes *Emblemasoma*. The internal relationships of this clade support those previously recovered by morphology [45] and UCEs [33] with *Blaesoxipha* as sister to *Spirobolomyia* and *Comasarcophaga* closely related to *Fletcherimyia*. The results partially support the morphology-based ‘Sarcophaga clade’ of Buenaventura & Pape [7], which includes genera *Chrysagria*, *Engelimyia*, *Helicobia*, *Lipoptilocnema*, *Peckia*, and *Sarcophaga*, although the clade recovered here also includes *Peckiamyia*, *Retrocitomyia*, and *Villegasia*. The present study also supports the existence of clades that previously had only weak or ambiguous support such as a *Boettcheria* + *Tripanurga* [22] and *Peckiamyia* + *Retrocitomyia* [7, 46].

Within the largest radiation of Sarcophaginae, phylogenetic relationships in the hyperdiverse genus *Sarcophaga* closely match those recovered in previous molecular phylogenies, with the Nearctic subgenus *Neobellieria* as sister-group to the remaining *Sarcophaga* species [10, 11, 33]. An early-branching clade including subgenera *Heteronychia* Brauer & Bergenstamm and *Brasia* Strand is also supported by data from an anchored hybrid enrichment analysis [9]. The clade (*Sarcophaga* + *Thyrsocnema*) has also been supported in previous molecular phylogenies [9–11, 33], as well as its close relationship to *Myorhina* [9, 11]. For the first time the Nearctic endemic subgenus *Neosarcophaga* is included in a phylogenetic study and all analyses recovered it in clade H as the sister to the Palaearctic clade (*Myorhina* (*Sarcophaga* + *Thyrsocnema*)). The relationships within clade J with subgenera *Bellieriomima*, *Helicophagella*, *Mauritiella* Verves, and *Asceloctella* Enderlein (among others) and those of clade K with subgenera *Pandelleana* Rohdendorf and *Sarcorohdendorfia* Baranov (among others) highly resemble those recovered in previous NGS-based phylogenies [9, 33]. Within clade K, the sister-group relationship between *Stackelbergeola* Rohdendorf and *Rohdendorfisca* Grunin is also consistent with previous Sanger- and NGS-based phylogenies [9, 33, 47]. A close relationship between *Bercaea* and *Liopygia* has been suggested in previous phylogenies [9], which is supported here with *Liopygia* rendered paraphyletic by *Bercaea*. Similarly, a close relationship between subgenera *Liosarcophaga*, *Parasarcophaga*, *Robineauella*, and *Rosellea* is supported by a previous molecular analysis [11] and it is here confirmed with clade M that has (*Robineauella* (*Parasarcophaga* (*Liosarcophaga* + *Rosellea*))). Most of the similar phylogenetic results of the present study and those of previous NGS-based phylogenies are due to taxon sampling compatibility and not to data duplication or data similarity, as the loci analyzed in these studies are not compatible but they give a significantly consistent phylogenetic signal.

### Evolution of life habits

Flesh flies successfully feed on a breadth of live or dead hosts (vertebrates, invertebrates) and substrates (feces), yet these vary tremendously in accessibility, volatile profile, attractiveness to potential competitors, as well as the set of behavioral, chemical, and morphological specializations needed for the gravid females and the first instar larvae to survive interactions with their hosts. A significant distinction can usually be made between a community of predatory species and a community of decomposer species living as sarcosaprophages, coprophages, or kleptoparasitic flies. There is a general fidelity in the choice of larval feeding habits, with some flesh-fly genera adapted to behave as predators on invertebrates (*Blaesoxipha*, *Boettcheria*, *Chrysagria*, *Cistudinomyia*, *Emblemasoma*, *Emdenimyia*, *Lepidodexia*, *Malacophagomyia*, *Rafaelia*, *Spirobolomyia*, *Tripanurga*, and several subgenera of *Sarcophaga*) and a group of genera adapted as decomposers of organic matter (sarcosaprograges and coprophages) either on invertebrates or vertebrates (*Engelimyia*, *Lipoptilocnema*, *Microcerella*, *Nephochaetopteryx*, *Peckia*, *Peckiamyia*, *Oxysarcodexia*, *Oxyvinia*, *Ravinia*, *Retrocitomyia*, *Sarcofahrtiopsis*, *Tricharaea*, and *Villegasia*). Very few Sarcophagidae taxa can be considered generalists, as usually only one or two species in particular genera are able to feed on different trophic substrates according to their availability. However, it has historically been unclear how this manifold ecology of predators, sarcosaprograges, coprophages, and kleptoparasites evolved within the flesh flies.

The evolutionary patterns giving origin to the diverse ecology of sarcophagids and hosts to which these flies have been associated in their evolution have not been fully addressed. The lack of a well-supported phylogeny of Sarcophagidae and its sister family (Oestridae) has been the most limiting factor. However, the scarcity of information on the biology of the group has also been a relevant limitation. The possibility of having a robust phylogeny for Sarcophagidae has improved over the years with the advance of phylogenetic methods and the efficiency in NGS-based molecular techniques and morphological methods for accessing and collecting different sources of data for phylogenetic reconstruction. The knowledge on the biology of the group, especially of some genera, continues to be a limitation, which can be mitigated with the use of algorithms for ancestral character state reconstruction that allow for missing data. However, this comes with costs in the uncertainty of the estimations.

Earlier hypotheses suggested that at least the subfamily Sarcophaginae had a coprophage or saprophage ancestor, and was undergoing the change from coprophagous and saprophagous nutrition to pure parasitic nutrition [48]. Regarding subfamily Miltogramminae, a recent hypothesis suggests that larvae of ancient miltogrammines were sarcosaprophagous [18]. An ancestral sarcosaprophagous Sarcophagidae fly feeding on invertebrates as primary food source was recently supported by the first formal analysis of this question, and the ancestors of the subfamilies Miltogramminae and Paramacronychiinae were further reconstructed as sarcosaprophagous feeding on both vertebrates and invertebrates [8]. These hypotheses are only partially supported by the data presented here. The results presented here support kleptoparasitism (or inquilinism) of Miltogramminae arising from a predator (i.e., parasitic) ancestor [49, 50]. In contrast to previous hypotheses, the MRCA of all sarcophagids is here estimated to have been a predator on invertebrates, which is consistently supported by both the Mesquite-based (including the sister-family Oestridae, among other outgroups) and the *corHMM*-based (including only Sarcophagidae) ancestral character state reconstructions. This same combination of larval feeding habit and larval food resource is also supported for the MRCA of each of the three subfamilies.

Differences in conclusions between previous hypotheses and the present analyses are probably due to (a) differences in taxon sampling and (b) differences in the topologies used for ACR. The MRCA of Miltogramminae subfamily is estimated as sarcosaprophagous when species of only this subfamily are analyzed (e.g., [18]). However, when the scope of the analysis includes other subfamilies and outgroups of other Oestroidea families, such as Oestridae, i.e., the sister-group of Sarcophagidae, the MRCA of Miltogramminae is reconstructed as a predator on invertebrates. Differences in topologies, especially regarding the earliest divergences, have an important impact on the ACR. For example, the inclusion of the genus *Galopagomyia* Bischof as sister to the remaining Paramacronychiinae in the supertree of Yan et al. [8] dramatically affects the estimated ancestral life history for the subfamilies Miltogramminae and Paramacronychiinae, giving sarcosaprophagy on invertebrates or vertebrates as the ancestral character state of the MRCA of these subfamilies. The phylogenetic position of *Galopagomyia* has only once been evaluated in a phylogenetic analysis including only Paramacronychiinae species and two Sarcophaginae species as outgroup [44]. In that analysis, the sister-group relationship between *Galopagomyia* and the remaining Paramacronychiinae is supported by two character states, i.e., the color of the tegula with respect to the basicosta and the shape of the posterior margin of the ST5 [44], both of which can vary greatly across taxa of the family and do not constitute synapomorphies for the clade *Galopagomyia* + remaining Paramacronychiinae. For example, a black tegula contrasting with yellowish or light brown basicosta as observed in *Galopagomyia* is also found in many non-related genera of Sarcophaginae [7]. Similarly, many non-related genera of Sarcophaginae share with *Galopagomyia* a distinctly emarginated and either broadly U-shaped or distinctly V-shaped posterior margin of the ST5. The evaluation of these characters in the broad context of the family would most likely not support the position of *Galopagomyia* as sister to the remaining Paramacronychiinae. Therefore, the phylogenetic position of *Galopagomyia* is considered uncertain. This taxon was manually added to the supertree used for ACR in Yan et al. [8], and therefore its phylogenetic position continues to be uncertain. Without *Galopagomyia*, the supertree used for ACR in Yan et al. [8] would have genus *Agria* Robineau-Desvoidy, a predator on invertebrates, as sister to the remaining Paramacronychiinae. Thus, as in Yan et al. [8] the MRCA of Miltogramminae is estimated as a predator or sarcosaprophage on vertebrates or invertebrates, then an ACR with an adjusted topology (without *Galopagomyia*) would most likely estimate the MRCA of both Miltogramminae and Paramacronychiinae as a predator on invertebrates, which would be consistent with results presented here. Another topology effect in the estimations of Yan et al. [8] is related to the taxa populating the early phylogenetic divergences within Sarcophaginae. The MRCA of Sarcophaginae is estimated as a sarcosaprophage on invertebrates in Yan et al.’s [8] supertree, as it has *Tricharaea* and *Sarcofahrtiopsis*, which are sarcosaprohages/coprophages and sarcosaprohages, respectively, as laddered sister-groups to the remaining Sarcophaginae. In contrast, the present ACR estimates the MRCA of Sarcophaginae as a predator on invertebrates based on an UCE-based phylogeny providing robust support for (*Lepidodexia* (*Emdenimyia* (*Boettcheria* + *Tripanurga*))) or clade A as sister to the remaining Sarcophaginae (Figs. 3 and 4). Not only the phylogenetic position of this clade of invertebrate predators determines the ACR estimation for the MRCA of Sarcophaginae, but the subsequent divergence of another taxon, *Udamopyga* Hall, which is coded as predator or sarcosaprophage on invertebrates.

An interesting transition from the predatory habits on invertebrates towards non-predatory habits in the early divergences of Sarcophaginae is observed within the clade B (Figs. 3 and 4). The MRCA of clade B is estimated as a predator on invertebrates, while the MRCA of *Microcerella* is estimated as a sarcosaprophage on vertebrates and the MRCA of its sister clade C is estimated as a coprophagous. Within clade C, other interesting results are related to the habitats occupied by closely related taxa and their use of available resources. Thus, the sister-group relationship between *Tricharaea* and *Cistudinomyia* tells the story of taxa living on beaches (although *Tricharaea* can also be found in other environments), which are adapted to predate on a specific host like turtles in the case of *Cistudinomyia*, and a more plastic taxon like *Tricharaea* using both dead invertebrates and vertebrates as well as feces to feed on. Another transition from predatory habits to sarcosaprophagy occurs in the MRCA of the clade containing genera *Engelimyia*, *Lipoptilocnema*, and *Peckia*, and such transition is also observed in a few species of genera *Blaesoxipha* and *Argoravinia*, and in the MRCA of (*Sarcosolomonia* Baranov + *Sarcorohdendorfia*) within the genus *Sarcophaga*. In contrast, there are no transitions from sarcosaprophagy or coprophagy to predatory habits, but clearly overall sarcosaprophagy and coprophagy have evolved in a nonrandom fashion in Sarcophaginae. No transitions from an exclusively sarcosaprophagous habit back to predation are supported (although exceptions could occur in *Peckia* and some uncertainty remains regarding *Helicobia*). This may indicate benefits of an adaptation to sarcosaprophagy that prevent the reversal to a predatory lifestyle. These results also indicate that the origin of sarcosaprophagy would have taken place in the context of an existing ecology of an ancestor with a predator-host established relationship. This would also support a hypothesis of a gradual transition from predation to sarcosaprophagy or coprophagy, with predator flesh flies gradually attacking not only healthy hosts, but also injured or weakened hosts, or even dead hosts. A transition from predation to sarcosaprophagy or coprophagy would imply less risk for a predator flesh fly, while the opposite could mean a sarcosaprophagous flesh fly attacking a healthy host that could eventually counterattack. More detailed species-level analyses estimating ancestral larval feeding habits within genera including ‘generalist species’ (in genera such as *Argoravinia*, *Peckia*, *Sarcophaga*) are necessary, which in turn could lead to slightly different conclusions at the genus level.

The observation of non-reversals from predation to sarcosaprophagy or coprophagy could also be used in future research using a different approach to model the parameter process (transitions between the different rate classes) in a hidden Markov model to assume that sarcosaprophagy and coprophagy are not lost once they evolve, which contrasts with the assumption for the models used here where all transitions among the specified number of rate classes are the same (ER) or all transition rates are allowed and are independently estimated (ARD). Such a different approach would allow the inference of a biologically relevant, but unmeasured ‘hidden’ character that could have influenced the evolution of the observed characters here.

### Evolution of male terminalia traits

Male terminalia traits evolved almost equally convergently and non-convergently in multiple lineages across all three main clades of Sarcophagidae, as it has been found in other studies [9], although there is a slight dominance of non-convergent traits here. The six convergent traits were the shape of posterior margin of the abdominal ST5, outline of dorsal surface of cercal prong, connection between basi- and distiphallus, shape of connection between basi- and distiphallus, harpes, and capitis, while the seven non-convergent traits were the vesica, phallotrema configuration, phallotrema position with regard to phallic tube, acrophallic levers, number of styli, median process, and juxta.

Regarding convergent traits, there are degrees in convergence with character states evolving twice in the phylogeny, while other characters have multiple independent origins across the tree. For example, a straight posterior margin of the abdominal ST5 evolves twice independently in the MRCA of (Miltogramminae + Paramacronychiinae) and in the MRCA of the clade containing genera *Cistudinomyia*, *Dexosarcophaga*, *Nephochaetopteryx*, *Oxysarcodexia*, *Oxyvinia*, *Sarcofahrtiopsis*, and *Tricharaea*. An absent or reduced capitis also evolves twice in subfamily Miltogramminae and some subgenera of genus *Peckia*. Whereas characters like the outline of dorsal surface of cercal prong and the harpes have multiple independent origins and few reversals across the sarcophagid tree. The multiple origins of the harpes and possibly also the loss of capitis could be explained by a homology definition problem, as at least the harpes are difficult to delimitate and could be confused with other accessory appendages of the phallus.

An interesting case of convergence is observed for the traits related to the connection and shape of connection between basi- and distiphallus. In general, a continuous connection between basi- and distiphallus evolves twice in the family, once in the MRCA of clade (Miltogramminae + Paramacronychiinae) and another time in the MRCA of clade C (Additional file 6). A second character, which looks into the details of this connection, shows that there is a transition in the degree of sclerotization originating the continuous connection between basi- and distiphallus in clade C (Fig. 5). Thus, the MRCA of clade B has a distinct hinge between basi- and distiphallus (Fig. 5a, b), while the MRCA of genus *Microcerella* has an intermediate character state between a distinct hinge and a fully sclerotized connection (Fig. 5c), and the MRCA of clade C has a fully sclerotized connection (Fig. 5d–f). The intermediate character state of the genus *Microcerella* consists of a hinge on the dorsal side of the phallus and a sclerotized, paler, rigid and tubular area on the ventral side of the phallus between basi- and distiphallus. This sclerotized, paler, rigid and tubular ventral area between basi- and distiphallus had been described before [51] but not analyzed in a broader phylogenetic context of the family. Such transitions in the degree of sclerotization in the morphological evolution of Sarcophagidae or for lineages within Miltogramminae or Paramacronychiinae have not been reported before.

The diversification of the subfamily Sarcophaginae, which includes 2/3 of the diversity of the family, is marked by the concerted evolution of a set of phallic traits that were found to be non-convergent. Some of these phallic traits involve complex structures like the juxta, vesica, and a complex acrophallus (with various styli), which have only isolated reversals or losses. The loss of complex structures as irreversible over time is a concept known as Dollo’s law [52]. Although this evolutionary principle is still commonly accepted, a number of cases where it is apparently violated have been proposed. Here I found that most of the complex phallic structures (e.g., juxta, vesica, a complex acrophallus with more than one styli) are rarely lost once they have evolved, and only the harpes seem to be the exception. The juxta originates most probably in the ancestor of all sarcophagids, and became a more complex structure separate by a hinge from the rest of the phallus in the clade A (*Lepidodexia* (*Emdenimyia* (*Boettcheria* + *Tripanurga*))) (Additional file 14), which constitutes the first branching within Sarcophaginae. The phallotrema placed in a ventral position with regard to phallic tube follows the same evolutionary pattern as the juxta (Additional file 10). The vesica and folding of the phallotrema evolve in the MRCA of Sarcophagidae (Additional files 8, 9), while the acrophallic levers evolve in the MRCA of clade C (Additional file 11). There might be some correlation between morphological characters, such as the concerted evolution of a phallotrema placed in a ventral position with regard to phallic tube and the origin of the juxta. The observed pattern in Miltogramminae suggests certain dependency between these characters, as species in this subfamily have a phallotrema placed in an apical position and absence of juxta. Similarly, concerted evolution is observed between the origin of the vesica and the folding of the phallotrema. Similar to the previous couple of characters, Miltogramminae lacks both vesica and the folding of the phallotrema. Interestingly, the vesica is particularly ornamented and complex in the genera *Cistudinomyia*, *Dexosarcophaga*, *Nephochaetopteryx*, *Oxysarcodexia*, *Oxyvinia*, *Sarcofahrtiopsis*, and *Tricharaea*, and it seems to be functionally related to an extrusion of the styli during mating, which is mediated by the acrophallic levers [7], another trait showing a non-convergent evolution that matches the evolutionary pattern of an ornamented vesica.

Regarding the relation between traits and diversification rate, the only trait having an evolutionary pattern close to the increased rate of diversification identified along the branch leading to genus *Sarcophaga* (Fig. 6b) is the number of styli (Fig. 6a). The MRCA of Sarcophaginae was estimated to have had three styli, which transitions to two styli in the MRCA of the large clade F that contains *Sarcophaga* and other genera (Fig. 2). There is no evident explanation on how the reduction in number of styli could have been related to the massive radiation within the genus *Sarcophaga*.

### Diversification of Sarcophagidae

The majority of the flesh fly diversity is represented by the subfamily Sarcophaginae, which contains three of the largest, most species-rich genera within the Sarcophagidae: *Blaesoxipha*, *Lepidodexia*, and *Sarcophaga*. In the present study, one diversification rate shift was inferred, which is associated unsurprisingly with the genus *Sarcophaga* that is the most species-rich genus and among the geographically most widespread taxa of flesh flies. The genera *Blaesoxipha* and *Lepidodexia* are not associated with any diversification rate shift. Incomplete taxon sampling could have influenced the age estimates for *Blaesoxipha* and *Lepidodexia* in the dating analyses. In addition, *Lepidodexia* is the least studied among the most species-rich genera, which could suffer from an underestimated number of species. The sensitivity of BAMM analyses to the selected rate shift prior in estimations of diversification rates and rate shifts (under certain circumstances) has been questioned [53, 54]. Similarly, a tendency to overestimate diversification rates in smaller clades, which may result in a potential underestimation of rate shifts overall, has also been identified and criticized [55]. The results presented here are most likely not biased by these sensitivity and overestimation issues affecting the diversification rate estimates, given that I recovered only one statistically significant shift for the most species-rich genus within Sacophagidae, which essentially confirms observations based on taxonomic species diversity. These results are concordant with studies showing lineages within Sarcophagidae [9, 10, 18, 23] as the dominant fast-evolving groups of Oestroidea. Furthermore, these results support a super-radiation within the genus *Sarcophaga*, as recent studies suggest [9–11, 23, 33].

The dynamics of evolutionary diversification are usually linked to ecological opportunity and the evolution of a key innovation. This means that increases in diversification rates could be the result of a lineage that evolved and diversified into previously inaccessible environmental niche space because of an ecological opportunity event (e.g., dispersal to and colonization of a new environment or extinction of a previously dominant group) or with the evolution of a key innovation, such as a novel trait (be it morphological, physiological, or genetic), resulting in rapid speciation as niche space is partitioned unencumbered by biological interactions, such as competition and predation [56]. Evidence for morphological diversification under increased diversification rates has been recovered in some clades of insects [57] and mammals [58, 59] using phylogenetic methods. Similarly, there is evidence for positive shifts in diversification rate of ants [57, 60, 61] and beetles [62] possibly associated with increased ecological opportunity in the form of biogeographic dispersal. In the case of the increased diversification rate inferred here for the genus *Sarcophaga*, this could be the result of an ecological opportunity event. Previous research showed this genus evolved in the Nearctic region and experienced a rapid radiation occurring in the Nearctic region with a subsequent dispersal into the Palaearctic region [10]. This is supported by the present results, although the diversification rate inferred here shows that the rate increase occurred once some *Sarcophaga* lineages had dispersed into the Palaearctic region and not before in the Nearctic region. Thus, the increased diversification rate inferred here for the large part of the genus *Sarcophaga* could be the result of a lineage that evolved and diversified into a previously inaccessible environmental niche space. Thus, the observed resemblance between the evolutionary pattern of traits such as the number of styli and pattern of diversification rate might not explain the rapid radiation of the non-Nearctic lineages of *Sarcophaga*. Even if the evolutionary pattern of this trait closely resemble the diversification pattern of *Sarcophaga*, the reduction in the number of styli is observed for the large clade F, which includes *Sarcophaga* but also many other genera (i.e., *Chrysagria*, *Helicobia*, *Peckiamyia*, *Retrocitomyia*, *Villegasia*, *Peckia*, *Engelimyia*, and *Lipoptilocnema*) that did not show increases in diversification rates and did not expand their biogeographic distribution beyond the Neotropical region. This hypothesis of diversification associated with biogeographic dispersal and ecological opportunity could be further tested with extended species-level phylogenetic, biogeographic, and biological data, while hypotheses regarding the evolutionary significance and influence of innovative features such as morphological traits in the diversification of sarcophagids will possibly present more difficult challenges.

## Conclusions

The phylogenomic approach combining taxon-specific, protein encoding, UCE probes with a large ingroup and outgroup sampling obtained a well-supported phylogeny for Sarcophagidae at the subfamily, genus, and species level. I was able to firmly place a group of predatory flies including the genera *Lepidodexia* and *Boettcheria* as sister-group to the rest of Sarcophaginae, which contrasts with previous phylogenetic hypotheses for sarcophagids. Similarly, genera *Oxysarcodexia*, *Ravinia*, and *Tricharaea*, long considered archaic and early-branching coprophagous and sarcosaprophagous lineages, were found nested well within the Sarcophaginae as sister-group to the sarcosaprophagous *Microcerella*. The ACR estimated predation on invertebrates as the ancestral and dominant strategy throughout the early evolution of flesh flies independent of the type of ancestral character reconstruction analysis performed (Mesquite- or *corHMM*-based). This strategy is also supported for the most recent common ancestor of each of the three subfamilies. These estimations also suggest that sarcosaprophagy, coprophagy, and kleptoparasitism evolved from predation. Thus, several transitions from predation to sarcosaprophagy and coprophagy were estimated across the phylogenetic tree of sarcophagids, in contrast with almost no transitions from sarcosaprophagy or coprophagy to predatory habits. The evolution of morphological traits seems not related with the change in feeding habit, although there might be a correlation between morphological characters only, such as the concerted evolution of the position of the phallotrema and the origin of the juxta, or the origin of the vesica and the folding of the phallotrema. The only diversification rate shift inferred in the evolution of Sarcophagidae is associated unsurprisingly with the richest and geographically most widespread taxon, the genus *Sarcophaga*, which could be the result of an increased ecological opportunity in the form of biogeographic dispersal and colonization of the Palaearctic region. As suggested in early studies on Sarcophaginae, a correlation could be present between feeding habits and physiological rather than morphological change [48], which is a hypothesis that should be tested in future research. Future studies using UCEs should include and phylogenetically place enigmatic taxa such as genera *Carinoclypeus* Dodge, *Galopagomyia*, *Sarcodexiopsis* Townsend, *Sarothromyiops* Townsend, *Sinopiella* Lopes & Tibana, *Tapacura* Tibana & Lopes, and *Thomazomyia* Lopes. By reconstructing a robust phylogeny and highlighting patterns of life histories and morphological evolution, this study has established the framework for further physiological and comparative genomic work between predatory, sarcosaprophage, coprophage, and kleptoparasitic lineages, which could also have significant implications for the evolution of diverse life histories in other Diptera.

## Methods

### Taxon sampling

Taxon sampling included 110 ingroup Sarcophagidae species of the subfamilies Miltogramminae (4 spp.), Paramacronychiinae (4 spp.), Sarcophaginae (102 spp.), and 31 outgroup species representing the families Anthomyiidae (2 spp.), Calliphoridae (7 spp.), Fanniidae (2 spp.), Mesembrinellidae (2 spp.), Oestridae (4 spp.), Polleniidae (1 sp.), Rhiniidae (4 spp.), Rhinophoridae (3 spp.), Scathophagidae (1 sp.), and Tachinidae (5 spp.). The selected outgroups represent fly families that could question the monophyly of Sarcophagidae and that are relevant representatives to evaluate the evolution of life history within the superfamily Oestroidea. Even tough the monophyly of Sarcophagidae has been studied and tested before [24, 25, 33], the present study includes a large outgroup in order to produce a robust phylogeny for the ancestral character state reconstruction. Thus, the present study includes the closest relatives of Sarcophagidae, i.e., fly families of the superfamily Oestroidea (Calliphoridae, Mesembrinellidae, Oestridae, Polleniidae, Rhiniidae, Rhinophoridae, and Tachinidae, except Mystacinobiidae and Ulurumyiidae) and more distant relatives belonging to the Muscoidea grade (i.e., Anthomyiidae, Fanniidae, Scathophagidae). All specimens included in this study were collected in accordance with local regulations and all necessary permits were obtained. Voucher specimens have been deposited at the Entomological Collection of Alicante University (CEUA), Museum für Naturkunde (MFN), Musée royal de l’Afrique centrale (RMCA), National Museum of Natural History (USNM), Natural History Museum of Denmark (NHMD), and Entomological Collection of Wright State University (JOSC) (see Additional file 1 for specimen identifiers and collection data).

### UCE data collection

Genomic DNA was obtained from DNA aliquots, pinned museum specimens, specimens preserved in 96% Ethanol, and in Liquid Nitrogen. I used 32 existing DNA aliquots from previous molecular studies [10, 11, 33, 47], which were stored in a − 20 °C freezer. Genomic DNA was extracted from 24 pinned specimens, 66 specimens preserved in 96% Ethanol, and 19 specimens collected and placed directly in empty vials stored in Liquid Nitrogen in the National Museum of Natural History (USNM) Biorepository, as indicated in Additional file 1, where specimen identity, preservation method, targeted tissue for extraction (i.e., thorax, abdomen, legs or the whole body excluding terminalia), collection data and corresponding repositories at natural history museums of all specimens is provided. Dust, pollen, and other forms of accumulated debris on pinned specimens were removed using sterilized forceps and a soft paintbrush. DNA was non-destructively extracted from the thorax of pinned specimens, while it was destructively extracted from specimens preserved in 96% ethanol and liquid nitrogen by grinding the tissue with a sterile pestle. DNA extractions used a DNeasy Blood and Tissue Kit (Qiagen, Valencia, CA, USA) and followed the manufacturer’s protocol, but to maximize DNA yield the Proteinase K digestion ran for 48 h at 56 °C and DNA was eluted twice in 50 µL (total volume 100 µL). To estimate size of the genomic DNA, 10 µL of each extract were run for 40 min at 100 volts on 1.5% agarose SB (sodium borate) gels.

### Library preparation, target enrichment, and sequencing of UCEs

Extracted genomic DNA was quantified using a Qubit fluorometer (High sensitivity kit, Life Technologies, Inc.). DNA (0.3–590 ng, 107.1 ng mean) was sheared to a target size of approximately 500–600 bp by sonication (Q800, Qsonica LLC.), depending on prior degradation and fragmentation of DNA. This sheared DNA was used as input for library preparation following a protocol for UCEs by Faircloth et al. [63] and detailed in Blaimer et al. [34]. For adapter ligation, I used Tru-Seq-style adapters [64] and PCR amplified 50% of the resulting library volume (15 µL) with a reaction mix of 25 µL HiFi HotStart polymerase (Kapa Biosystems), 2.5 µL each of Illumina TruSeq-style i5 and i7 primers (5 µM each), and 5 µL double-distilled water (ddH20). I used the following thermal protocol: 98 °C for 45 s; 13 cycles of 98 °C for 15 s, 65 °C for 30 s, 72 °C for 60 s, and final extension at 72 °C for 5 m. After rehydrating (in 23 µL pH 8 Elution Buffer) and purifying reactions using 1.0× speedbeads, 8 libraries were combined at equimolar ratios into enrichment pools with final concentrations of 85.4–189 ng/µL. Each pool was enriched using a set of 5,117 custom-designed probes targeting 2,581 UCE loci in Calyptratae flies [33] using the MYcroarray MYBaits kit [65], except I used a 0.1x concentration of the standard MYBaits concentration, and added 0.7 µL of 500 µL custom blocking oligos designed against the custom sequence tags. The pool hybridization reaction ran for 24 h at 65 °C. Subsequently, I bound all pools to streptavidin beads (MyOne C1, Life Technologies) and washed bound libraries according to a standard target enrichment protocol [65]. Post-enrichment amplification was performed on beads with the KAPA Hifi HotStart ReadyMix using the following thermal profile: 98 °C for 45 s; 18 cycles of 98 °C for 15 s, 60 °C for 30 s, 72 °C for 60 s; and a final extension of 72 °C for 5 m. Post-enrichment libraries were purified using 1.0x speedbeads (Sera-mag, GE Healthcare) and rehydrated the enriched pools in 22 µL TLE. Post-enrichment library concentration was quantified via qPCR using a SYBR® FAST qPCR kit (Kapa Biosystems) on a ViiA™ 7 (Life Technologies). Based on the size-adjusted concentrations estimated by qPCR, I pooled libraries at equimolar concentrations and size-selected for 250–800 with a BluePippin (SageScience) (1.5% agarose, 250 bp–1.5 kb), and the pool-of-pools was quality checked on an Agilent 2200 TapeStation. The pooled libraries were sequenced using two lanes of a 125-bp paired-end Illumina HiSeq 2500 run (University of Utah Genomics Core Facility).

### Processing and alignment of UCE data

Illumiprocessor [66], based on the package Trimmomatic [67], was used to trim the demultiplexed FASTQ data output for adapter contamination and low-quality bases. Cleaned reads were assembled using Trinity [68]. All further data processing relied on the PHYLUCE package [69, 70] with Python scripts designed by the Smithsonian Institution Bioinformatics Group (available at www.github.com/SmithsonianWorkshops/Targeted_Enrichment/blob/master/phyluce.md). Summary statistics were computed on the data using the *phyluce_assembly_get_fastq_stats.py* script. Average sequencing coverage and contig length across assembled contigs were calculated using the *phyluce_assembly_get_trinity_coverage.py* script. To identify contigs representing enriched UCE loci from each species, species-specific contig assemblies were aligned to a FASTA file of all enrichment baits (min_coverage = 70, min_identity = 80), and sequence coverage statistics (avg, min, max) for contigs containing UCE loci were calculated. I created FASTA files for each UCE locus containing sequence data for taxa present at that particular locus and aligned these using MAFFT [71] (min-length = 20, no-trim). Alignments were trimmed using Gblocks [72] with relaxed settings (b1 = 0.5, b2 = 0.5, b3 = 12, b4 = 7). The alignment design used two different configurations. First, in an exploratory stage, concatenated UCE alignments having varying loci occupancy (0.1–0.9, nine datasets) were submitted to phylogenetic reconstruction and analyzed as nucleotides. Second, UCE alignments having varying loci occupancy (0.1, 0.3, 0.6, 0.75, four datasets) were submitted to phylogenetic reconstruction and analyzed both as nucleotides and amino acids. All of these datasets (Table 1) were designed to evaluate the relative contribution of varying amounts of UCE loci as nucleotides or amino acids to the construction of the phylogenetic tree.

### Phylogenomic analyses

First, datasets having varying loci occupancy and coded as nucleotides (analyses 1–9 in Table 1) were analyzed as concatenated datasets under Maximum Likelihood (ML). ML best tree (model GTRGAMMAI) and bootstrap searches (N = 100) of the nine concatenated datasets were conducted in RAxML v8.2.7 [73]. As the parameters Gamma and the proportion of invariable sites cannot be optimized independently from each other, I also analyzed the nine concatenated datasets using the GTRCAT model, which showed a reduction in conflict between resulting topologies, therefore these results are preferred. Subsequently, each of the eight datasets (analyses 10–17 in Table 1) having varying loci occupancy and coded as nucleotides and amino acids was analyzed using gene trees in a multi-coalescent species tree analysis with ASTRAL-III [74]. Data were partitioned by individual UCE loci using the Sliding-Window Site Characteristics approach and site characteristics such as entropy implemented in the SWSC-EN algorithm, which generates partitions that account for heterogeneity in rates and patterns of molecular evolution within each UCE [75]. A partitioning scheme from the by-locus character sets was selected with PartitionFinder2 [76]. Then, I sequentially ran an ML analysis for the best tree and 1000 replicates of ultrafast bootstrap on each locus for the gene tree estimations using IQ-TREE [77]. A multi-coalescent species tree analysis was carried out in ASTRAL-III using gene trees (one tree search per gene) estimated by 100 ML searches conducted in RAxML. Statistical supports by ASTRAL-III are local posterior probabilities (LPP), which are branch support values that measure the support for a quadripartition, not a bipartition.

All of the above phylogenomic analyses were performed on the Smithsonian Institution High Performance Cluster Hydra (SI/HPC) using Python scripts (designed by the Smithsonian Institution Bioinformatics Group, Michael Lloyd, and some modified by Bonnie Blaimer and myself) (available at www.github.com/SmithsonianWorkshops/Targeted_Enrichment/blob/master/phyluce.md). Tips of final trees were renamed using a Perl script (designed by Michael Lloyd and available at www.github.com/MikeWLloyd/Tree-Tip-Replacer).

### Dating analysis

Diversification rates of Sarcophagidae were estimated using the coalescent species-tree obtained from the dataset having loci occupancy 0.10 (includes 2018 UCE loci) and analyzed as nucleotides, which produced a strongly supported topology consistent with most of trees obtained from the remaining analyses. All of the outgroups were trimmed using the R package *phytools* v0.7-70 [78]. The coalescent species tree was transformed into a chronogram using correlated rates with *makeChronosCalib* in the R package *ape* v5.4-1 [79], setting the age of the most recent common ancestor of Sarcophagidae at 23 Ma (11.4–38.0) [2]. This age is uncertain due to the lack of fossils for sarcophagids and other groups of Oestroidea, but it is conservative with respect to other age estimates.

### Diversification rate estimation

An evaluation on whether shifts in diversification rates occurred over time in the evolution of Sarcophagidae was conducted using BAMM v2.5 [80, 81] and the associated R package *BAMMtools* v2.1.5 [81]. Incomplete sampling was accounted by using clade-specific sampling probabilities. To calculate sampling probabilities, a species-richness matrix was assembled. This matrix lists each tip (i.e., a species) of the phylogeny, which is assigned to a clade (i.e., its respective genus). Thus, the sampling fraction for each clade is calculated as the number of sampled species divided by number of described species in that clade (Additional file 15). The chronogram obtained above plus the set of sampling fractions were used to perform BAMM analyses, which followed the guidelines in the BAMM documentation (http://bamm-project.org/). The function *setBAMMpriors* within *BAMMtools* was used to obtain appropriate priors for speciation-extinction analyses, and the expected number of shifts was left at the default value (= 1). The Bayesian analysis included four MCMC chains with a length of 25 million generations, sampling every 10,000 generations, with discarded burn-in of 10%. Convergence was visualized using plots of log likelihoods of all sampled generations and all model parameters over time were examined focusing especially on effective sample size (ESS) values with the R package *coda* v0.19–4 [82]. Diversification rates were analyzed and visualized with various functions in *BAMMtools*, as follows: *computeBayesFactors* compared the evidence for models with at least one diversification shift to the evidence for the null model (zero diversification shifts) and identified the best-supported model of rate shifts; *credibleShiftSet* computed 95% credible set of distinct shift configurations that account for 95% of the probability of the data; *getBestShiftConfiguration* extracted the rate shift configuration with the maximum posterior probability (MAP); *plot.bammdata* plotted a ‘phylorate’ graph showing mean, model-averaged diversification rates along branches of the phylogeny.

### *Evolution of sarcophagid life histories and male terminalia traits*

Since I was interested in the evolutionary trajectory of life histories and male terminalia in sarcophagids, I inferred ancestral character states using the dated phylogeny. Biological data were compiled mostly from the literature [3, 4, 51, 172–176, 83–91, 5, 92–101, 6, 102–111, 13, 112–121, 14, 122–131, 18, 132–141, 20, 142–151, 47, 152–161, 49, 162–171] and personal collecting experience. Definition of life-history characters followed Yan et al. [8], where the characters larval food resource and larval feeding habits were selected to represent the breeding and feeding strategies of sarcophagid and Calyptratae larvae. The character larval food resource had five character states as follows: 0) invertebrates, (1) vertebrates, (2) feces, (3) plants, and (4) unknown. In the present study, the character larval feeding habits had six character states as follows: 0) kleptoparasitism, (1) sarcosaprophagy, (2) predation, (3) coprophagy, (4) phytophagy, (5) unknown. Yan et al. [8] did not include phytophagy as a character state of larval feeding habits. Following Yan et al. [8], predation was defined to include parasitism and parasitoidism; sarcosaprophagy (feeding on carrion) and coprophagy (feeding on faeces) were defined as distinct character states because their associated volatile profiles are different [177]; and kleptoparasitism was defined as a distinct character state due to the combined behavioural, chemical, and morphological especializations needed for the gravid females and the first instar larvae to survive interactions with their hosts [178].

Thirteen morphological characters of the male terminalia were selected from the dataset of Buenaventura & Pape [7]. The selected characters are: (1) Shape of posterior margin of the abdominal sternite 5 (ST5), (2) outline of dorsal surface of cercal prong in lateral view, (3) connection between basi- and distiphallus, (4) shape of connection between basi- and distiphallus, (5) harpes, (6) presence of vesica, (7) phallotrema configuration, (8) phallotrema position with regard to phallic tube, (9) presence of acrophallic levers, (10) number of styli, (11) presence of capitis, (12) median process, and (13) presence of juxta.

Using the biological and morphological characters described above, two datasets were produced. The first dataset includes all taxa sampled (ingroup and outgroup) for which only biological characters are scored (Additional file 16). The second dataset includes only ingroup species for which all of the 15 biological and morphological characters are scored (Additional file 17). The character state phytophagy of larval feeding habits was only included in the first dataset as this character state was sampled in the outgroup species and it is excluded from the second dataset as it was not sampled in the ingroup species. Morphological characters of the male terminalia were scored for the ingroup only and included only in the second dataset, as the homology of these characters needs to be further studied in comparative analyses across the whole Calyptratae clade. Species of flesh flies generally have marked preferences for carrion or faeces, but for some species, there is no certainty about their preferences. Adults of some species have been observed as visitors of both carrion and feces, but there is no information about their larval feeding strategies. In such cases, species were scored as polymorphic when feeding data supporting the polymorphism was available. When data was not available (unknown), species were scored with ‘?’ for the first dataset and with ‘n/a’ for the second dataset.

For ACR, I used the trace character option with Maximum parsimony under unordered states assumption in the software Mesquite version 3.04 (build 725) [179, 180] to trace the life-history characters both in outgroup and ingroup taxa using the first dataset. In parallel, also for ACR, I used the *rayDISC* function in the package *corHMM* in R v4.0.0 (https://www.R-project.org/) which can analyze multivariate traits and the time-calibrated phylogeny estimated while employing the median range calibration on the root node. The *corHMM*-based ACR was performed on each biological and morphological character independently using the second dataset. Thus, each ACR analysis used two models available in *corHMM*, ‘equal rates’ (ER) and ‘all rates different’ (ARD). All analyses were carried out twice with the same settings to ascertain robustness of reconstructions. The fit of these models (i.e., ER and ARD) for the ACR analysis on each character was compared using the resulting lnL scores and corrected Akaike information criterion (AICc, test corrected for small sample sizes) values (Additional file 3). Results are presented for ACR with models significantly better considering AICc estimates. When either ER or ARD are not significantly better, then results are presented for the model with the lowest AICc estimate.

## Supplementary Information

**Additional file 1.** Specimen data. Specimen identity, preservation method, targeted tissue for extraction, collection data, specimen identifiers and repositories at Natural History Museums, DNA input data, contig data, UCE capture data. Abbreviations: CEUA, Entomological Collection Alicante University; JOSC, Entomological Collection Wright State University; MFN, Museum für Naturkunde Berlin; NHMD, Natural History Museum of Denmark; RMCA, Musée royal de l'Afrique centrale; USNM, National Museum of Natural History.

**Additional file 2.** Phylogenetic relationships for Sarcophagidae and 10 other Calyptratae fly families inferred from 17 datasets having varying loci occupancy and coded as nucleotides and amino acids using a concatenated ML approach as well as by reconstructing a species tree estimated from UCE gene trees. Datasets are described in Table 1.

**Additional file 3.** Comparison of models used for ancestral character state reconstructions. Models compared are ‘equal rates’ (ER, one transition rate) and ‘all rates different’ (ARD). Significantly better values are in bold. Characters analysed are: (1) larval food resource, (2) larval feeding habits, (3) abdominal ST5, shape of posterior margin, (4) cercal prong, outline of dorsal surface in lateral view, (5) phallus, connection between basi- and distiphallus, (6) phallus, connection between basi- and distiphallus, shape, (7) harpes, (8) vesica, (9) phallotrema, configuration, (10) phallotrema, position with regard to phallic tube, (11) acrophallic levers, (12) styli, number, (13) capitis, (14) median process, and (15) juxta.

**Additional file 4.** Evolution of shape of posterior margin of the abdominal sternite 5 in Sarcophagidae. Ancestral character state reconstruction for shape of posterior margin of the abdominal sternite 5 using maximum likelihood and the *rayDISC* function in the R package *corHMM*. Only reconstruction of the best fitting model (ER) is shown. Pie proportions represent state probabilities estimated for each internal node. Character states are indicated in insets at the bottom.

**Additional file 5.** Evolution of shape of posterior margin of the outline of dorsal surface of cercal prong in lateral view. Ancestral character state reconstruction for shape of posterior margin of the abdominal sternite 5 using maximum likelihood and the *rayDISC* function in the R package *corHMM*. Only reconstruction of the best fitting model (ARD) is shown. Pie proportions represent state probabilities estimated for each internal node. Character states are indicated in insets at the bottom.

**Additional file 6.** Evolution of the connection between basi- and distiphallus in Sarcophagidae. Ancestral character state reconstruction for connection between basi- and distiphallus using maximum likelihood and the *rayDISC* function in the R package *corHMM*. Only reconstruction of the best fitting model (ER) is shown. Pie proportions represent state probabilities estimated for each internal node. Character states are indicated in insets at the bottom.

**Additional file 7.** Evolution of the harpes in Sarcophagidae. Ancestral character state reconstruction for harpes using maximum likelihood and the *rayDISC* function in the R package *corHMM*. Only reconstruction of the best fitting model (ARD) is shown. Pie proportions represent state probabilities estimated for each internal node. Character states are indicated in insets at the bottom.

**Additional file 8.** Evolution of the vesica in Sarcophagidae. Ancestral character state reconstruction for vesica using maximum likelihood and the *rayDISC* function in the R package *corHMM*. Only reconstruction of the best fitting model (ER) is shown. Pie proportions represent state probabilities estimated for each internal node. Character states are indicated in insets at the bottom.

**Additional file 9.** Evolution of the configuration of the phallotrema in Sarcophagidae. Ancestral character state reconstruction for configuration of the phallotrema using maximum likelihood and the *rayDISC* function in the R package *corHMM*. Only reconstruction of the best fitting model (ER) is shown. Pie proportions represent state probabilities estimated for each internal node. Character states are indicated in insets at the bottom.

**Additional file 10.** Evolution of the phallotrema position with regard to phallic tube in Sarcophagidae. Ancestral character state reconstruction for phallotrema position with regard to phallic tube using maximum likelihood and the *rayDISC* function in the R package *corHMM*. Only reconstruction of the best fitting model (ER) is shown. Pie proportions represent state probabilities estimated for each internal node. Character states are indicated in insets at the bottom.

**Additional file 11.** Evolution of the acrophallic levers in Sarcophagidae. Ancestral character state reconstruction for acrophallic levers using maximum likelihood and the *rayDISC* function in the R package *corHMM*. Only reconstruction of the best fitting model (ER) is shown. Pie proportions represent state probabilities estimated for each internal node. Character states are indicated in insets at the bottom.

**Additional file 12.** Evolution of the capitis in Sarcophagidae. Ancestral character state reconstruction for capitis using maximum likelihood and the *rayDISC* function in the R package *corHMM*. Only reconstruction of the best fitting model (ER) is shown. Pie proportions represent state probabilities estimated for each internal node. Character states are indicated in insets at the bottom.

**Additional file 13.** Evolution of the median process in Sarcophagidae. Ancestral character state reconstruction for median process using maximum likelihood and the *rayDISC* function in the R package *corHMM*. Only reconstruction of the best fitting model (ER) is shown. Pie proportions represent state probabilities estimated for each internal node. Character states are indicated in insets at the bottom.

**Additional file 14.** Evolution of the juxta in Sarcophagidae. Ancestral character state reconstruction for juxta using maximum likelihood and the *rayDISC* function in the R package *corHMM*. Only reconstruction of the best fitting model (ER) is shown. Pie proportions represent state probabilities estimated for each internal node. Character states are indicated in insets at the bottom.

**Additional file 15.** Species richness matrix used for diversification analyses. This table lists the assigned clade for each species, the number of species sampled, and the number of nominal species described. From these cladespecific values, sampling fractions were calculated and used for BAMM analyses.

**Additional file 16.** Dataset of biological characters including ingroup and outgroup. Characters: 1) Larval food resource: (0) invertebrates; (1) vertebrates; (2) feces; (3) plants; (4) ? (unknown); 2) larval feeding habits: (0) kleptoparasitism; (1) sarcosaprophagy; (2) predation; (3) coprophagy; (4) phytophagy; (5) ? (unknown).

**Additional file 17.** Dataset of biological and morphological characters including ingroup only. Characters: 1) Larval food resource: (0) invertebrates; (1) vertebrates; (2) feces; (3) n/a (unknown); 2) larval feeding habits: (0) kleptoparasitism; (1) sarcosaprophagy; (2) predation; (3) coprophagy; (4) n/a (unknown); 3) abdominal ST5, shape of posterior margin: (0) straight or with a shallow concavity; (1) forming a cleft; 4) cercal prong, outline of dorsal surface in lateral view: (0) straight or almost straight; (1) swollen or curved uniformly; (2) with a proximal hump; (3) with a subapical saddle-shaped concavity followed by a hump; 5) phallus, connection between basi- and distiphallus: (0) continuous; (1) non-continuous; 6) phallus, connection between basi- and distiphallus, shape: (0) desclerotized band; (1) distinct hinge; (2) partially sclerotized; (3) fully sclerotized; 7) harpes: (0) absent; (1) present; 8) vesica: (0) absent; (1) present; 9) phallotrema, configuration: (0) not folded, forming one opening; (1) folded, forming three openings; 10) phallotrema, position with regard to phallic tube: (0) apical; (1) ventral; 11) acrophallic levers: (0) present; (1) absent; 12) styli, number: (0) one; (1) three; (2) two; 13) capitis: (0) present; (1) absent; 14) median process: (0) present; (1) absent or reduced; 15) juxta: (0) absent; (1) present.

## Data Availability

The UCE data supporting the conclusions of this article are available in the Museum für Naturkunde repository under accession 10.7479/t80n-ta02 [181]. The datasets (biological and morphological data) supporting the conclusions of this article are included within the article and its additional files.

## Abbreviations

ACR: Ancestral character state reconstruction
AHE: Anchored hybrid enrichment
AICc: Corrected Akaike information criterion
ARD: All rates different
BS: Bootstrap support
bp: Base pairs
CEUA: Entomological Collection of Alicante University
ER: Equal rates
ESS: Effective sample size
JOSC: Entomological Collection of Wright State University
LPP: Local posterior probability
Ma: Million years ago
MAP: Maximum posterior probability
MCMC: Markov Chain Monte Carlo
ML: Maximum likelihood
MRCA: Most recent common ancestor
NGS: Next generation sequencing
NHMD: Natural History Museum of Denmark
NMNH: National Museum of Natural History
RMCA: Musée royal de l’Afrique central
SI/HPC: Smithsonian Institution High Performance Cluster Hydra
ST5: Abdominal sternite 5
SWSC-EN: Sliding-Window Site Characteristics Entropy
UCEs: Ultraconserved elements
USNM: United States National Museum of Natural History
MFN: Museum für Naturkunde
