# Supplementary material for: Museomics and phylogenomics with protein-encoding ultraconserved elements illuminate the evolution of life history and phallic morphology of flesh flies (Diptera: Sarcophagidae)
Source: BMC Ecol Evol. 2021 Apr 28;21:70. doi: 10.1186/s12862-021-01797-7 (PMC8082969; doi:10.1186/s12862-021-01797-7)
Supplement: Supplementary file 1 — Additional file 1. Specimen data. Specimen identity, preservation method, targeted tissue for extraction, collection data, specimen identifiers and repositories at Natural History Museums, DNA input data, contig data, UCE capture data. Abbreviations: CEUA, Entomological Collection Alicante University; JOSC, Entomological Collection Wright State University; MFN, Museum für Naturkunde Berlin; NHMD, Natural History Museum of Denmark; RMCA, Musée royal de l'Afrique centrale; USNM, National Museum of Natural History. [file 12862_2021_1797_MOESM1_ESM.pdf]

Additional file 1. Specimen data. Specimen identity, preservation method, targeted tissue for extraction, collection data, specimen identifiers and repositories at Natural History Museums, DNA input data, contig data, UCE capture data. Abbreviations: CEUA, Entomological Collection Alicante University; JOSC, Entomological Collection Wright State University; MFN, Museum für Naturkunde Berlin; NHMD, Natural History Museum of Denmark; RMCA, Musée royal de l'Afrique centrale; USNM, National Museum of Natural History.

Specimen identity							Sample type		Extraction	Contigs			UCE loci		
Family	Subfamily	Species	NHM	Specimen ID	Specimen age (years)	Country	Prep.	Tissue	DNA (ng/µL)	Total	Total (bp)	Mean length (bp)	Total	Total (bp)	Mean length (bp)
Anthomyiidae		*Leucophora maculata*	USNM	USNMENT01137798	2	USA	LiqNit	Thorax	92.6	1,578	464,638	294.4	694	216,450	311.9
Anthomyiidae		*Anthomyia procellaris*	USNM	USNMENT01137770	2	USA	LiqNit	Thorax	266	1,342	410,504	305.9	664	219,488	330.6
Fanniidae		*Fannia sp.*	USNM	USNMENT01410976	1	USA	LiqNit	Thorax	114	1,675	510,395	304.7	449	137,403	306.0
Fanniidae		*Euryomma sp.*	MFN	EB_1345	1	Costa Rica	Ethanol	Thorax	33.7	3,320	926,103	278.9	571	219,238	384.0
Scathophagidae		*Scathophaga stercolaria*	MFN	EB_1146	1	USA	Ethanol	Thorax	9.62	11,539	3,653,746	316.6	879	367,269	417.8
Calliphoridae	Calliphorinae	*Calliphora sp. 1*	MFN	EB_1333	6	Peru	Ethanol	Thorax	16.1	7,541	2,523,702	334.7	1,036	403,863	389.8
Calliphoridae	Calliphorinae	*Pericallimyia io*	CEUA	CEUA020	3	South Africa	Ethanol	Legs	11.1	17,829	4,630,005	259.7	843	283,063	335.8
Calliphoridae	Phumosinae	*Phumosia sp.*	CEUA	CEUA029	3	South Africa	Ethanol	Legs	8.25	2,366	759,049	320.8	833	307,507	370.5
Calliphoridae	Bengaliinae	*Bengalia sp.*	CEUA	Ken017	1	Kenya	Ethanol	Legs	10.2	2,100	762,783	363.2	951	395,844	416.2
Calliphoridae	Chrysomyinae	*Chrysomya rufifacies*	MFN	EB_1143	1	Costa Rica	Ethanol	Thorax	74.2	56,281	14,655,060	260.4	1,155	539,778	467.3
Calliphoridae	Chrysomyinae	*Protocalliphora sp.*	MFN	EB_1318	1	USA	Ethanol	Thorax	217	2,310	802,553	347.4	967	375,361	388.2
Calliphoridae	Lucilinae	*Lucilia sp. 2*	MFN	EB_1141	1	Costa Rica	Ethanol	Thorax	446	2,285	796,599	348.6	1,041	393,443	377.9
Mesembrinellidae		*Mesembrinella spicata*	MFN	EB_1153	1	Costa Rica	Ethanol	Thorax	212	2,112	747,393	353.9	974	379,846	390.0
Mesembrinellidae		*Mesembrinella batesi*	MFN	EB_976	1	Costa Rica	Ethanol	Thorax	93.1	2,081	694,354	333.7	925	344,599	372.5
Oestridae	Oestrinae	*Oestrus ovis*	USNM	USNMENT01443506	50	New Zealand	Pinned	Thorax	21	2,948	888,022	301.2	955	279,235	292.4
Oestridae	Cuterebrinae	*Dermatobia hominis*	USNM	USNMENT01443481	7	Bolivia	Pinned	Thorax	16.7	2,711	809,435	298.6	100	25,124	251.2
Oestridae	Oestrinae	*Cephenemyia sp.*	USNM	USNMENT01443476	3	USA	Pinned	Thorax	11	6,510	1,896,411	291.3	1,069	441,999	413.5
Oestridae	Cuterebrinae	*Cuterebra sp.*	MFN	EB_1184	1	USA	Ethanol	Thorax	86.7	3,879	1,188,155	306.3	913	390,083	427.3
Polleniidae	Polleniinae	*Pollenia rudis*	USNM	USNMENT01137793	2	USA	LiqNit	Thorax	306	1,572	481,692	306.4	703	227,228	322.8
Rhiniidae	Cosmininae	*Sumatria vittigera*	USNM	USNMENT01443491	35	Malaysia	Pinned	Thorax	3.42	3,046	875,701	287.5	554	163,461	294.5
Rhiniidae	Rhiniinae	*Stomorhina lunata*	CEUA	CEUA027	3	South Africa	Pinned	Thorax	86.8	3,450	1,185,126	343.5	1,068	434,558	406.9
Rhiniidae	Cosmininae	*Cosmina fuscipennis*	RMCA	RMCA006	1	South Africa	Ethanol	Thorax	590	1,784	656,122	367.8	621	191,747	308.8
Rhiniidae	Cosmininae	*Rhyncomya soyauxi*	CEUA	Ken009	1	Kenya	Ethanol	Thorax	145	3,253	1,060,963	326.1	947	371,194	392.0
Rhinophoridae		*Rhinomorinia capensis*	USNM	USNMENT01443496	40	South Africa	Pinned	Thorax	8.93	8,247	2,704,152	327.9	659	200,273	303.9
Rhinophoridae		*Tromodesia angustifrons*	USNM	USNMENT01443490	36	Israel	Pinned	Thorax	5.31	6,671	1,987,016	297.9	437	112,615	257.7
Rhinophoridae		*Melanophora roralis*	USNM	USNMENT01443501	14	USA	Pinned	Thorax	11.1	75,722	24,991,419	330.0	918	376,403	410.0
Tachinidae	Tachininae	*Spilochaetosoma californicum*	JOSC	T_1160	17	USA	Pinned	Abdome n	0.539	1,224	302,179	246.9	406	105,030	258.7
Tachinidae	Exoristinae	*Smidtia fumiferanae*	JOSC	T_1201	12	Canada	Ethanol	Legs	25.5	11,392	3,174,309	278.6	777	304,674	392.6
Tachinidae	Phasiinae	*Trichopoda pennipes*	JOSC	T_1195	6	USA	Ethanol	Legs	3.06	23,361	6,789,832	290.6	875	401,170	458.5
Tachinidae	Dexiinae	*Ptilodexia harpassa*	JOSC	T_1200	6	USA	Ethanol	Legs	3.85	25,056	8,090,213	322.9	924	396,506	429.1

Tachinidae	Tachininae	*Panzeria nigricornea*	JOSC	T_1185	1	USA	Ethanol	Legs	13.2	3,169	946,603	298.7	796	143,745	326.7
Sarcophagidae	Miltogramminae	*Amobia signata*	MFN	EB_x002	13	Italy	DNA aliquot	Legs	1.26	13,014	4,189,409	321.9	1,238	674,005	543.6
Sarcophagidae	Miltogramminae	*Eumacronychia sp.*	MFN	EB_1319	1	USA	Ethanol	Thorax	59	9,087	2,931,976	322.7	1,316	711,851	540.9
Sarcophagidae	Miltogramminae	*Sarcotachina subcylindrica*	MFN	NGSTE_UA_034	10	Ukraine	Ethanol	Legs	44.7	6,433	2,113,965	328.6	1,229	620,402	504.8
Sarcophagidae	Miltogramminae	*Sphecapatoclea sp.*	MFN	EB_1321	4	Iran	Ethanol	Thorax	101	8,048	2,629,570	326.7	1,328	655,321	493.5
Sarcophagidae	Paramacronychiinae	*Brachicoma setosa*	USNM	USNMENT01137781	2	USA	LiqNit	Thorax	6.71	3,344	1,321,419	395.2	1,086	468,292	431.2
Sarcophagidae	Paramacronychiinae	*Dexagria ushinskyi*	MFN	EB_1322	4	Iran	Ethanol	Thorax	109	6,285	2,105,180	335.0	1,315	651,477	495.4
Sarcophagidae	Paramacronychiinae	*Erythrandra distincta*	USNM	USNMENT01137800	2	USA	LiqNit	Thorax	5.07	3,894	1,514,810	389.0	1,213	591,394	487.5
Sarcophagidae	Paramacronychiinae	*Paramacronychia flavipalpis*	MFN	EB_1323	3	?	Ethanol	Thorax	14.1	8,758	2,918,373	333.2	1,356	660,841	487.3
Sarcophagidae	Sarcophaginae	*Argoravinia rufiventris*	MFN	EB_617	2	Costa Rica	Ethanol	Thorax	189	17,241	4,662,388	270.4	1,460	693,156	474.8
Sarcophagidae	Sarcophaginae	*Blaesoxipha (Acanthodotheca) reperta*	USNM	USNMENT01137758	2	USA	LiqNit	Thorax	124	2,570	1,062,409	413.4	1,492	601,430	403.1
Sarcophagidae	Sarcophaginae	*Blaesoxipha (Gigantotheca) plinthopyga*	USNM	USNMENT01137736	2	USA	LiqNit	Thorax	590	2,418	1,019,610	421.7	1,438	592,484	412.0
Sarcophagidae	Sarcophaginae	*Boettcheria latisterna*	USNM	USNMENT01137774	2	USA	LiqNit	Thorax	187	2,653	1,040,151	392.1	1,456	576,097	395.7
Sarcophagidae	Sarcophaginae	*Boettcheria praevolans*	MFN	EB_905	4	Costa Rica	Ethanol	Thorax	570	4,639	1,609,915	347.0	1,371	583,943	425.9
Sarcophagidae	Sarcophaginae	*Chrysagria andina*	USNM	USNMENT01443519	40	Ecuador	Pinned	Thorax	5.09	5,803	1,862,429	320.9	1,333	535,331	401.6
Sarcophagidae	Sarcophaginae	*Cistudinomyia cistudinis*	USNM	USNMENT01443516	48	USA	Pinned	Thorax	6.41	3,664	1,177,263	321.3	1,297	415,160	320.1
Sarcophagidae	Sarcophaginae	*Comasarcophaga texana*	USNM	USNMENT01443518	33	USA	Pinned	Thorax	8.57	6,359	2,167,504	340.9	1,453	706,200	486.0
Sarcophagidae	Sarcophaginae	*Dexosarcophaga transita*	MFN	EB_1326	5	Peru	Ethanol	Thorax	472	4,675	1,911,607	408.9	1,452	688,855	474.4
Sarcophagidae	Sarcophaginae	*Emblemasoma faciale*	USNM	USNMENT01443510	28	USA	Pinned	Thorax	7.89	7,056	2,452,906	347.6	1,305	677,742	519.3
Sarcophagidae	Sarcophaginae	*Emblemasoma sp.*	USNM	USNMENT01443525	16	Bolivia	Pinned	Thorax	9.91	9,400	3,108,609	330.7	1,426	791,036	554.7
Sarcophagidae	Sarcophaginae	*Emdenimyia limai*	MFN	EB_1328	5	Peru	Ethanol	Thorax	131	8,361	2,761,948	330.3	1,407	729,186	518.3
Sarcophagidae	Sarcophaginae	*Engelimyia inops*	MFN	EB_615	2	Costa Rica	Ethanol	Thorax	411	2,679	1,228,042	458.4	1,475	676,521	458.7
Sarcophagidae	Sarcophaginae	*Engelimyia sp.*	USNM	USNMENT01443532	16	Bolivia	Pinned	Legs	8.68	7,752	2,666,788	344.0	1,367	722,032	528.2
Sarcophagidae	Sarcophaginae	*Fletcherimyia folkertsi*	USNM	USNMENT01443517	18	USA	Pinned	Legs	6.69	8,636	3,068,861	355.4	1,187	622,165	523.7
Sarcophagidae	Sarcophaginae	*Fletcherimyia oreophilae*	USNM	USNMENT01443533	18	USA	Pinned	Legs	9.53	18,750	5,590,390	298.2	1,457	773,165	530.7
Sarcophagidae	Sarcophaginae	*Helicobia morionella*	MFN	EB_595	2	Costa Rica	Ethanol	Thorax	84	3,442	1,576,366	458.0	1,412	747,268	529.2
Sarcophagidae	Sarcophaginae	*Helicobia rapax*	USNM	USNMENT01410968	1	USA	LiqNit	Thorax	43.4	2,688	1,162,288	432.4	1,428	607,595	425.5
Sarcophagidae	Sarcophaginae	*Lepidodexia (Neophyto) sheldoni*	USNM	USNMENT00191947	16	USA	Pinned	Thorax	17.4	11,184	3,872,699	346.3	1,430	688,332	481.4
Sarcophagidae	Sarcophaginae	*Lepidodexia (Notochaeta) sp.*	MFN	EB_893	1	Costa Rica	Ethanol	Thorax	406	2,838	1,019,291	359.2	1,193	444,827	372.9
Sarcophagidae	Sarcophaginae	*Lepidodexia (Notochaeta) woodi*	MFN	EB_865	8	Costa Rica	Ethanol	Thorax	322	33,841	9,255,566	273.5	1,461	697,102	477.1
Sarcophagidae	Sarcophaginae	*Lipoptilocnema koehleri*	MFN	EB_x287	6	Brazil	DNA aliquot	Body	36.1	20,310	5,846,842	287.9	1,322	819,083	619.6
Sarcophagidae	Sarcophaginae	*Lipoptilocnema lanei*	MFN	EB_x285	7	Brazil	DNA aliquot	Body	25.1	29,808	8,187,327	274.7	1,323	826,933	625.0
Sarcophagidae	Sarcophaginae	*Malacophagomyia filamenta*	MFN	EB_570	2	Venezuela	Ethanol	Thorax	144	29,322	7,705,446	262.8	1,410	710,899	504.2
Sarcophagidae	Sarcophaginae	*Malacophagomyia sp.C*	USNM	USNMENT01443524	42	Venezuela	Pinned	Thorax	3.51	7,185	2,284,056	317.9	1,152	424,098	368.5
Sarcophagidae	Sarcophaginae	*Mecynocorpus salvum*	USNM	USNMENT01443528	54	USA	Pinned	Thorax	7.54	7,101	2,270,700	319.8	1,115	371,873	333.8
Sarcophagidae	Sarcophaginae	*Microcerella sp.*	MFN	EB_1332	6	Peru	Ethanol	Thorax	416	3,447	1,430,145	414.9	1,434	673,397	469.6
Sarcophagidae	Sarcophaginae	*Microcerella halli*	MFN	EB_1330	7	Peru	Ethanol	Thorax	101	7,130	2,512,538	352.4	1,388	780,613	562.4

Sarcophagidae	Sarcophaginae	*Nephochaetopteryx orbitalis*	MFN	EB_904	1	Costa Rica	Ethanol	Thorax	104	7,013	2,505,706	357.3	1,389	771,316	554.9
Sarcophagidae	Sarcophaginae	*Nephochaetopteryx sp.*	MFN	EB_1338	5	Peru	Ethanol	Thorax	92.7	5,688	2,141,011	376.4	1,438	741,607	515.7
Sarcophagidae	Sarcophaginae	*Oxysarcodexia thornax*	MFN	EB_576	2	Costa Rica	Ethanol	Thorax	344	18,293	5,154,854	281.8	1,445	702,212	486.0
Sarcophagidae	Sarcophaginae	*Oxysarcodexia trivialis*	MFN	EB_907	4	Costa Rica	Ethanol	Thorax	363	2,791	939,824	336.7	1,329	452,793	340.7
Sarcophagidae	Sarcophaginae	*Oxysarcodexia ventricosa*	USNM	USNMENT01410964	1	USA	LiqNit	Thorax	127	3,400	1,474,377	433.6	1,423	683,063	480.0
Sarcophagidae	Sarcophaginae	*Oxyvinia sp.*	MFN	EB_1344	5	Peru	Ethanol	Thorax	12.5	5,013	1,699,980	339.1	1,523	573,869	376.8
Sarcophagidae	Sarcophaginae	*Oxyvinia sp. dexo*	MFN	EB_1324	6	Peru	Ethanol	Thorax	354	6,048	2,232,545	369.1	1,379	736,973	534.4
Sarcophagidae	Sarcophaginae	*Oxyvinia wicharti*	MFN	EB_863	5	Peru	Ethanol	Thorax	116	3,057	1,379,663	451.3	1,426	688,368	482.4
Sarcophagidae	Sarcophaginae	*Peckia (Euboettcheria) anguilla*	MFN	EB_1150	1	Costa Rica	Ethanol	Thorax	172	5,760	2,220,560	385.5	1,358	764,257	562.8
Sarcophagidae	Sarcophaginae	*Peckia (Euboettcheria) collusor*	MFN	EB_851	1	Guatemala	Ethanol	Thorax	336	21,328	5,749,653	269.6	1,413	732,568	518.4
Sarcophagidae	Sarcophaginae	*Peckia (Pattonella) intermutans*	MFN	EB_937	1	Costa Rica	Ethanol	Thorax	76.7	5,511	2,159,373	391.8	1,371	776,137	565.7
Sarcophagidae	Sarcophaginae	*Peckia (Peckia) gulo*	MFN	EB_1017	1	Costa Rica	Ethanol	Thorax	428	2,601	1,195,638	459.7	1,449	664,808	458.8
Sarcophagidae	Sarcophaginae	*Peckia (Sarcodexia) aequata*	MFN	EB_1149	1	Costa Rica	Ethanol	Thorax	309	2,815	1,304,433	463.4	1,446	693,288	479.5
Sarcophagidae	Sarcophaginae	*Peckia (Sarcodexia) lambens*	USNM	USNMENT01410983	1	USA	LiqNit	Thorax	217	4,111	1,749,001	425.4	1,316	684,252	519.9
Sarcophagidae	Sarcophaginae	*Peckia (Squamatodes) ingens*	MFN	EB_941	1	Costa Rica	Ethanol	Thorax	310	3,424	1,485,802	433.9	1,408	693,173	492.3
Sarcophagidae	Sarcophaginae	*Peckiamyia calx*	MFN	EB_895	1	Costa Rica	Ethanol	Thorax	156	3,908	1,650,894	422.4	1,447	748,065	517.0
Sarcophagidae	Sarcophaginae	*Rafaelia ampulla*	USNM	USNMENT01443520	22	USA	Pinned	Thorax	9	11,491	3,479,550	302.8	1,417	710,976	501.7
Sarcophagidae	Sarcophaginae	*Ravinia derelicta*	USNM	USNMENT01410963	1	USA	LiqNit	Thorax	233	2,861	1,242,508	434.3	1,416	643,405	454.4
Sarcophagidae	Sarcophaginae	*Ravinia querula*	USNM	USNMENT01137267	2	USA	LiqNit	Thorax	510	2,994	1,295,253	432.6	1,413	637,800	451.4
Sarcophagidae	Sarcophaginae	*Retrocitomyia sp.*	MFN	EB_1212	5	Peru	Ethanol	Thorax	108	3,711	1,590,884	428.7	1,414	731,420	517.3
Sarcophagidae	Sarcophaginae	*Sarcofahrtiopsis cuneata*	MFN	EB_575	2	Costa Rica	Ethanol	Thorax	91.9	18,249	5,119,082	280.5	1,410	650,692	461.5
Sarcophagidae	Sarcophaginae	*Sarcofahrtiopsis paterna*	USNM	USNMENT01410959	1	USA	LiqNit	Thorax	63	4,566	1,764,414	386.4	1,380	687,878	498.5
Sarcophagidae	Sarcophaginae	*Sarcophaga (Aethiopisca) currani*	NHMD	ZMUC00036119	6	Uganda	DNA aliquot	Legs	1.99	16,783	5,179,072	308.6	1,331	760,992	571.7
Sarcophagidae	Sarcophaginae	*Sarcophaga (Asceloctella) australis*	NHMD	ZMUC00036135	6	Australia	DNA aliquot	Legs	1.23	18,706	5,123,686	273.9	1,359	605,385	445.5
Sarcophagidae	Sarcophaginae	*Sarcophaga (Asceloctella) calicifera*	NHMD	ZMUC00022812	7	Burundi	DNA aliquot	Legs	1.25	6,253	1,730,512	276.7	1,218	418,221	343.9
Sarcophagidae	Sarcophaginae	*Sarcophaga (Bellieromima) subulata*	NHMD	ZMUC00022919	6	Croatia	DNA aliquot	Legs	1.67	22,351	6,679,612	298.9	1,496	721,240	482.1
Sarcophagidae	Sarcophaginae	*Sarcophaga (Bercaea) africa*	USNM	USNMENT01137691	2	Denmark	Ethanol	Thorax	19.4	8,624	2,569,958	298.0	1,451	549,286	378.6
Sarcophagidae	Sarcophaginae	*Sarcophaga (Bercaea) arno*	NHMD	ZMUC00022867	7	Burundi	DNA aliquot	Legs	1.17	12,678	3,585,220	282.8	1,481	658,975	445.0
Sarcophagidae	Sarcophaginae	*Sarcophaga (Bezziella) cfvicaria*	NHMD	ZMUC00022810	9	Tanzania	DNA aliquot	Legs	1.31	10,473	3,019,497	288.3	1,379	567,899	411.8
Sarcophagidae	Sarcophaginae	*Sarcophaga (Brasia) booersiana*	NHMD	ZMUC00036198	9	Tanzania	DNA aliquot	Legs	5.41	19,118	5,403,205	282.6	1,479	681,365	460.7
Sarcophagidae	Sarcophaginae	*Sarcophaga (Curranisca) chapini*	NHMD	ZMUC00022854	7	Burundi	DNA aliquot	Legs	3.72	22,512	6,645,517	295.2	1,431	696,507	486.7
Sarcophagidae	Sarcophaginae	*Sarcophaga (Danbeckia) paralina*	NHMD	ZMUC00022878	7	Kenya	DNA aliquot	Legs	4.35	14,477	4,142,361	286.1	1,416	637,213	450.0
Sarcophagidae	Sarcophaginae	*Sarcophaga (Helicophagella) noverca*	NHMD	ZMUC00036055	6	Croatia	DNA aliquot	Legs	1.11	2,861	928,456	324.5	585	153,095	261.7
Sarcophagidae	Sarcophaginae	*Sarcophaga (Hellicophagella) melanura*	USNM	USNMENT01137742	2	Denmark	Ethanol	Thorax	25.8	4,160	1,424,645	342.5	1,165	396,597	340.4
Sarcophagidae	Sarcophaginae	*Sarcophaga (Heteronychia) haemorrhoides*	NHMD	ZMUC00036020	6	Croatia	DNA aliquot	Legs	4.11	8,419	3,262,131	387.5	800	218,475	273.1

Sarcophagidae	Sarcophaginae	*Sarcophaga (Heteronychia) schineri*	NHMD	ZMUC00036030	6	Croatia	DNA aliquot	Legs	3.45	22,024	7,999,417	363.2	1,194	331,337	277.5
Sarcophagidae	Sarcophaginae	*Sarcophaga (Hyperacanthisca) zumpti*	NHMD	ZMUC00022813	7	Burundi	DNA aliquot	Legs	1.3	20,206	6,519,787	322.7	1,247	364,180	292.0
Sarcophagidae	Sarcophaginae	*Sarcophaga (Liopygia) crassipalpis*	USNM	USNMENT01137240	2	USA	LiqNit	Thorax	48	3,111	1,606,162	516.3	1,414	792,115	560.2
Sarcophagidae	Sarcophaginae	*Sarcophaga (Liopygia) par*	NHMD	ZMUC00022832	7	Burundi	DNA aliquot	Legs	2.63	2,151	642,385	298.6	734	205,132	279.5
Sarcophagidae	Sarcophaginae	*Sarcophaga (Liosarcophaga) emdeni*	USNM	USNMENT01137647	2	Denmark	Ethanol	Thorax	42.1	10,835	3,381,282	312.1	1,468	503,765	343.2
Sarcophagidae	Sarcophaginae	*Sarcophaga (Liosarcophaga) redux*	NHMD	ZMUC00022877	7	Kenya	DNA aliquot	Legs	1.42	4,042	1,433,080	354.5	763	225,710	295.8
Sarcophagidae	Sarcophaginae	*Sarcophaga (Mauritiella) cfrayssae*	NHMD	ZMUC00036120	6	Uganda	DNA aliquot	Legs	2.99	10,299	3,761,552	365.2	1,099	338,418	307.9
Sarcophagidae	Sarcophaginae	*Sarcophaga (Mehria) sexpunctata*	NHMD	ZMUC00022936	6	Croatia	DNA aliquot	Legs	1.17	14,608	5,110,547	349.8	1,285	672,130	523.1
Sarcophagidae	Sarcophaginae	*Sarcophaga (Myorhina) lunigera*	NHMD	ZMUC00022942	6	Croatia	DNA aliquot	Legs	2.16	17,447	5,788,814	331.8	1,353	787,280	581.9
Sarcophagidae	Sarcophaginae	*Sarcophaga (Neobellieria) bullata*	USNM	USNMENT01410973	1	USA	LiqNit	Thorax	244	2,555	1,010,602	395.5	1,422	557,250	391.9
Sarcophagidae	Sarcophaginae	*Sarcophaga (Neosarcophaga) occidentalis*	USNM	USNMENT01137184	2	USA	LiqNit	Thorax	463	27,804	8,158,254	293.4	1,412	757,964	536.8
Sarcophagidae	Sarcophaginae	*Sarcophaga (Pandelleana) insularis*	NHMD	ZMUC00036107	6	France	DNA aliquot	Legs	3.82	29,692	8,794,109	296.2	1,381	819,403	593.3
Sarcophagidae	Sarcophaginae	*Sarcophaga (Pandelleana) protuberans*	NHMD	ZMUC00022886	7	Turkey	DNA aliquot	Legs	1.45	15,997	5,236,787	327.4	1,392	802,446	576.5
Sarcophagidae	Sarcophaginae	*Sarcophaga (Paraethiopisca) dewulfi*	NHMD	ZMUC00022870	7	Burundi	DNA aliquot	Legs	4.41	10,548	3,719,236	352.6	1,366	724,429	530.3
Sarcophagidae	Sarcophaginae	*Sarcophaga (Parasarcophaga) albiceps*	MFN	Kiev_2007 #2	11	Ukraine	DNA aliquot	Legs	1.06	10,167	3,519,082	346.1	1,364	754,623	553.2
Sarcophagidae	Sarcophaginae	*Sarcophaga (Parasarcophaga) hirtipes*	NHMD	ZMUC00022828	7	Burundi	DNA aliquot	Legs	5.47	5,209	2,071,528	397.7	1,380	692,673	503.0
Sarcophagidae	Sarcophaginae	*Sarcophaga (Robineauella) caerulescens*	USNM	USNMENT01137715	2	Denmark	Ethanol	Thorax	75.9	3,861	1,595,681	413.3	1,464	685,886	468.5
Sarcophagidae	Sarcophaginae	*Sarcophaga (Rohdendorfisca) forma*	MFN	EB_x045	13	United Arab Emirates	DNA aliquot	Legs	2.94	20,689	6,083,331	294.0	1,424	832,518	584.6
Sarcophagidae	Sarcophaginae	*Sarcophaga (Rosellea) aratrix*	NHMD	ZMUC00036051	6	Croatia	DNA aliquot	Legs	2.32	10,853	4,062,798	374.3	1,394	771,819	553.7
Sarcophagidae	Sarcophaginae	*Sarcophaga (Rosellea) beckiana*	NHMD	ZMUC00022883	6	Turkey	DNA aliquot	Legs	2.35	13,332	4,846,234	363.5	1,360	787,821	579.7
Sarcophagidae	Sarcophaginae	*Sarcophaga (Sarcophaga) lehmanni*	USNM	USNMENT01137756	2	Denmark	Ethanol	Thorax	17.1	7,151	2,334,800	326.5	1,713	587,553	343.0
Sarcophagidae	Sarcophaginae	*Sarcophaga (Sarcophaga) variegata*	USNM	USNMENT01137709	2	Denmark	Ethanol	Thorax	129	2,409	776,892	322.5	1,615	492,610	305.0
Sarcophagidae	Sarcophaginae	*Sarcophaga (Sarcorohdendorfia) furcata*	NHMD	ZMUC00036146	6	Australia	DNA aliquot	Legs	2.32	19,467	5,863,997	301.2	1,374	807,727	587.9
Sarcophagidae	Sarcophaginae	*Sarcophaga (Sarcorohdendorfia) spinigera*	NHMD	ZMUC00036159	6	Australia	DNA aliquot	Legs	2.03	9,747	3,336,966	342.4	1,248	672,045	538.5
Sarcophagidae	Sarcophaginae	*Sarcophaga (Sarcosolomonia) crinita*	USNM	USNMENT01147992	3	Myanmar	Ethanol	Thorax	242	2,487	604,212	242.9	597	158,625	265.7
Sarcophagidae	Sarcophaginae	*Sarcophaga (Stackelbergeola) mehadiensis*	NHMD	ZMUC00036034	6	Croatia	DNA aliquot	Legs	2.54	24,717	7,810,831	316.0	1,385	846,000	610.8
Sarcophagidae	Sarcophaginae	*Sarcophaga (Thyrsocnema) incisilobata*	USNM	USNMENT01137746	2	Denmark	Ethanol	Thorax	27.2	5,279	1,470,350	278.5	1,129	308,764	273.5
Sarcophagidae	Sarcophaginae	*Sarcophaga (Thyrsocnema) platariae*	MFN	EB_1152	1	Turkey	Ethanol	Thorax	400	4,080	1,789,371	438.6	1,388	747,130	538.3
Sarcophagidae	Sarcophaginae	*Spirobolomyia flavipalpis*	USNM	USNMENT01443565	1	USA	Ethanol	Thorax	330	3,012	1,356,472	450.4	1,368	655,064	478.8
Sarcophagidae	Sarcophaginae	*Spirobolomyia singularis*	USNM	USNMENT01411040	1	USA	LiqNit	Thorax	231	2,788	1,201,541	431.0	1,421	632,300	445.0
Sarcophagidae	Sarcophaginae	*Titanogrypa (Cucullomyia) placida*	MFN	EB_1211	5	Costa Rica	Ethanol	Thorax	15.1	13,603	4,195,698	308.4	1,380	783,317	567.6
Sarcophagidae	Sarcophaginae	*Titanogrypa (Titanogrypa) melampyga*	USNM	USNMENT00091605	18	USA	Pinned	Thorax	8.38	27,087	7,419,417	273.9	1,269	681,347	536.9
Sarcophagidae	Sarcophaginae	*Tricharaea (Sarcophagula) occidua*	MFN	EB_1021	1	Costa Rica	Ethanol	Thorax	8.32	7,838	2,673,067	341.0	1,387	649,059	468.3

Sarcophagidae	Sarcophaginae	*Tricharaea (Sarothromyia) simplex*	MFN	EB_1151	1	Costa Rica	Ethanol	Thorax	9.3	7,833	2,571,997	328.4	1,374	666,788	485.6
Sarcophagidae	Sarcophaginae	*Tripanurga importuna*	USNM	USNMENT01443483	23	USA	Pinned	Thorax	23.5	4,486	1,453,943	324.1	1,465	450,026	307.2
Sarcophagidae	Sarcophaginae	*Tripanurga sp.*	MFN	EB_1208	1	USA	Ethanol	Thorax	49	5,633	2,039,148	362.0	1,388	609,075	439.1
Sarcophagidae	Sarcophaginae	*Tulaeopoda pervillosa*	USNM	USNMENT01443507	52	Bahamas	Pinned	Legs	0.328	1,070	266,135	248.7	196	45,171	230.5
Sarcophagidae	Sarcophaginae	*Udamopyga iku*	MFN	EB_862	5	Colombia	Ethanol	Thorax	433	2,383	949,602	398.5	1,350	530,638	393.1
Sarcophagidae	Sarcophaginae	*Villegasia postuncinata*	MFN	EB_1028	1	Costa Rica	Ethanol	Thorax	41.2	8,131	2,790,530	343.2	1,433	711,872	496.8
