# Supplementary material for: Museomics and phylogenomics with protein-encoding ultraconserved elements illuminate the evolution of life history and phallic morphology of flesh flies (Diptera: Sarcophagidae)
Source: BMC Ecol Evol. 2021 Apr 28;21:70. doi: 10.1186/s12862-021-01797-7 (PMC8082969; doi:10.1186/s12862-021-01797-7)

Additional file 2. Phylogenetic relationships for Sarcophagidae and 10 other Calyptratae fly families inferred from 17 datasets having varying loci occupancy and coded as nucleotides and amino acids using a concatenated ML approach as well as by reconstructing a species tree estimated from UCE gene trees. Datasets are described in Table 1.

2.1. Phylogeny of Sarcophagidae. RAxML best tree (topology A) estimated from the concatenated dataset (**analysis number 1**, see Table 1) having 0.10 of occupancy and including 2,018 UCE loci coded as nucleotides with support values from 100 RAxML bootstrap analyses mapped on the respective nodes.

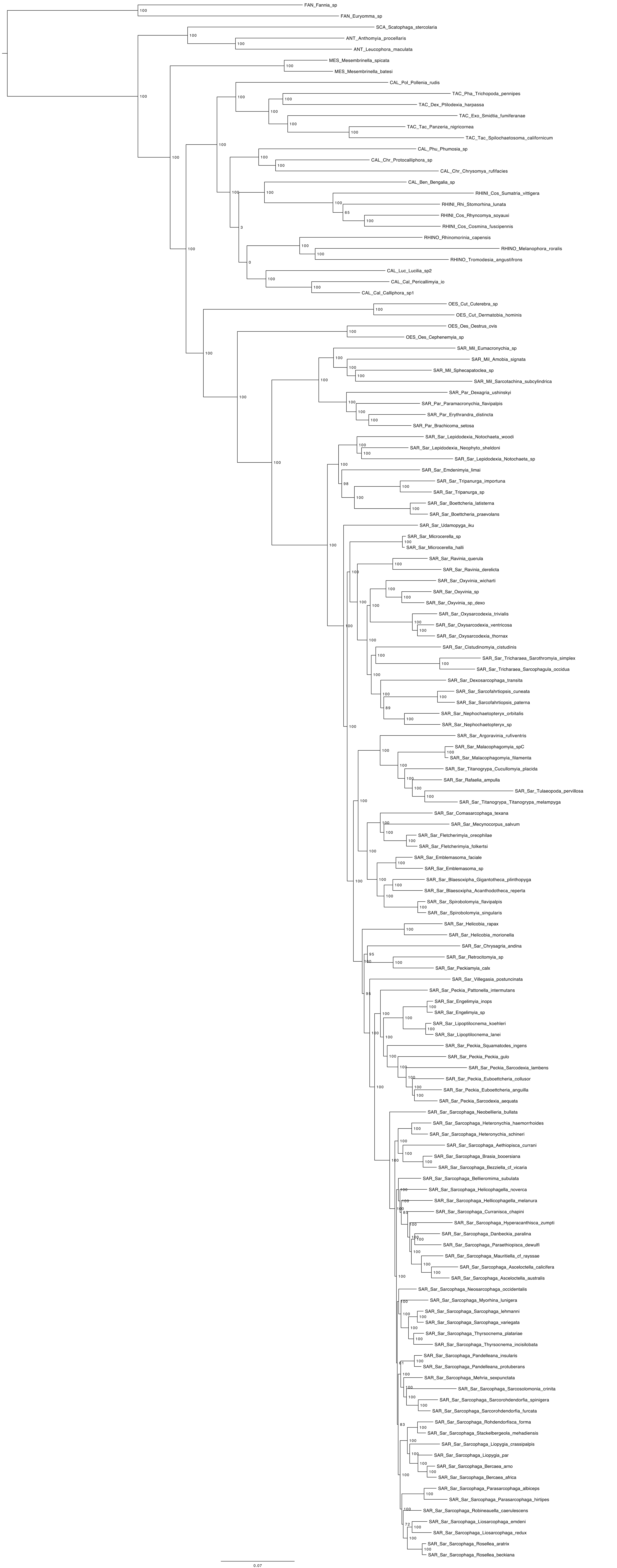

2.2. Phylogeny of Sarcophagidae. RAxML best tree (topology A) estimated from the concatenated dataset (**analysis number 2**, see Table 1) having 0.20 of occupancy and including 1,778 UCE loci coded as nucleotides with support values from 100 RAxML bootstrap analyses mapped on the respective nodes.

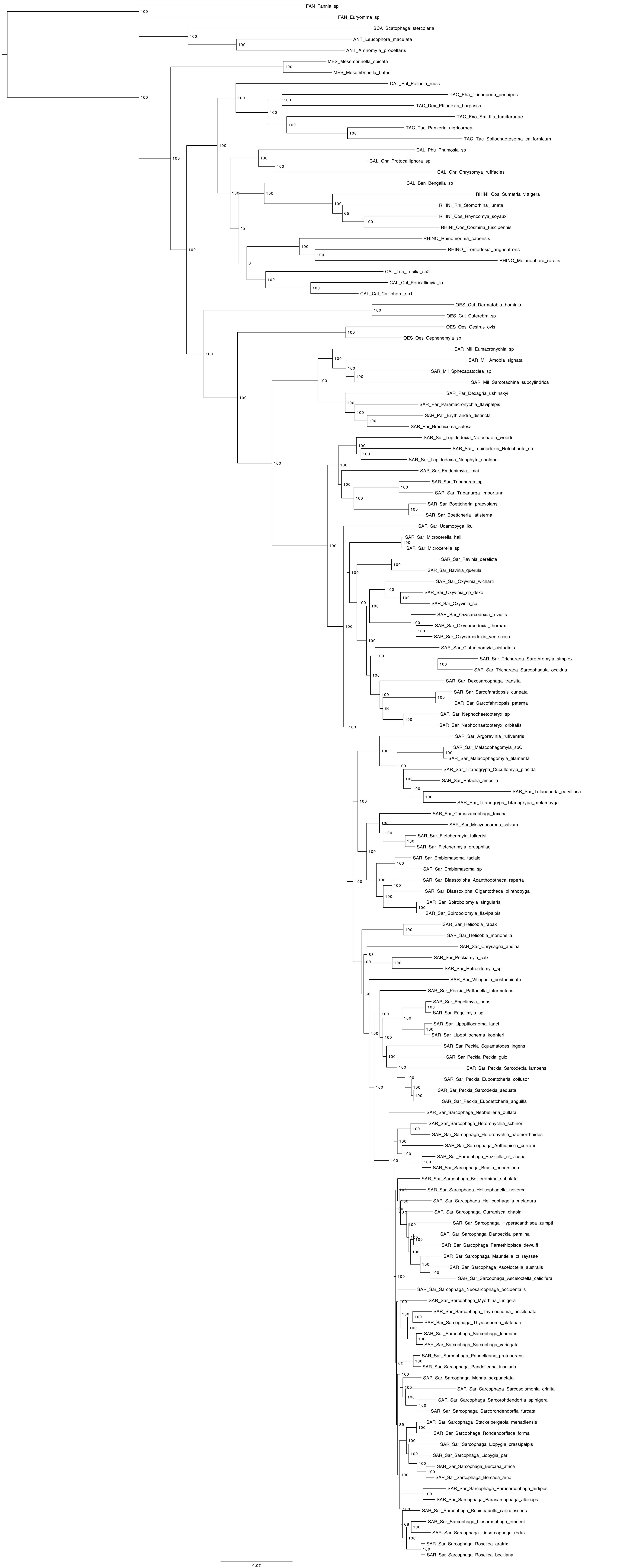

2.3. Phylogeny of Sarcophagidae. RAxML best tree (topology A) estimated from the concatenated dataset (**analysis number 3**, see Table 1) having 0.30 of occupancy and including 1,634 UCE loci coded as nucleotides with support values from 100 RAxML bootstrap analyses mapped on the respective nodes.

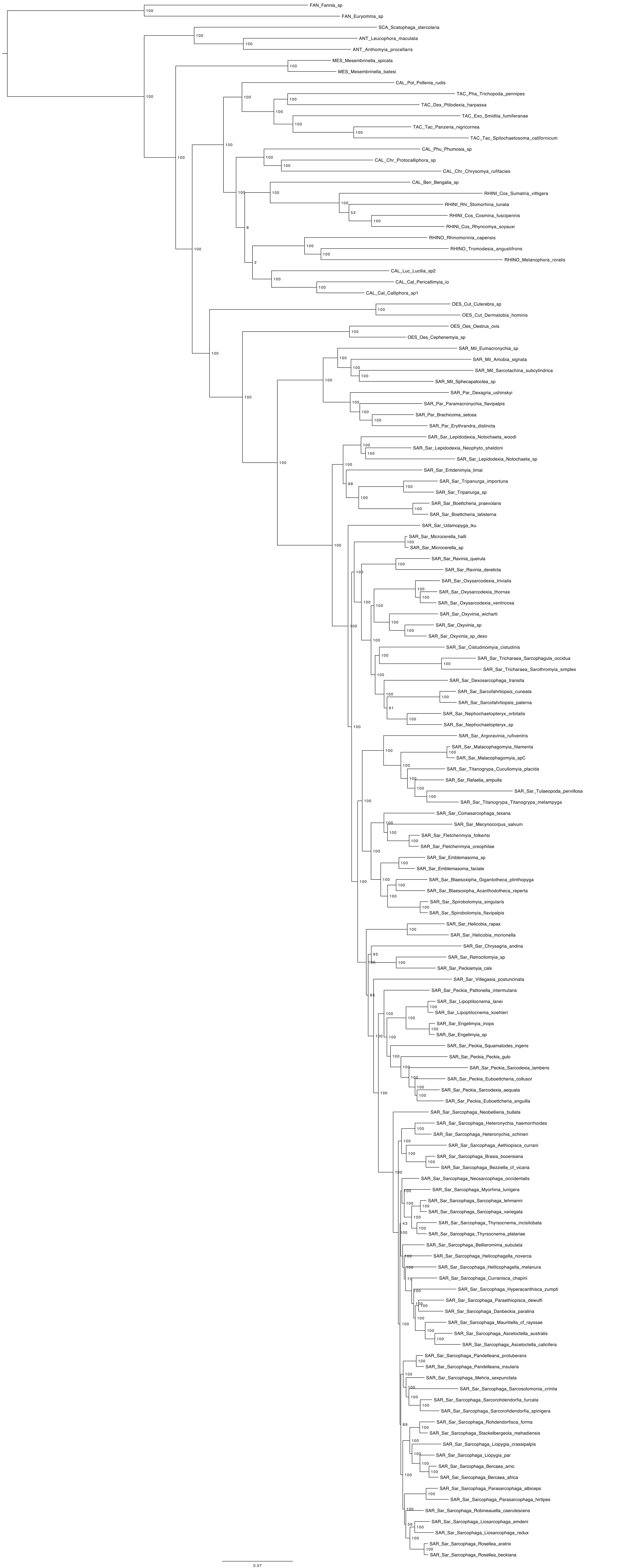

2.4. Phylogeny of Sarcophagidae. RAxML best tree (topology A) estimated from the concatenated dataset (**analysis number 4**, see Table 1) having 0.40 of occupancy and including 1,524 UCE loci coded as nucleotides with support values from 100 RAxML bootstrap analyses mapped on the respective nodes.

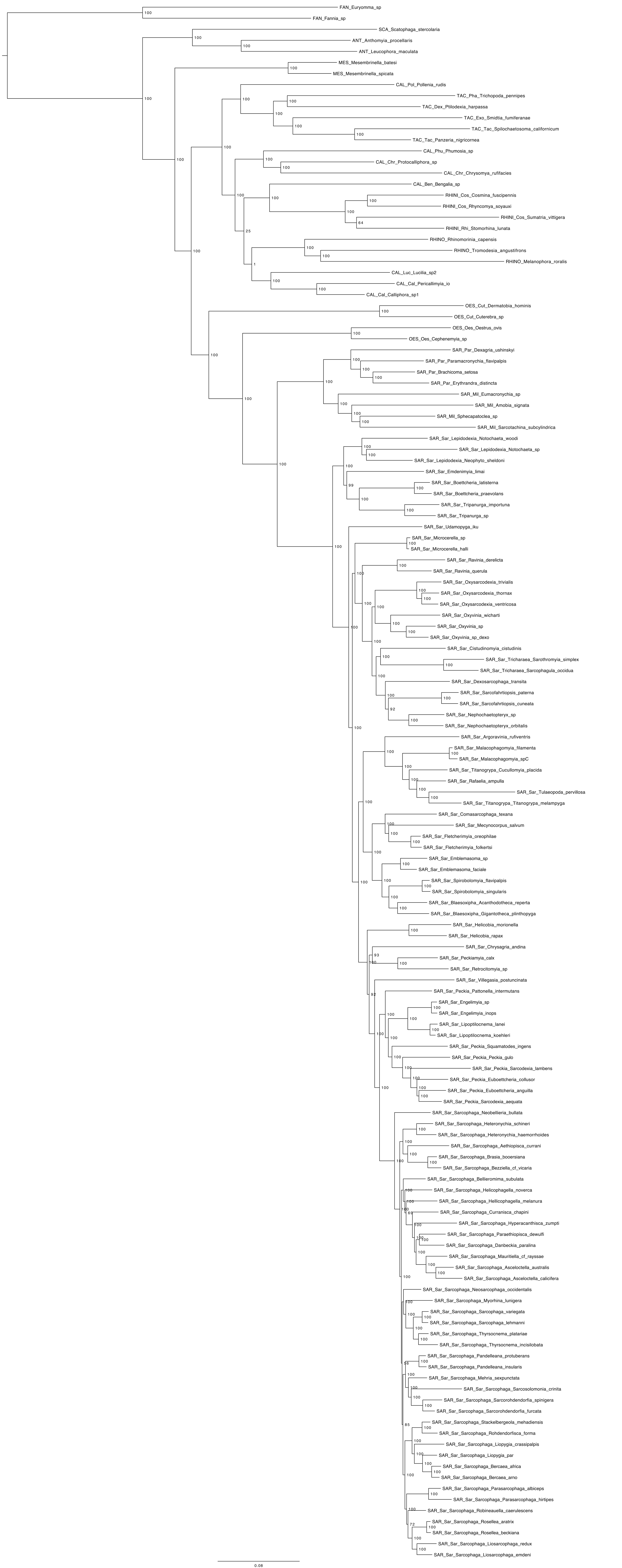

2.5. Phylogeny of Sarcophagidae. RAxML best tree (topology A) estimated from the concatenated dataset (**analysis number 5**, see Table 1) having 0.50 of occupancy and including 1,417 UCE loci coded as nucleotides with support values from 100 RAxML bootstrap analyses mapped on the respective nodes.

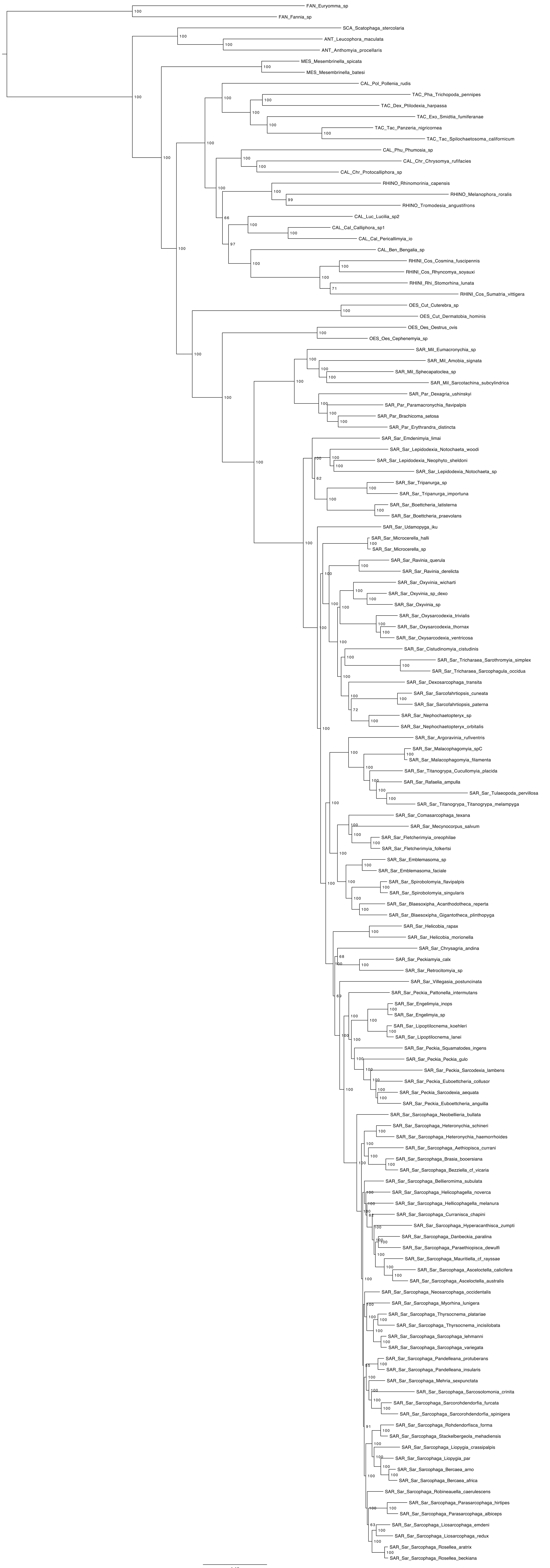

2.6. Phylogeny of Sarcophagidae. RAxML best tree (topology A) estimated from the concatenated dataset (**analysis number 6**, see Table 1) having 0.60 of occupancy and including 1,271 UCE loci coded as nucleotides with support values from 100 RAxML bootstrap analyses mapped on the respective nodes.

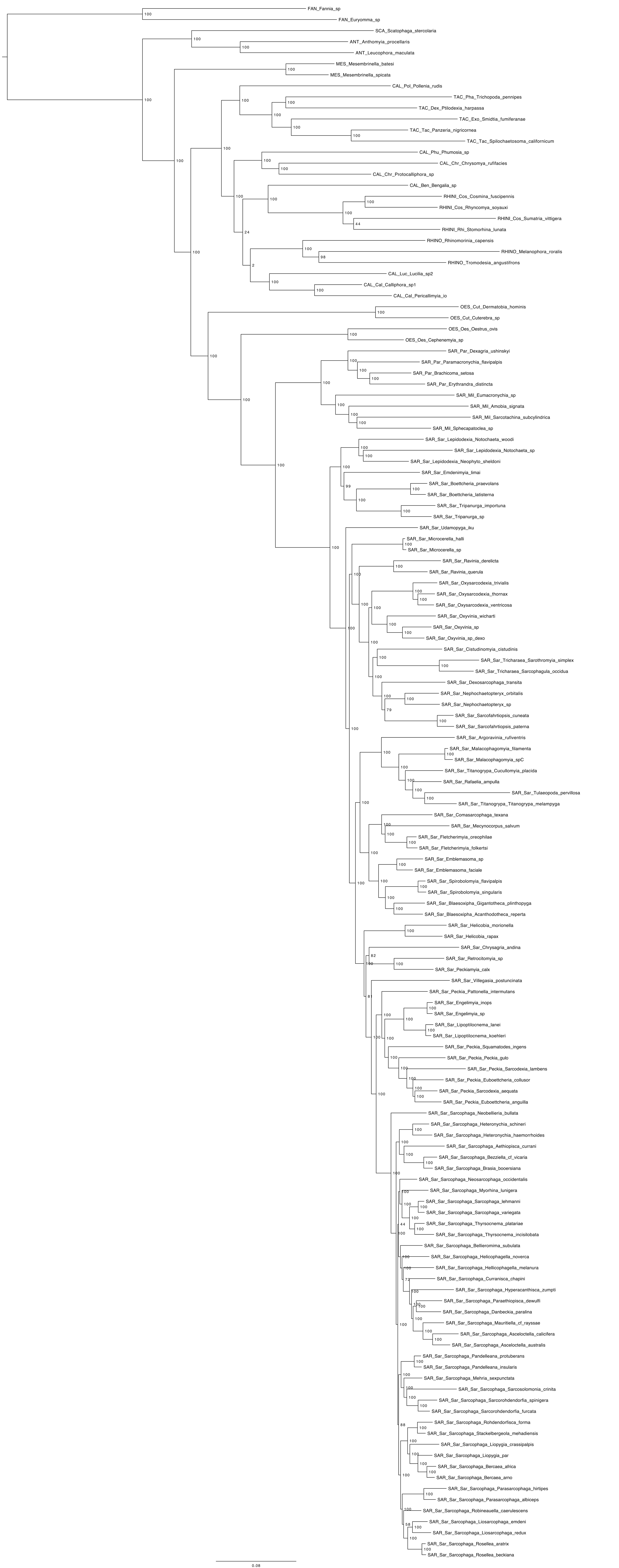

2.7. Phylogeny of Sarcophagidae. RAxML best tree (topology int.) estimated from the concatenated dataset (**analysis number 7**, see Table 1) having 0.70 of occupancy and including 936 UCE loci coded as nucleotides with support values from 100 RAxML bootstrap analyses mapped on the respective nodes.

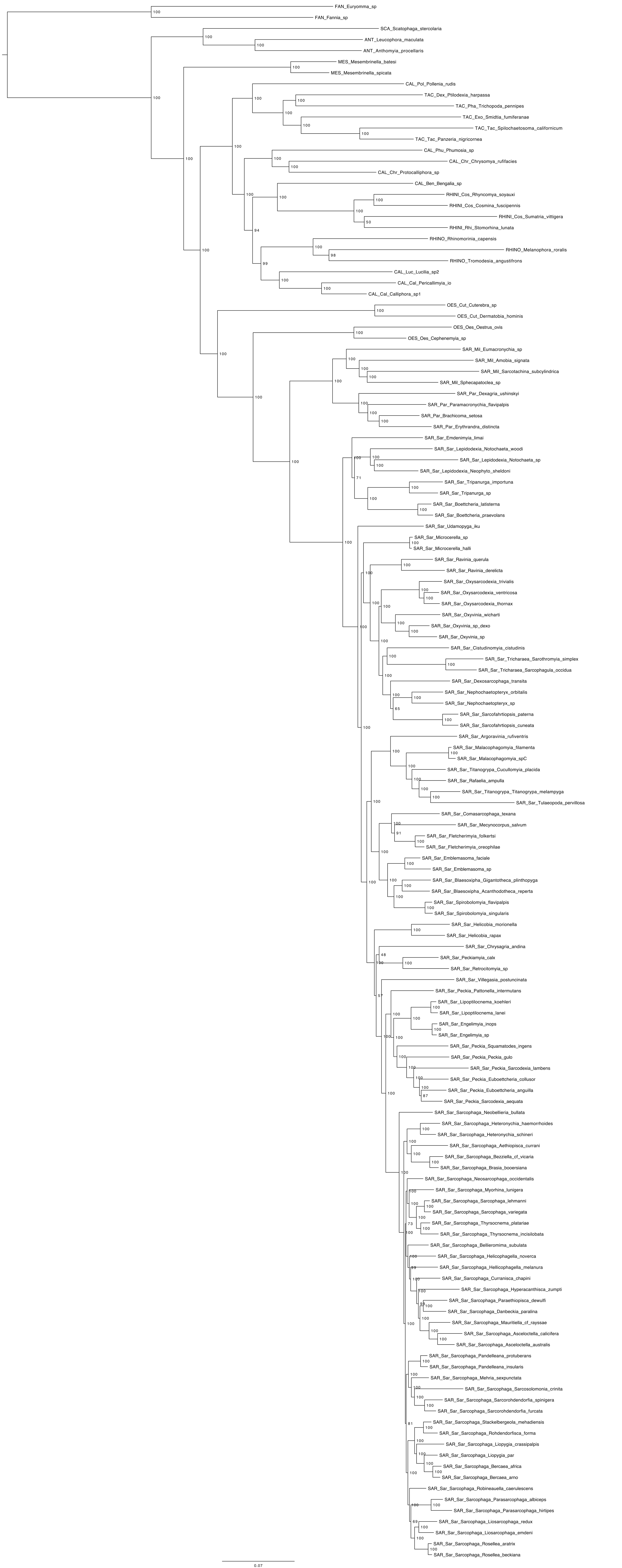

2.8. Phylogeny of Sarcophagidae. RAxML best tree (topology int.) estimated from the concatenated dataset (**analysis number 8**, see Table 1) having 0.80 of occupancy and including 440 UCE loci coded as nucleotides with support values from 100 RAxML bootstrap analyses mapped on the respective nodes.

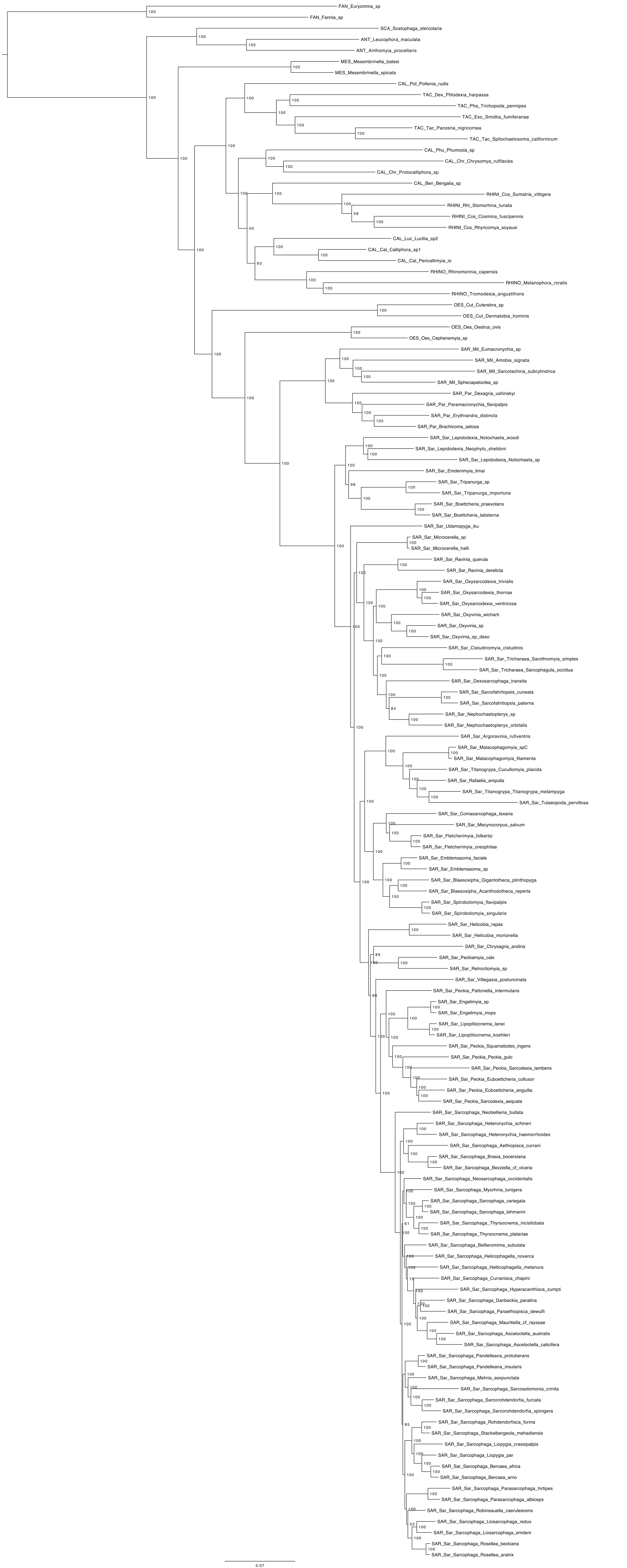

2.9. Phylogeny of Sarcophagidae. RAxML best tree (topology B) estimated from the concatenated dataset (**analysis number 9**, see Table 1) having 0.90 of occupancy and including 59 UCE loci coded as nucleotides with support values from 100 RAxML bootstrap analyses mapped on the respective nodes.

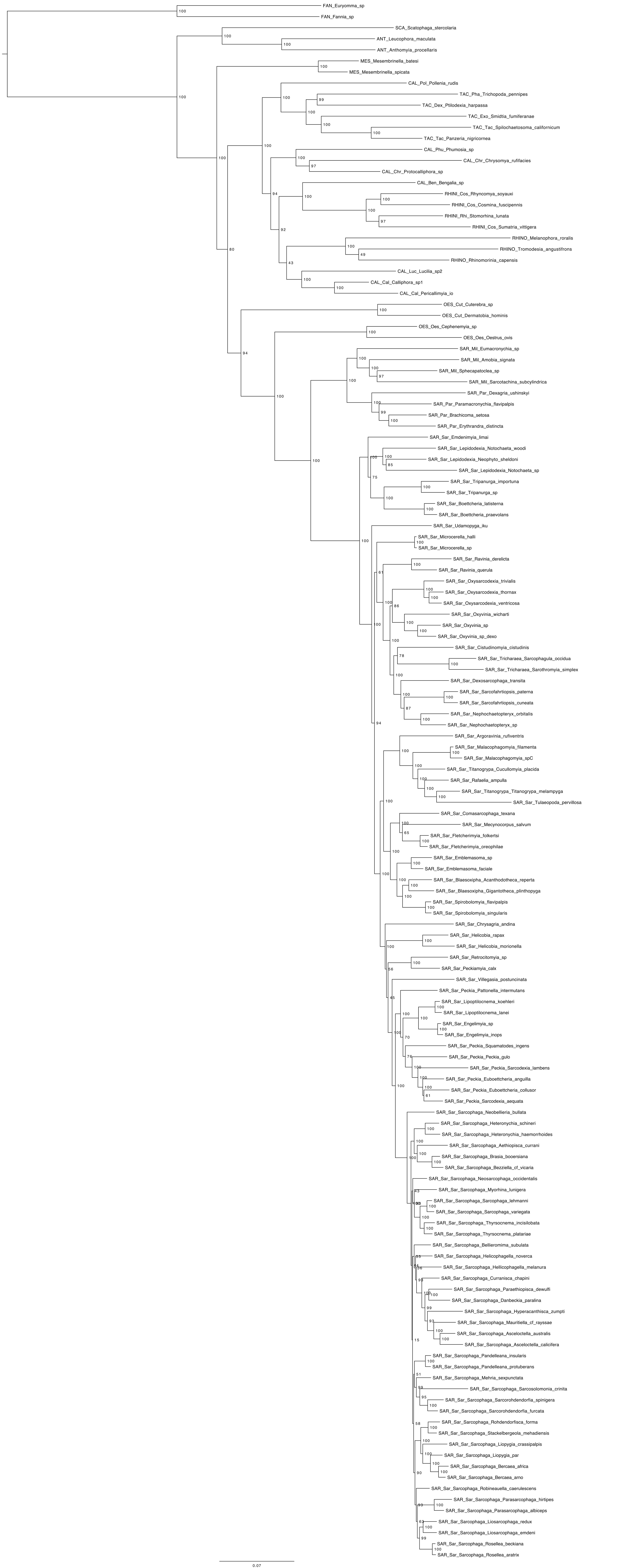

2.10. Phylogeny of Sarcophagidae. Species tree (topology A) estimated by ASTRAL-III analysis of the dataset having 0.30 of occupancy and including 1,634 UCE loci (**analysis number 11**, see Table 1) coded as nucleotides using SWSC-EN partitioning scheme. Local posterior probabilities are shown in front to each node.

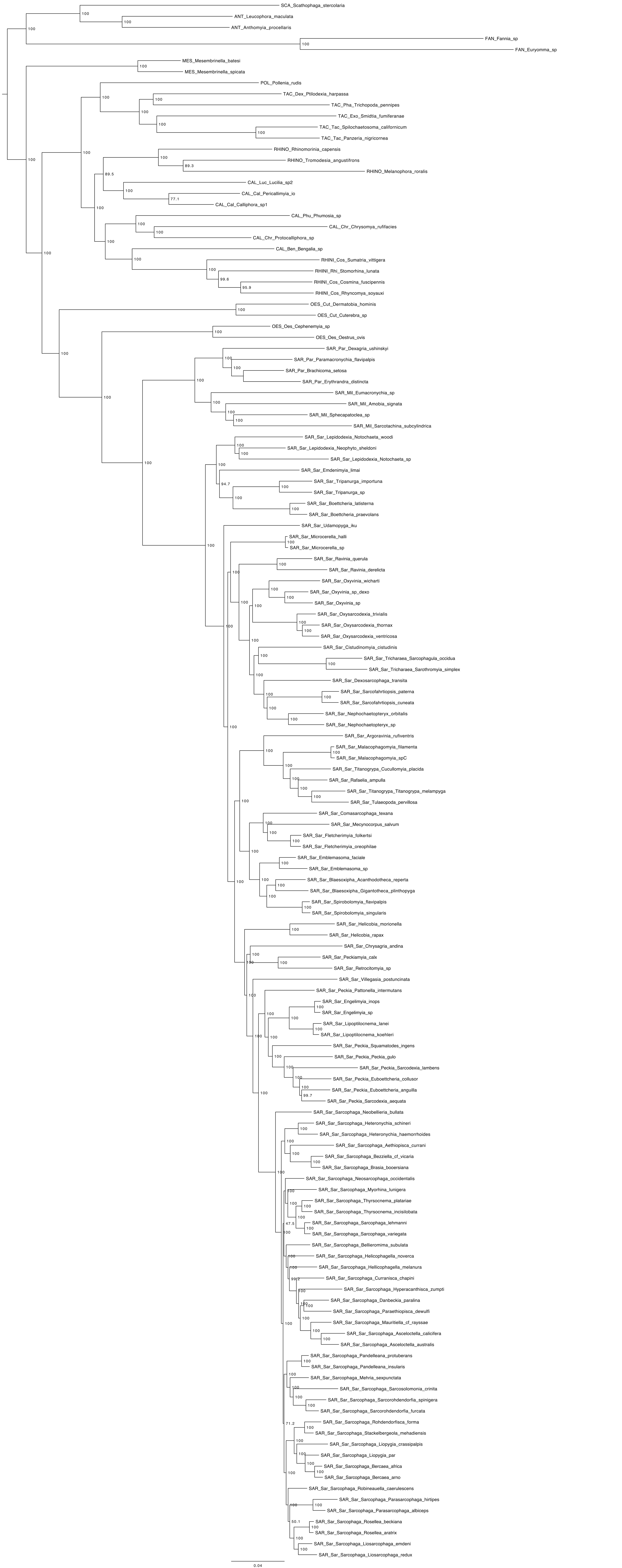

2.11. Phylogeny of Sarcophagidae. Species tree (topology A) estimated by ASTRAL-III analysis of the dataset having 0.60 of occupancy and including 1,271 UCE loci (**analysis number 12**, see Table 1) coded as nucleotides using SWSC-EN partitioning scheme. Local posterior probabilities are shown in front to each node.

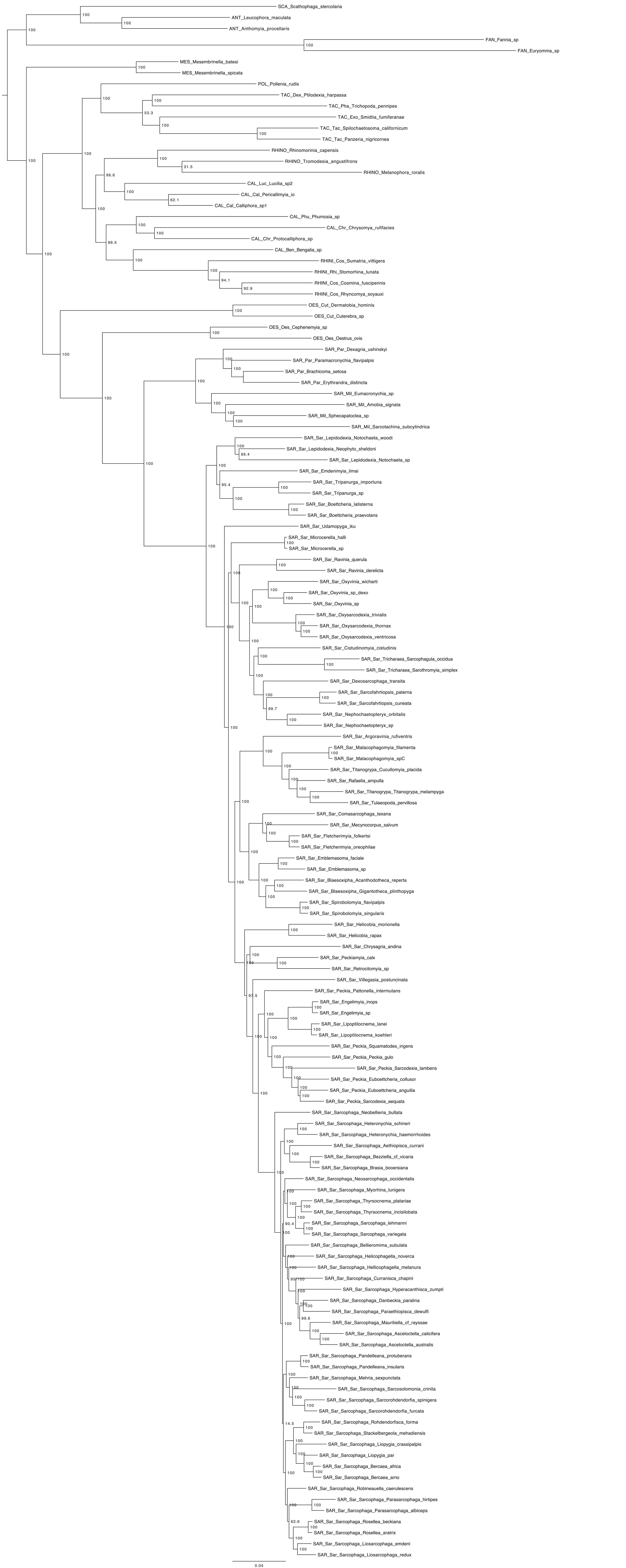

2.12. Phylogeny of Sarcophagidae. Species tree (topology int.) estimated by ASTRAL-III analysis of the dataset having 0.75 of occupancy and including 288 UCE loci (**analysis number 13**, see Table 1) coded as nucleotides using SWSC-EN partitioning scheme. Local posterior probabilities are shown in front to each node.

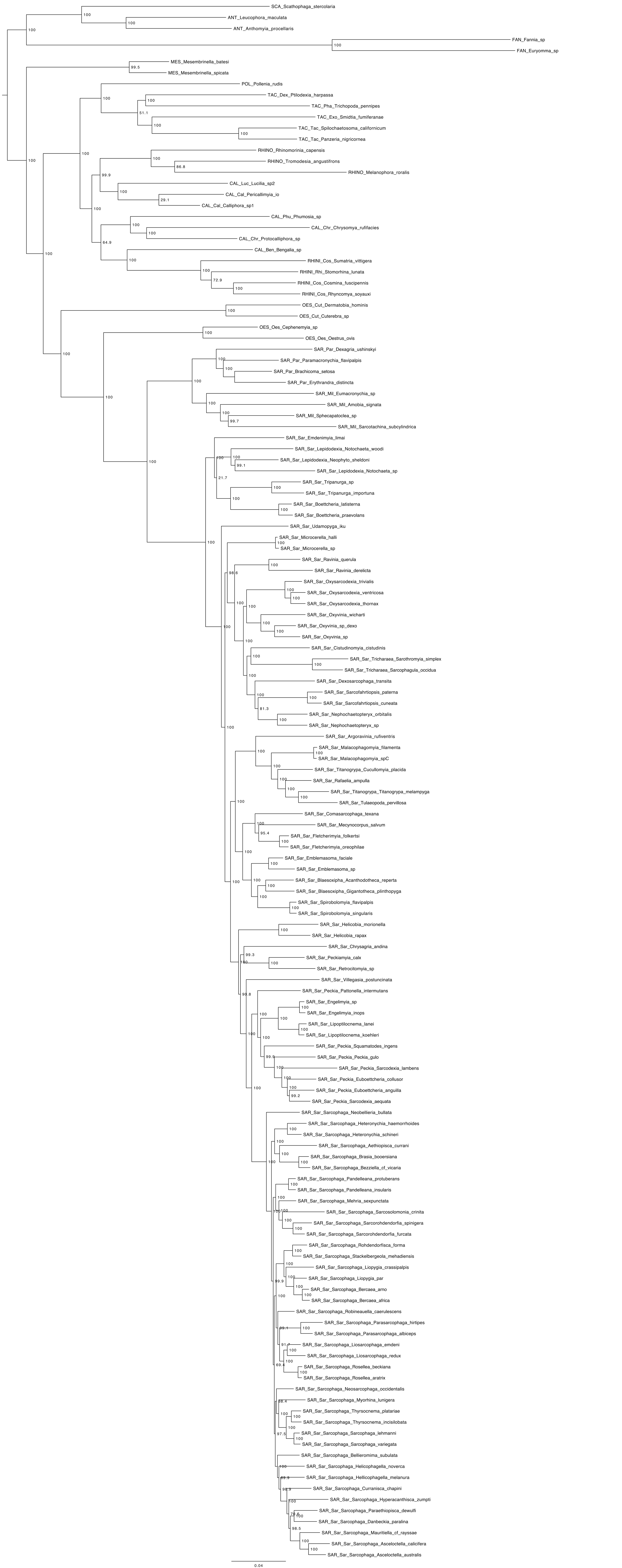

2.13. Phylogeny of Sarcophagidae. Species tree (topology A) estimated by ASTRAL-III analysis of the dataset having 0.10 of occupancy and including 2,018 UCE loci (**analysis number 14**, see Table 1) coded as amino acids using SWSC-EN partitioning scheme. Local posterior probabilities are shown in front to each node.

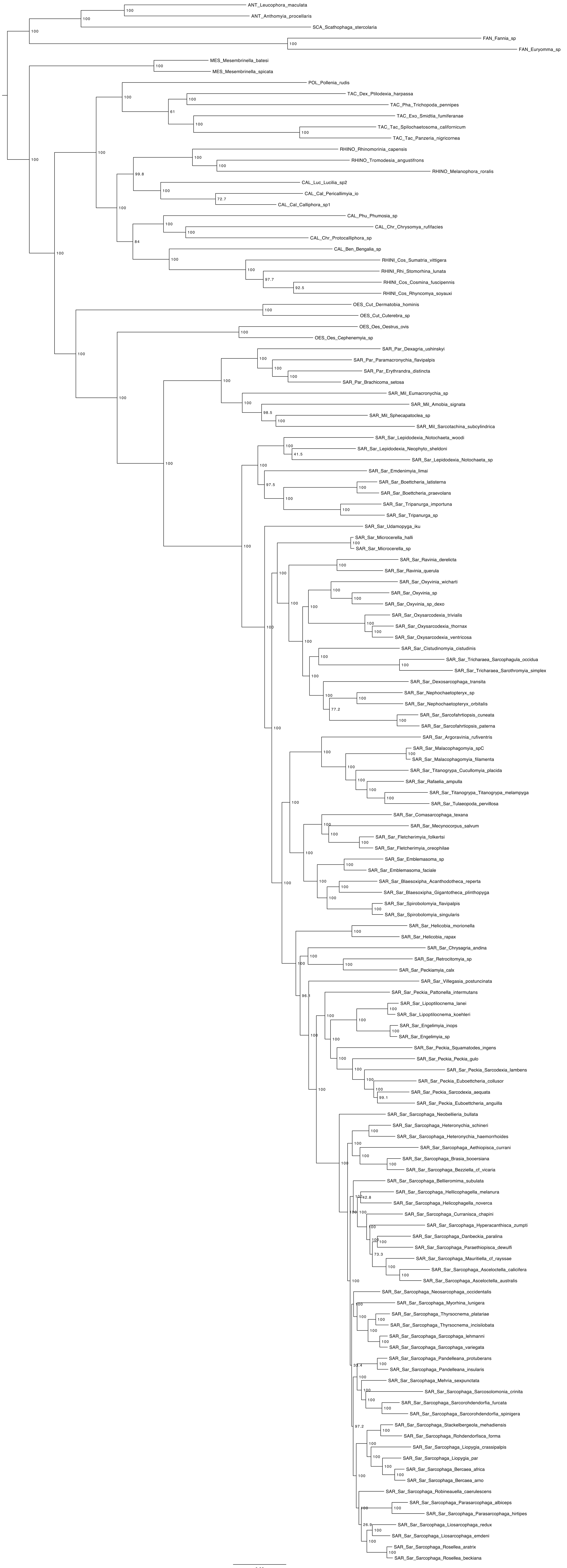

2.14. Phylogeny of Sarcophagidae. Species tree (topology A) estimated by ASTRAL-III analysis of the dataset having 0.30 of occupancy and including 1,634 UCE loci (**analysis number 15**, see Table 1) coded as amino acids using SWSC-EN partitioning scheme. Local posterior probabilities are shown in front to each node.

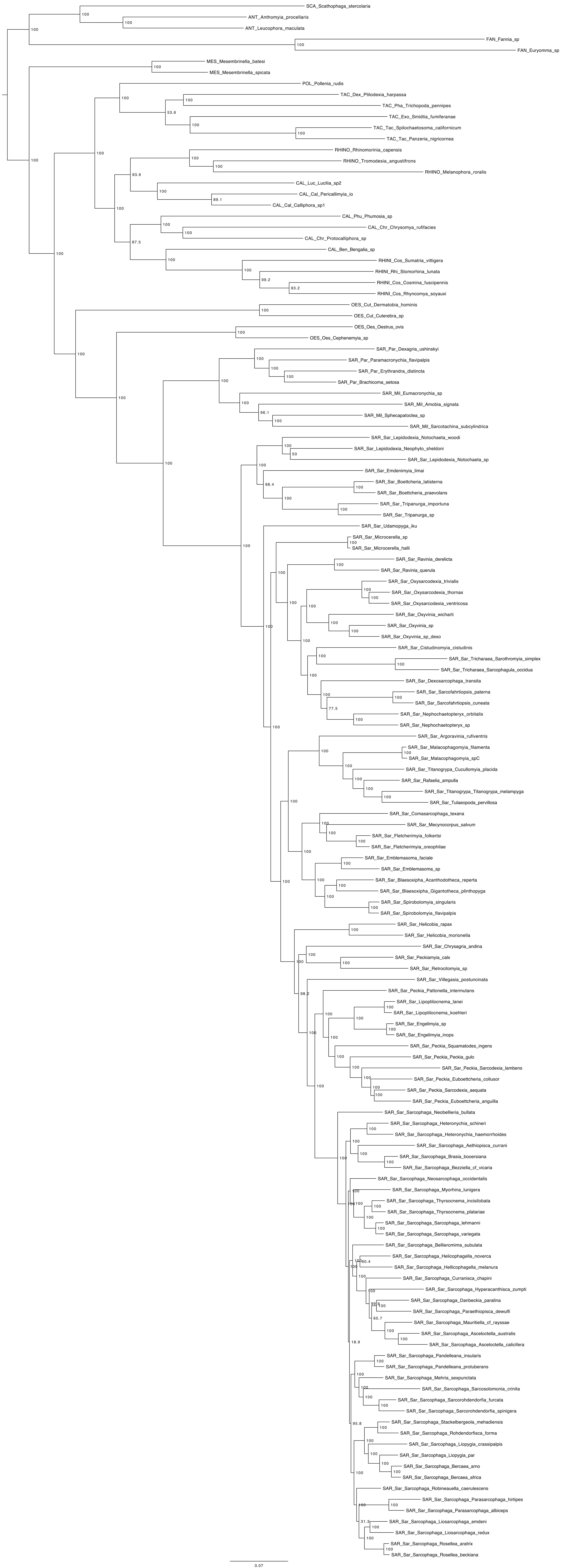

2.15. Phylogeny of Sarcophagidae. Species tree (topology A) estimated by ASTRAL-III analysis of the dataset having 0.60 of occupancy and including 1,271 UCE loci (**analysis number 16**, see Table 1) coded as amino acids using SWSC-EN partitioning scheme. Local posterior probabilities are shown in front to each node.

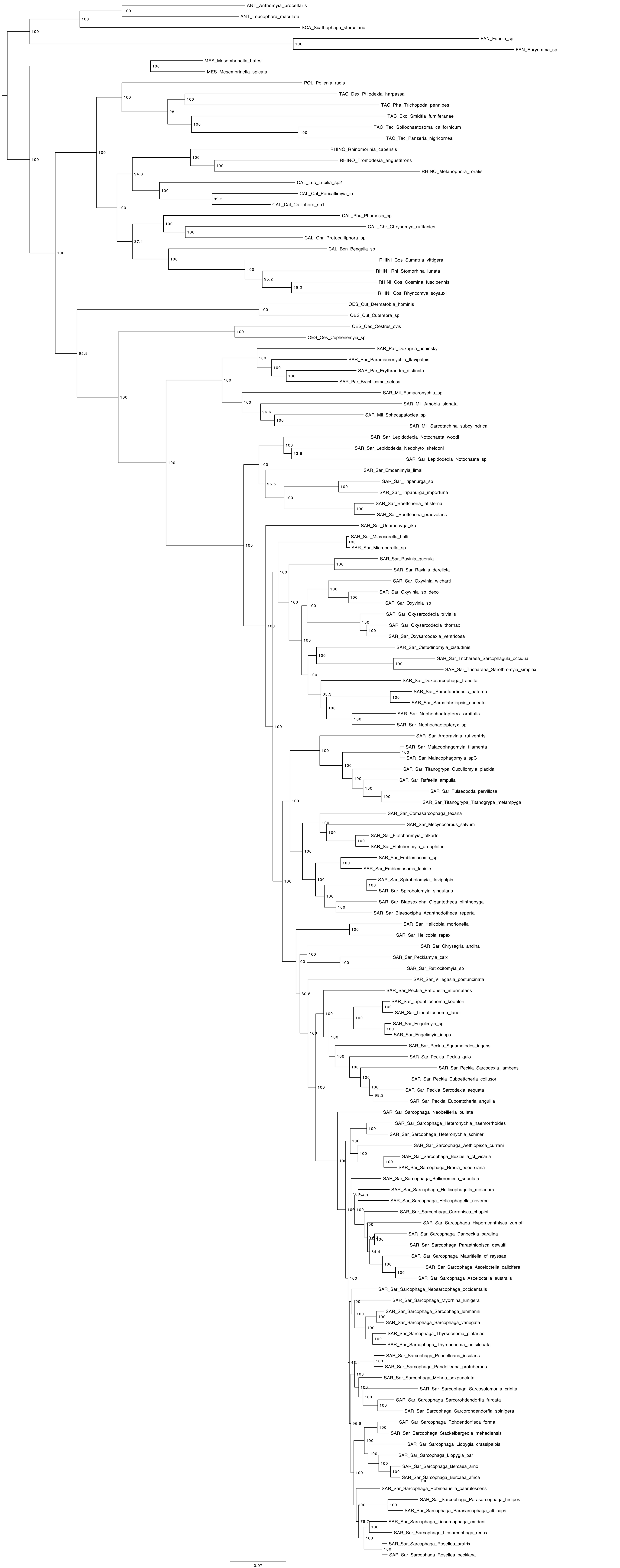

2.16. Phylogeny of Sarcophagidae. Species tree (topology int.) estimated by ASTRAL-III analysis of the dataset having 0.75 of occupancy and including 288 UCE loci (**analysis number 17**, see Table 1) coded as nucleotides using SWSC-EN partitioning scheme. Local posterior probabilities are shown in front to each node.

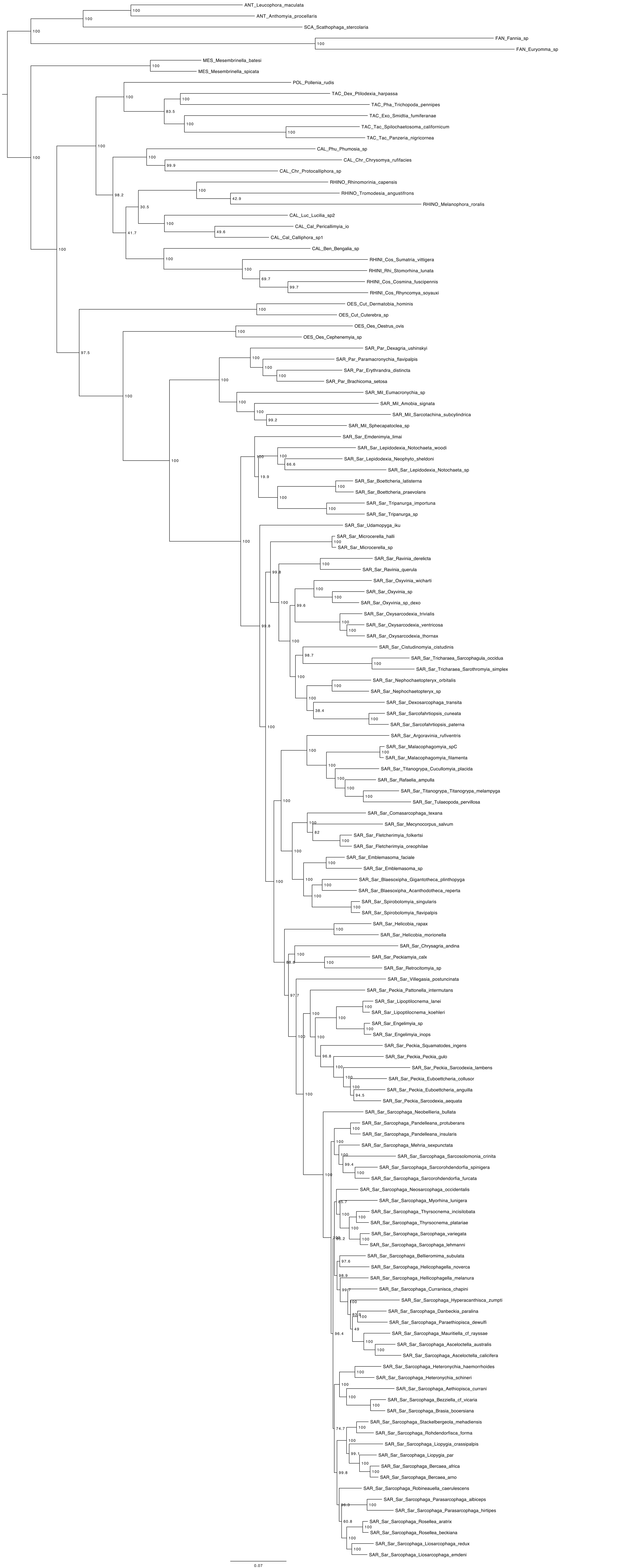

Supplement: Supplementary file 2 — Additional file 2. Phylogenetic relationships for Sarcophagidae and 10 other Calyptratae fly families inferred from 17 datasets having varying loci occupancy and coded as nucleotides and amino acids using a concatenated ML approach as well as by reconstructing a species tree estimated from UCE gene trees. Datasets are described in Table 1. [file 12862_2021_1797_MOESM2_ESM.pdf]
