# Supplementary material for: Museomics and phylogenomics with protein-encoding ultraconserved elements illuminate the evolution of life history and phallic morphology of flesh flies (Diptera: Sarcophagidae)
Source: BMC Ecol Evol. 2021 Apr 28;21:70. doi: 10.1186/s12862-021-01797-7 (PMC8082969; doi:10.1186/s12862-021-01797-7)
Supplement: Supplementary file 3 — Additional file 3. Comparison of models used for ancestral character state reconstructions. Models compared are ‘equal rates’ (ER, one transition rate) and ‘all rates different’ (ARD). Significantly better values are in bold. Characters analysed are: (1) larval food resource, (2) larval feeding habits, (3) abdominal ST5, shape of posterior margin, (4) cercal prong, outline of dorsal surface in lateral view, (5) phallus, connection between basi- and distiphallus, (6) phallus, connection between basi- and distiphallus, shape, (7) harpes, (8) vesica, (9) phallotrema, configuration, (10) phallotrema, position with regard to phallic tube, (11) acrophallic levers, (12) styli, number, (13) capitis, (14) median process, and (15) juxta. [file 12862_2021_1797_MOESM3_ESM.pdf]

Additional file 3. Comparison of models used for ancestral character state reconstructions. Models compared are 'equal rates' (ER, one transition rate) and 'all rates different' (ARD). Significantly better values are in bold. Characters analysed are: (1) larval food resource, (2) larval feeding habits, (3) abdominal ST5, shape of posterior margin, (4) cercal prong, outline of dorsal surface in lateral view, (5) phallus, connection between basi- and distiphallus, (6) phallus, connection between basi- and distiphallus, shape, (7) harpes, (8) vesica, (9) phallotrema, configuration, (10) phallotrema, position with regard to phallic tube, (11) acrophallic levers, (12) styli, number, (13) capitis, (14) median process and (15) juxta.

Character	Model	-lnL	AIC	AICc	Difference
1	**ARD**	-72.80864	169.6173	**172.8009**	
	ER	-89.30511	180.6102	180.6469	7.8
2	ARD	-83.9557	207.9114	217.2447	
	**ER**	-101.5164	205.0328	**205.0695**	12.2
3	ARD	-11.59651	27.19302	27.30413	
	**ER**	-11.60953	25.21905	25.25575	2.0
4	**ARD**	-73.33492	170.6698	**173.8535**	
	ER	-94.77865	191.5573	191.594	17.7
5	ARD	-11.86001	27.72003	27.83114	
	**ER**	-11.86126	25.72252	**25.75921**	2.1
6	ARD	-16.79748	45.59495	46.40264	
	**ER**	-17.70037	37.40073	**37.43743**	9.0
7	**ARD**	-30.51638	65.03277	65.14388	
	ER	-31.82506	65.65012	65.68682	0.5
8	ARD	-23.31111	50.62223	50.73334	
	**ER**	-23.33626	48.67252	48.70922	2.0
9	ARD	-6.65729	17.31458	17.42569	
	**ER**	-6.865953	15.73191	15.7686	1.7
10	ARD	-5.517948	15.0359	15.14701	
	**ER**	-5.896321	13.79264	13.82934	1.3
11	ARD	-6.924375	17.84875	17.95986	
	**ER**	-7.120624	16.24125	16.27794	1.7
12	ARD	-18.77524	49.55049	50.35818	
	**ER**	-19.87109	41.74219	**41.77889**	8.6
13	**ARD**	-10.09124	24.18249	24.2936	
	ER	-10.26919	22.53838	22.57508	1.7
14	ARD	-12.83728	29.67457	29.78568	
	**ER**	-13.10558	28.21117	28.24786	1.5
15	ARD	-5.517948	15.0359	15.14701	
	**ER**	-5.896321	13.79264	13.82934	1.3
