# Supplementary material for: Museomics and phylogenomics with protein-encoding ultraconserved elements illuminate the evolution of life history and phallic morphology of flesh flies (Diptera: Sarcophagidae)
Source: BMC Ecol Evol. 2021 Apr 28;21:70. doi: 10.1186/s12862-021-01797-7 (PMC8082969; doi:10.1186/s12862-021-01797-7)
Supplement: Supplementary file 15 — Additional file 15. Species richness matrix used for diversification analyses. This table lists the assigned clade for each species, the number of species sampled, and the number of nominal species described. From these cladespecific values, sampling fractions were calculated and used for BAMM analyses. [file 12862_2021_1797_MOESM15_ESM.pdf]

Additional file 15. Species richness matrix used for diversification analyses. This table lists the assigned clade for each species, the number of species sampled, and the number of nominal species described. From these cladespecific values, sampling fractions were calculated and used for BAMM analyses.

speciesName	cladeName	species_sampled	species_described	samplingFraction
Amobia_signata	amobia	1	14	0.071
Eumacronychia_sp	eumacr	1	22	0.045
Sarcotachina_sp	sarcot	2	3	0.667
Sarcotachina_subcylindrica	sarcot	2	3	0.667
Sphecapatoclea_sp	spheca	1	15	0.067
Brachicoma_setosa	brachi	1	8	0.125
Dexagria_ushinskyi	dexagr	1	3	0.333
Erythrandra_distincta	erythr	1	2	0.500
Paramacronychia_flavipalpis	parama	1	3	0.333
Argoravinia_rufiventris	argora	1	8	0.125
Blaesoxipha_Acanthodotheca_reperta	blaeso	2	254	0.008
Blaesoxipha_Gigantotheca_plinthopyga	blaeso	2	254	0.008
Boettcheria_latisterna	boettc	2	32	0.063
Boettcheria_praevolans	boettc	2	32	0.063
Chrysagria_andina	chrysa	1	3	0.333
Cistudinomyia_cistudinis	cistud	1	1	1.000
Comasarcophaga_texana	comasa	1	4	0.250
Dexosarcophaga_transita	dexosa	1	49	0.020
Emblemasoma_faciale	emblem	2	16	0.125
Emblemasoma_sp	emblem	2	16	0.125
Emdenimyia_limai	emdeni	1	11	0.091
Engelimyia_inops	engeli	2	4	0.500
Engelimyia_sp	engeli	2	4	0.500
Fletcherimyia_folkertsi	fletch	2	8	0.250
Fletcherimyia_oreophilae	fletch	2	8	0.250
Helicobia_morionella	helico	2	36	0.056
Helicobia_rapax	helico	2	36	0.056
Lepidodexia_Neophyto_sheldoni	lepido	3	186	0.016
Lepidodexia_Notochaeta_sp	lepido	3	186	0.016
Lepidodexia_Notochaeta_woodi	lepido	3	186	0.016
Lipoptilocnema_koehleri	lipopt	2	10	0.200
Lipoptilocnema_lanei	lipopt	2	10	0.200
Malacophagomyia_filamenta	malaco	2	3	0.667
Malacophagomyia_spC	malaco	2	3	0.667
Mecynocorpus_salvum	mecyno	1	1	1.000
Microcerella_sp	microc	2	80	0.025
Microcerella_halli	microc	2	80	0.025
Nephochaetopteryx_orbitalis	nephoc	2	39	0.051
Nephochaetopteryx_sp	nephoc	2	39	0.051
Oxysarcodexia_thornax	oxysar	3	96	0.031
Oxysarcodexia_trivialis	oxysar	3	96	0.031
Oxysarcodexia_ventricosa	oxysar	3	96	0.031
Oxyvinia_sp	oxyvin	3	10	0.300
Oxyvinia_sp_dexo	oxyvin	3	10	0.300
Oxyvinia_wicharti	oxyvin	3	10	0.300
Peckia_Euboettcheria_anguilla	peckia	7	75	0.093
Peckia_Euboettcheria_collusor	peckia	7	75	0.093
Peckia_Pattonella_intermutans	peckia	7	75	0.093
Peckia_Peckia_gulo	peckia	7	75	0.093
Peckia_Sarcodexia_aequata	peckia	7	75	0.093
Peckia_Sarcodexia_lambens	peckia	7	75	0.093
Peckia_Squamatodes_ingens	peckia	7	75	0.093
Peckiamyia_calx	peckim	1	6	0.167
Rafaelia_ampulla	rafael	1	8	0.125
Ravinia_derelicta	ravini	2	34	0.059
Ravinia_querula	ravini	2	34	0.059
Retrocitomyia_sp	retroc	1	11	0.091
Sarcofahrtiopsis_cuneata	sarcof	2	15	0.133
Sarcofahrtiopsis_paterna	sarcof	2	15	0.133
Sarcophaga_Aethiopisca_currani	sarcop	41	874	0.047
Sarcophaga_Asceloctella_australis	sarcop	41	874	0.047
Sarcophaga_Asceloctella_calicifera	sarcop	41	874	0.047
Sarcophaga_Bellieromima_subulata	sarcop	41	874	0.047
Sarcophaga_Bercaea_africa	sarcop	41	874	0.047
Sarcophaga_Bercaea_arno	sarcop	41	874	0.047
Sarcophaga_Bezziella_cfvicaria	sarcop	41	874	0.047
Sarcophaga_Brasia_booersiana	sarcop	41	874	0.047

Sarcophaga_Curranisca_chapini	sarcop	41	874	0.047
Sarcophaga_Danbeckia_paralina	sarcop	41	874	0.047
Sarcophaga_Hellicophagella_melanura	sarcop	41	874	0.047
Sarcophaga_Helicophagella_noverca	sarcop	41	874	0.047
Sarcophaga_Heteronychia_haemorrhoides	sarcop	41	874	0.047
Sarcophaga_Heteronychia_schineri	sarcop	41	874	0.047
Sarcophaga_Hyperacanthisca_zumpti	sarcop	41	874	0.047
Sarcophaga_Liopygia_crassipalpis	sarcop	41	874	0.047
Sarcophaga_Liopygia_par	sarcop	41	874	0.047
Sarcophaga_Liosarcophaga_emdeni	sarcop	41	874	0.047
Sarcophaga_Liosarcophaga_redux	sarcop	41	874	0.047
Sarcophaga_Mauritiella_cfrayssae	sarcop	41	874	0.047
Sarcophaga_Mehria_sexpunctata	sarcop	41	874	0.047
Sarcophaga_Myorhina_lunigera	sarcop	41	874	0.047
Sarcophaga_Neobellieria_bullata	sarcop	41	874	0.047
Sarcophaga_Neosarcophaga_occidentalis	sarcop	41	874	0.047
Sarcophaga_Pandelleana_insularis	sarcop	41	874	0.047
Sarcophaga_Pandelleana_protuberans	sarcop	41	874	0.047
Sarcophaga_Paraethiopisca_dewulfi	sarcop	41	874	0.047
Sarcophaga_Parasarcophaga_albiceps	sarcop	41	874	0.047
Sarcophaga_Parasarcophaga_hirtipes	sarcop	41	874	0.047
Sarcophaga_Robineauella_caerulescens	sarcop	41	874	0.047
Sarcophaga_Rohdendorfisca_forma	sarcop	41	874	0.047
Sarcophaga_Rosellea_aratrix	sarcop	41	874	0.047
Sarcophaga_Rosellea_beckiana	sarcop	41	874	0.047
Sarcophaga_Sarcophaga_lehmanni	sarcop	41	874	0.047
Sarcophaga_Sarcophaga_variegata	sarcop	41	874	0.047
Sarcophaga_Sarcorohdendorfia_furcata	sarcop	41	874	0.047
Sarcophaga_Sarcorohdendorfia_spinigera	sarcop	41	874	0.047
Sarcophaga_Sarcosolomonia_crinita	sarcop	41	874	0.047
Sarcophaga_Stackelbergeola_mehadiensis	sarcop	41	874	0.047
Sarcophaga_Thyrsocnema_incisilobata	sarcop	41	874	0.047
Sarcophaga_Thyrsocnema_platariae	sarcop	41	874	0.047
Spirobolomyia_flavipalpis	spirob	2	5	0.400
Spirobolomyia_singularis	spirob	2	5	0.400
Titanogrypa_Cucullomyia_placida	titano	2	18	0.111
Titanogrypa_Titanogrypa_melampyga	titano	2	18	0.111
Tricharaea_Sarcophagula_occidua	tricha	2	15	0.133
Tricharaea_Sarothromyia_simplex	tricha	2	15	0.133
Tripanurga_importuna	tripan	2	13	0.154
Tripanurga_sp	tripan	2	13	0.154
Tulaeopoda_pervillosa	tulaeo	1	3	0.333
Udamopyga_iku	udamop	1	22	0.045
Villegasia_postuncinata	villeg	1	3	0.333
