# Supplementary material for: Museomics and phylogenomics with protein-encoding ultraconserved elements illuminate the evolution of life history and phallic morphology of flesh flies (Diptera: Sarcophagidae)
Source: BMC Ecol Evol. 2021 Apr 28;21:70. doi: 10.1186/s12862-021-01797-7 (PMC8082969; doi:10.1186/s12862-021-01797-7)
Supplement: Supplementary file 16 — Additional file 16. Dataset of biological characters including ingroup and outgroup. Characters: 1) Larval food resource: (0) invertebrates; (1) vertebrates; (2) feces; (3) plants; (4) ? (unknown); 2) larval feeding habits: (0) kleptoparasitism; (1) sarcosaprophagy; (2) predation; (3) coprophagy; (4) phytophagy; (5) ? (unknown). [file 12862_2021_1797_MOESM16_ESM.pdf]

Additional file 16. Dataset of biological characters including ingroup and outgroup. Characters: 1) Larval food resource: (0) invertebrates; (1) vertebrates; (2) feces; (3) plants; (4) ? (unknown); 2) larval feeding habits: (0) kleptoparasitism; (1) sarcosaprophagy; (2) predation; (3) coprophagy; (4) phytophagy; (5) ? (unknown).

Taxa	1	2
FAN Euryomma sp	0&1&2&3	1&3&4
FAN Fannia sp	3	1&4
ANT Anthomyia procellaris	3	4
ANT Leucophora maculata	3	4
SCA Scatophaga stercolaria	2	3
MES Mesembrinella batesi	?	?
MES Mesembrinella spicata	?	?
POL Pollenia rudis	0	2
TAC Dex Ptilodexia harpassa	?	?
TAC Exo Smidtia fumiferanae	0	2
TAC Pha Trichopoda pennipes	0	2
TAC Tac Panzeria nigricornea	0	2
TAC Tac Spilochaetosoma californicum	?	?
RHINO Melanophora roralis	0	2
RHINO Rhinomorinia capensis	0	2
RHINO Tromodesia angustifrons	0	2
CAL Luc Lucilia sp2	1	1
CAL Cal Pericallimyia io	1	1
CAL Cal Calliphora sp1	1	1
CAL Phu Phumosia sp	0	1&2
CAL Chr Chrysomya rufifacies	1&2	1
CAL Chr Protocalliphora sp	1	2
CAL Ben Bengalia sp	0	0
RHINI Cos Rhyncomya soyauxi	0	0
RHINI Cos Cosmina fuscipennis	0	0
RHINI Cos Sumatria vittigera	0	0
RHINI Rhi Stomorhina lunata	0	0
OES Cut Cuterebra sp	1	2
OES Cut Dermatobia hominis	1	2
OES Oes Oestrus ovis	1	2
OES Oes Cephenemyia sp	1	2
Amobia_signata	0	0
Eumacronychia_sp	1	1&2
Sarcotachina_sp	1	1
Sarcotachina_subcylindrica	1	1
Sphecapatoclea_sp	0	2
Brachicoma_setosa	0	2
Dexagria_ushinskyi	?	?
Erythrandra_distincta	0	2
Paramacronychia_flavipalpis	0	1
Argoravinia_rufiventris	1	1
Blaesoxipha_Acanthodotheca_reperta	0	2
Blaesoxipha_Gigantotheca_plinthopyga	0&1	1
Boettcheria_latisterna	0	2
Boettcheria_praevolans	0	2
Chrysagria_andina	0	2
Cistudinomyia_cistudinis	1	2
Comasarcophaga_texana	0	1&2
Dexosarcophaga_transita	0	0&1
Emblemasoma_faciale	0	2
Emblemasoma_sp	0	2
Emdenimyia_limai	0	2
Engelimyia_inops	1	1&3
Engelimyia_sp	1	1
Fletcherimyia_folkertsi	0	0
Fletcherimyia_oreophilae	0	0
Helicobia_morionella	0	1&2
Helicobia_rapax	0	1&2
Lepidodexia_Neophyto_sheldoni	0	2
Lepidodexia_Notochaeta_sp	1	2
Lepidodexia_Notochaeta_woodi	1	2
Lipoptilocnema_koehleri	0	1
Lipoptilocnema_lanei	1	1
Malacophagomyia_filamenta	0	2
Malacophagomyia_spC	0	2
Mecynocorpus_salvum	0	2
Microcerella_sp	1	1

Microcerella_halli	1	1
Nephochaetopteryx_orbitalis	2	3
Nephochaetopteryx_sp	2	3
Oxysarcodexia_thornax	1&2	1&3
Oxysarcodexia_trivialis	2	3
Oxysarcodexia_ventricosa	2	3
Oxyvinia_sp	2	3
Oxyvinia_sp_dexo	2	3
Oxyvinia_wicharti	2	3
Peckia_Euboettcheria_anguilla	1	1
Peckia_Euboettcheria_collusor	1	1
Peckia_Pattonella_intermutans	1	1
Peckia_Peckia_gulo	0	1
Peckia_Sarcodexia_aequata	1	1
Peckia_Sarcodexia_lambens	0&1	1&2
Peckia_Squamatodes_ingens	1	1
Peckiamyia_calx	1	1
Rafaelia_ampulla	0	2
Ravinia_derelicta	0&2	2&3
Ravinia_querula	0&2	2&3
Retrocitomyia_sp	1	1
Sarcofahrtiopsis_cuneata	1&2	1&3
Sarcofahrtiopsis_paterna	1&2	1&3
Sarcophaga_Aethiopisca_currani	0&1&2	1&2&3
Sarcophaga_Asceloctella_australis	0	1
Sarcophaga_Asceloctella_calicifera	0	1
Sarcophaga_Bellieromima_subulata	0	1&2
Sarcophaga_Bercaea_africa	0	1&3
Sarcophaga_Bercaea_arno	0	3
Sarcophaga_Bezziella_cfvicaria	0	2
Sarcophaga_Brasia_booersiana	0	2
Sarcophaga_Curranisca_chapini	0&1&2	1&2&3
Sarcophaga_Danbeckia_paralina	?	?
Sarcophaga_Helicophagella_noverca	0&1&2	1&2&3
Sarcophaga_Hellicophagella_melanura	0&1	1&2
Sarcophaga_Heteronychia_haemorrhoides	0	2
Sarcophaga_Heteronychia_schineri	0	2
Sarcophaga_Hyperacanthisca_zumpti	?	?
Sarcophaga_Liopygia_crassipalpis	0&1	1&2
Sarcophaga_Liopygia_par	0	2
Sarcophaga_Liosarcophaga_emdeni	0	1&2
Sarcophaga_Liosarcophaga_redux	0&1	1&2
Sarcophaga_Mauritiella_cfrayssae	0	1&2
Sarcophaga_Mehria_sexpunctata	0	2
Sarcophaga_Myorhina_lunigera	0	2
Sarcophaga_Neobellieria_bullata	0&1	1&2
Sarcophaga_Neosarcophaga_occidentalis	?	?
Sarcophaga_Pandelleana_insularis	0	2
Sarcophaga_Pandelleana_protuberans	0	2
Sarcophaga_Paraethiopisca_dewulfi	?	?
Sarcophaga_Parasarcophaga_albiceps	0&1&2	1&2&3
Sarcophaga_Parasarcophaga_hirtipes	0&1&2	1&2&3
Sarcophaga_Robineauella_caerulescens	0	1&2
Sarcophaga_Rohdendorfisca_forma	0	2
Sarcophaga_Rosellea_aratrix	0	1&2
Sarcophaga_Rosellea_beckiana	0	1&2
Sarcophaga_Sarcophaga_lehmanni	0	2
Sarcophaga_Sarcophaga_variegata	0	2
Sarcophaga_Sarcorohdendorfia_furcata	1	1
Sarcophaga_Sarcorohdendorfia_spinigera	1	1
Sarcophaga_Sarcosolomonia_crinita	0	0&1
Sarcophaga_Stackelbergeola_mehadiensis	?	?
Sarcophaga_Thyrsocnema_incisilobata	0&2	1&2&3
Sarcophaga_Thyrsocnema_platariae	1&2	1&3
Spirobolomyia_flavipalpis	0	2
Spirobolomyia_singularis	0	2
Titanogrypa_Cucullomyia_placida	0	1&2
Titanogrypa_Titanogrypa_melampyga	0	1&2
Tricharaea_Sarcophagula_occidua	0&1&2	1&3
Tricharaea_Sarothromyia_simplex	0&1	1
Tripanurga_importuna	1	2
Tripanurga_sp	1	2
Tulaeopoda_pervillosa	0	2
Udamopyga_iku	0	1&2

Villegasia_postuncinata	0	1
