# Supplementary material for: Museomics and phylogenomics with protein-encoding ultraconserved elements illuminate the evolution of life history and phallic morphology of flesh flies (Diptera: Sarcophagidae)
Source: BMC Ecol Evol. 2021 Apr 28;21:70. doi: 10.1186/s12862-021-01797-7 (PMC8082969; doi:10.1186/s12862-021-01797-7)
Supplement: Supplementary file 17 — Additional file 17. Dataset of biological and morphological characters including ingroup only. Characters: 1) Larval food resource: (0) invertebrates; (1) vertebrates; (2) feces; (3) n/a (unknown); 2) larval feeding habits: (0) kleptoparasitism; (1) sarcosaprophagy; (2) predation; (3) coprophagy; (4) n/a (unknown); 3) abdominal ST5, shape of posterior margin: (0) straight or with a shallow concavity; (1) forming a cleft; 4) cercal prong, outline of dorsal surface in lateral view: (0) straight or almost straight; (1) swollen or curved uniformly; (2) with a proximal hump; (3) with a subapical saddle-shaped concavity followed by a hump; 5) phallus, connection between basi- and distiphallus: (0) continuous; (1) non-continuous; 6) phallus, connection between basi- and distiphallus, shape: (0) desclerotized band; (1) distinct hinge; (2) partially sclerotized; (3) fully sclerotized; 7) harpes: (0) absent; (1) present; 8) vesica: (0) absent; (1) present; 9) phallotrema, configuration: (0) not folded, forming one opening; (1) folded, forming three openings; 10) phallotrema, position with regard to phallic tube: (0) apical; (1) ventral; 11) acrophallic levers: (0) present; (1) absent; 12) styli, number: (0) one; (1) three; (2) two; 13) capitis: (0) present; (1) absent; 14) median process: (0) present; (1) absent or reduced; 15) juxta: (0) absent; (1) present. [file 12862_2021_1797_MOESM17_ESM.pdf]

Additional file 17. Dataset of biological and morphological characters including ingroup only. Characters: 1) Larval food resource: (0) invertebrates; (1) vertebrates; (2) feces; (3) n/a (unknown); 2) larval feeding habits: (0) kleptoparasitism; (1) sarcosaprophagy; (2) predation; (3) coprophagy; (4) n/a (unknown); 3) abdominal ST5, shape of posterior margin: (0) straight or with a shallow concavity; (1) forming a cleft; 4) cercal prong, outline of dorsal surface in lateral view: (0) straight or almost straight; (1) swollen or curved uniformly; (2) with a proximal hump; (3) with a subapical saddle-shaped concavity followed by a hump; 5) phallus, connection between basi- and distiphallus: (0) continuous; (1) non-continuous; 6) phallus, connection between basi- and distiphallus, shape: (0) desclerotized band; (1) distinct hinge; (2) partially sclerotized; (3) fully sclerotized; 7) harpes: (0) absent; (1) present; 8) vesica: (0) absent; (1) present; 9) phallotrema, configuration: (0) not folded, forming one opening; (1) folded, forming three openings; 10) phallotrema, position with regard to phallic tube: (0) apical; (1) ventral; 11) acrophallic levers: (0) present; (1) absent; 12) styli, number: (0) one; (1) three; (2) two; 13) capitis: (0) present; (1) absent; 14) median process: (0) present; (1) absent or reduced; 15) juxta: (0) absent; (1) present.

Taxa	1	2	3	4	5	6	7	8	9	10	11	12	13	14	15
Amobia_signata	0	0	0	0	0	3	0	0	0	0	1	0	1	0	0
Eumacronychia_sp	1	1&2	0	0	0	3	0	0	0	0	1	0	1	0	0
Sarcotachina_sp	1	1	0	0	0	3	0	0	0	0	1	0	1	0	0
Sarcotachina_subcylindrica	1	1	0	0	0	3	0	0	0	0	1	0	1	0	0
Sphecapatoclea_sp	0	2	0	0	0	3	0	0	0	0	1	0	1	0	0
Brachicoma_setosa	0	2	0	0	0	3	0	0	0	1	1	0	0	0	1
Dexagria_ushinskyi	n/a	n/a	0	0	0	3	0	0	0	1	1	0	0	0	1
Erythrandra_distincta	0	2	0	0	0	3	0	0	0	1	1	0	0	0	1
Paramacronychia_flavipalpis	0	1	0	0	0	3	0	0	0	1	1	0	0	0	1
Argoravinia_rufiventris	1	1	1	0	1	1	0	1	1	1	1	1	0	0	1
Blaesoxipha_Acanthodotheca_reperta	0	2	1	2	1	1	0	0	1	1	1	1	0	0	1
Blaesoxipha_Gigantotheca_plinthopyga	0&1	1	1	2	1	1	0	0	1	1	1	1	0	0	1
Boettcheria_latisterna	0	2	1	0	1	1	1	1	1	1	1	1	0	0	1
Boettcheria_praevolans	0	2	1	0	1	1	1	1	1	1	1	1	0	0	1
Chrysagria_andina	0	2	1	0	1	1	1	1	1	1	1	2	0	1	1
Cistudinomyia_cistudinis	1	2	0	0	0	3	0	1	1	1	0	1	0	0	1
Comasarcophaga_texana	0	1&2	1	2	1	1	0	1	1	1	1	1	0	0	1
Dexosarcophaga_transita	0	0&1	0	0	0	3	0	1	1	1	0	1	0	0	1
Emblemasoma_faciale	0	2	1	0	1	1	1	1	1	1	1	1	0	0	1
Emblemasoma_sp	0	2	1	0	1	1	1	1	1	1	1	1	0	0	1
Emdenimyia_limai	0	2	1	0	1	1	0	0	1	1	1	1	0	0	1
Engelimyia_inops	1	1&3	1	3	1	1	0	1	1	1	1	2	0	1	1
Engelimyia_sp	1	1	1	3	1	1	0	1	1	1	1	2	0	1	1
Fletcherimyia_folkertsi	0	0	1	2	1	1	0	1	1	1	1	1	0	0	1
Fletcherimyia_oreophilae	0	0	1	2	1	1	0	1	1	1	1	1	0	0	1
Helicobia_morionella	0	1&2	1	0	1	1	1	1	1	1	1	2	0	1	1
Helicobia_rapax	0	1&2	1	0	1	1	1	1	1	1	1	2	0	1	1
Lepidodexia_Neophyto_sheldoni	0	2	1	0	1	1	1	1	1	1	1	1	0	0	1
Lepidodexia_Notochaeta_sp	1	2	1	0	1	1	1	1	1	1	1	1	0	0	1
Lepidodexia_Notochaeta_woodi	1	2	1	0	1	1	1	1	1	1	1	1	0	0	1
Lipoptilocnema_koehleri	0	1	1	3	1	1	1	1	1	1	1	2	0	1	1
Lipoptilocnema_lanei	1	1	1	3	1	1	1	1	1	1	1	2	0	1	1
Malacophagomyia_filamenta	0	2	1	0	1	1	0	1	1	1	1	1	0	0	1
Malacophagomyia_spC	0	2	1	0	1	1	0	1	1	1	1	1	0	0	1
Mecynocorpus_salvum	0	2	1	2	1	1	0	0	1	1	1	1	0	0	1
Microcerella_sp	1	1	1	0	1	2	1	1	1	1	1	1	0	0	1
Microcerella_halli	1	1	1	0	1	2	1	1	1	1	1	1	0	0	1
Nephochaetopteryx_orbitalis	2	3	0	0	0	3	0	1	1	1	0	1	0	0	1
Nephochaetopteryx_sp	2	3	0	0	0	3	0	1	1	1	0	1	0	0	1
Oxysarcodexia_thornax	1&2	1&3	0	0	0	3	0	1	1	1	0	1	0	0	1
Oxysarcodexia_trivialis	2	3	0	0	0	3	0	1	1	1	0	1	0	0	1
Oxysarcodexia_ventricosa	2	3	0	0	0	3	0	1	1	1	0	1	0	0	1
Oxyvinia_sp	2	3	0	0	0	3	0	1	1	1	0	1	0	0	1
Oxyvinia_sp_dexo	2	3	0	0	0	3	0	1	1	1	0	1	0	0	1
Oxyvinia_wicharti	2	3	0	0	0	3	0	1	1	1	0	1	0	0	1
Peckia_Euboettcheria_anguilla	1	1	1	3	1	1	0	1	1	1	1	2	1	1	1
Peckia_Euboettcheria_collusor	1	1	1	3	1	1	0	1	1	1	1	2	1	1	1
Peckia_Pattonella_intermutans	1	1	1	1	1	1	0	1	1	1	1	2	0	1	1
Peckia_Peckia_gulo	0	1	1	3	1	1	0	1	1	1	1	2	1	1	1
Peckia_Sarcodexia_aequata	1	1	1	3	1	1	0	1	1	1	1	2	1	1	1
Peckia_Sarcodexia_lambens	0&1	1&2	1	3	1	1	0	1	1	1	1	2	1	1	1
Peckia_Squamatodes_ingens	1	1	1	3	1	1	0	0	1	1	1	2	1	1	1
Peckiamyia_calx	1	1	1	0	1	1	0	1	1	1	1	1	0	0	1
Rafaelia_ampulla	0	2	1	0	1	1	0	1	1	1	1	1	0	0	1
Ravinia_derelicta	0&2	2&3	1	0	0	3	0	1	1	1	0	1	0	0	1
Ravinia_querula	0&2	2&3	1	0	0	3	0	1	1	1	0	1	0	0	1
Retrocitomyia_sp	1	1	1	0	1	1	0	1	1	1	1	1	0	0	1
Sarcofahrtiopsis_cuneata	1&2	1&3	0	0	0	3	0	1	1	1	0	1	0	0	1
Sarcofahrtiopsis_paterna	1&2	1&3	0	0	0	3	0	1	1	1	0	1	0	0	1
Sarcophaga_Aethiopisca_currani	0&1&2	1&2&3	1	3	1	1	1	1	1	1	1	2	0	1	1
Sarcophaga_Asceloctella_australis	0	1	1	0	1	1	1	1	1	1	1	2	0	1	1
Sarcophaga_Asceloctella_calicifera	0	1	1	0	1	1	1	1	1	1	1	2	0	1	1
Sarcophaga_Bellieromima_subulata	0	1&2	1	1	1	1	1	1	1	1	1	2	0	1	1
Sarcophaga_Bercaea_africa	0	1&3	1	3	1	1	1	1	1	1	1	2	0	1	1
Sarcophaga_Bercaea_arno	0	3	1	3	1	1	1	1	1	1	1	2	0	1	1
Sarcophaga_Bezziella_cfvicaria	0	2	1	0	1	1	1	1	1	1	1	2	0	1	1
Sarcophaga_Brasia_booersiana	0	2	1	0	1	1	1	1	1	1	1	2	0	1	1
Sarcophaga_Curranisca_chapini	0&1&2	1&2&3	1	1	1	1	1	1	1	1	1	2	0	1	1

Sarcophaga_Danbeckia_paralina	n/a	n/a	1	1	1	1	1	1	1	1	1	2	0	1	1
Sarcophaga_Helicophagella_noverca	0&1&2	1&2&3	1	0	1	1	1	1	1	1	1	2	0	1	1
Sarcophaga_Hellicophagella_melanura	0&1	1&2	1	0	1	1	1	1	1	1	1	2	0	1	1
Sarcophaga_Heteronychia_haemorrhoides	0	2	1	3	1	1	1	1	1	1	1	2	0	1	1
Sarcophaga_Heteronychia_schineri	0	2	1	3	1	1	1	1	1	1	1	2	0	1	1
Sarcophaga_Hyperacanthisca_zumpti	n/a	n/a	1	0	1	1	1	1	1	1	1	2	0	1	1
Sarcophaga_Liopygia_crassipalpis	0&1	1&2	1	3	1	1	1	1	1	1	1	2	0	1	1
Sarcophaga_Liopygia_par	0	2	1	3	1	1	1	1	1	1	1	2	0	1	1
Sarcophaga_Liosarcophaga_emdeni	0	1&2	1	0	1	1	1	1	1	1	1	2	0	1	1
Sarcophaga_Liosarcophaga_redux	0&1	1&2	1	3	1	1	1	1	1	1	1	2	0	1	1
Sarcophaga_Mauritiella_cfrayssae	0	1&2	1	0	1	1	1	1	1	1	1	2	0	1	1
Sarcophaga_Mehria_sexpunctata	0	2	1	0	1	1	1	1	1	1	1	2	0	1	1
Sarcophaga_Myorhina_lunigera	0	2	1	0	1	1	1	1	1	1	1	2	0	1	1
Sarcophaga_Neobellieria_bullata	0&1	1&2	1	3	1	1	1	1	1	1	1	2	0	1	1
Sarcophaga_Neosarcophaga_occidentalis	n/a	n/a	1	1	1	1	1	1	1	1	1	2	0	1	1
Sarcophaga_Pandelleana_insularis	0	2	1	0	1	1	1	1	1	1	1	2	0	1	1
Sarcophaga_Pandelleana_protuberans	0	2	1	0	1	1	1	1	1	1	1	2	0	1	1
Sarcophaga_Paraethiopisca_dewulfi	n/a	n/a	1	0	1	1	1	1	1	1	1	2	0	1	1
Sarcophaga_Parasarcophaga_albiceps	0&1&2	1&2&3	1	3	1	1	1	1	1	1	1	2	0	1	1
Sarcophaga_Parasarcophaga_hirtipes	0&1&2	1&2&3	1	0	1	1	1	1	1	1	1	2	0	1	1
Sarcophaga_Robineauella_caerulescens	0	1&2	1	1	1	1	1	1	1	1	1	2	0	1	1
Sarcophaga_Rohdendorfisca_forma	0	2	1	0	1	1	1	1	1	1	1	2	0	1	1
Sarcophaga_Rosellea_aratrix	0	1&2	1	1	1	1	1	1	1	1	1	2	0	1	1
Sarcophaga_Rosellea_beckiana	0	1&2	1	1	1	1	1	1	1	1	1	2	0	1	1
Sarcophaga_Sarcophaga_lehmanni	0	2	1	0	1	1	1	1	1	1	1	2	0	1	1
Sarcophaga_Sarcophaga_variegata	0	2	1	0	1	1	1	1	1	1	1	2	0	1	1
Sarcophaga_Sarcorohdendorfia_furcata	1	1	1	3	1	1	1	1	1	1	1	2	0	1	1
Sarcophaga_Sarcorohdendorfia_spinigera	1	1	1	3	1	1	1	1	1	1	1	2	0	1	1
Sarcophaga_Sarcosolomonia_crinita	0	0&1	1	1	1	1	1	1	1	1	1	2	0	1	1
Sarcophaga_Stackelbergeola_mehadiensis	n/a	n/a	1	1	1	1	1	1	1	1	1	2	0	1	1
Sarcophaga_Thyrsocnema_incisilobata	0&2	1&2&3	1	0	1	1	1	1	1	1	1	2	0	1	1
Sarcophaga_Thyrsocnema_platariae	1&2	1&3	1	0	1	1	1	1	1	1	1	2	0	1	1
Spirobolomyia_flavipalpis	0	2	1	2	1	1	0	1	1	1	1	1	0	0	1
Spirobolomyia_singularis	0	2	1	2	1	1	0	1	1	1	1	1	0	0	1
Titanogrypa_Cucullomyia_placida	0	1&2	1	0	1	1	0	1	1	1	1	1	0	0	1
Titanogrypa_Titanogrypa_melampyga	0	1&2	1	0	1	1	0	1	1	1	1	1	0	0	1
Tricharaea_Sarcophagula_occidua	0&1&2	1&3	0	0	0	3	0	1	1	1	0	1	0	0	1
Tricharaea_Sarothromyia_simplex	0&1	1	0	0	0	3	0	1	1	1	0	1	0	0	1
Tripanurga_importuna	1	2	1	0	1	1	0	1	1	1	1	1	0	0	1
Tripanurga_sp	1	2	1	0	1	1	0	1	1	1	1	1	0	0	1
Tulaeopoda_pervillosa	0	2	1	0	1	1	0	1	1	1	1	1	0	0	1
Udamopyga_iku	0	1&2	1	0	1	1	0	1	1	1	1	1	0	0	1
Villegasia_postuncinata	0	1	1	0	1	1	0	0	1	1	1	2	0	1	1
