# Supplementary material for: Museomics and phylogenomics with protein-encoding ultraconserved elements illuminate the evolution of life history and phallic morphology of flesh flies (Diptera: Sarcophagidae)
Source: BMC Ecol Evol. 2021 Apr 28;21:70. doi: 10.1186/s12862-021-01797-7 (PMC8082969; doi:10.1186/s12862-021-01797-7)

Additional file 7. Evolution of the harpes in Sarcophagidae. Ancestral character state reconstruction for harpes using maximum likelihood and the *rayDISC* function in the R package *corHMM*. Only reconstruction of the best fitting model (ARD) is shown. Pie proportions represent state probabilities estimated for each internal node. Character states are indicated in insets at the bottom.

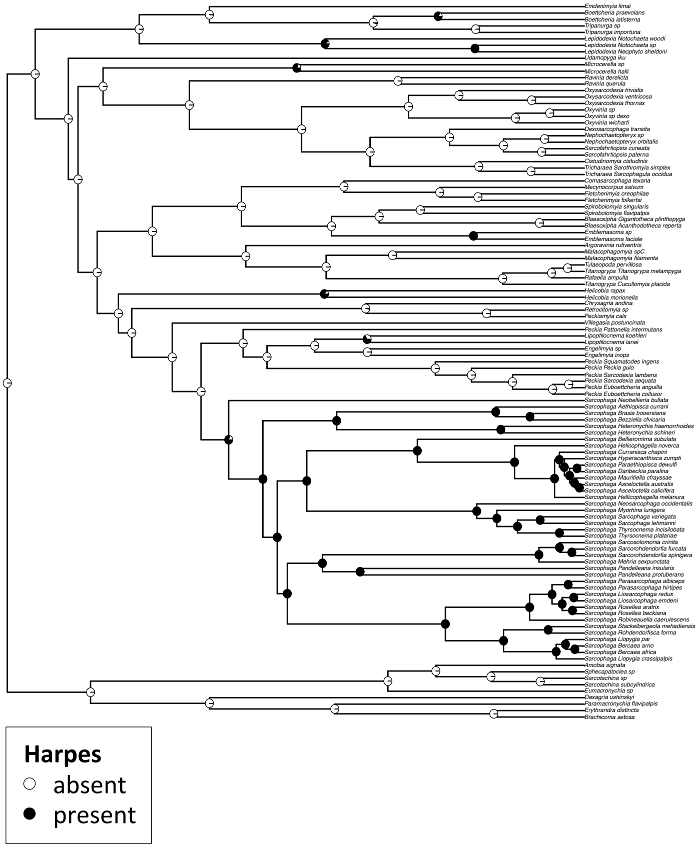

Supplement: Supplementary file 7 — Additional file 7. Evolution of the harpes in Sarcophagidae. Ancestral character state reconstruction for harpes using maximum likelihood and the rayDISC function in the R package corHMM. Only reconstruction of the best fitting model (ARD) is shown. Pie proportions represent state probabilities estimated for each internal node. Character states are indicated in insets at the bottom. [file 12862_2021_1797_MOESM7_ESM.pdf]
