# Supplementary material for: Museomics and phylogenomics with protein-encoding ultraconserved elements illuminate the evolution of life history and phallic morphology of flesh flies (Diptera: Sarcophagidae)
Source: BMC Ecol Evol. 2021 Apr 28;21:70. doi: 10.1186/s12862-021-01797-7 (PMC8082969; doi:10.1186/s12862-021-01797-7)

Additional file 10. Evolution of the phallotrema position with regard to phallic tube in Sarcophagidae. Ancestral character state reconstruction for phallotrema position with regard to phallic tube using maximum likelihood and the *rayDISC* function in the R package *corHMM*. Only reconstruction of the best fitting model (ER) is shown. Pie proportions represent state probabilities estimated for each internal node. Character states are indicated in insets at the bottom.

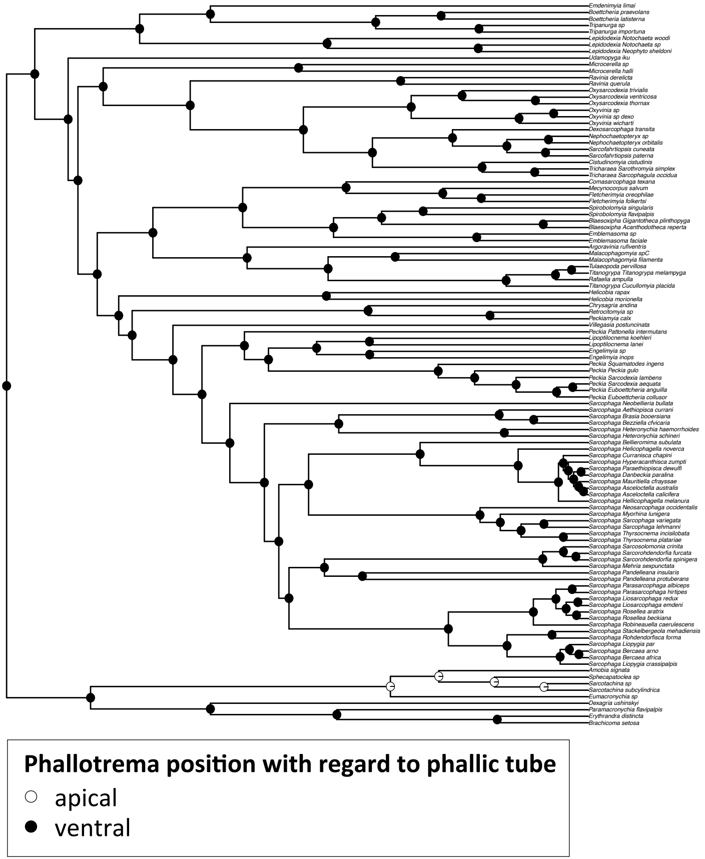

Supplement: Supplementary file 10 — Additional file 10. Evolution of the phallotrema position with regard to phallic tube in Sarcophagidae. Ancestral character state reconstruction for phallotrema position with regard to phallic tube using maximum likelihood and the rayDISC function in the R package corHMM. Only reconstruction of the best fitting model (ER) is shown. Pie proportions represent state probabilities estimated for each internal node. Character states are indicated in insets at the bottom. [file 12862_2021_1797_MOESM10_ESM.pdf]
